# Fungal community profiles in agricultural soils of a long-term field trial under different tillage, fertilization and crop rotation conditions analyzed by high-throughput ITS-amplicon sequencing

**DOI:** 10.1371/journal.pone.0195345

**Published:** 2018-04-05

**Authors:** Loreen Sommermann, Joerg Geistlinger, Daniel Wibberg, Annette Deubel, Jessica Zwanzig, Doreen Babin, Andreas Schlüter, Ingo Schellenberg

**Affiliations:** 1 Institute of Bioanalytical Sciences (IBAS), Anhalt University of Applied Sciences, Bernburg, Saxony-Anhalt, Germany; 2 Center for Biotechnology (CeBiTec), Genome Research of Industrial Microorganisms (GRIM), Bielefeld University, Bielefeld, North Rhine-Westphalia, Germany; 3 Department of Agriculture, Ecotrophology and Landscape Development, Anhalt University of Applied Sciences, Bernburg, Saxony-Anhalt, Germany; 4 Institute for Epidemiology and Pathogen Diagnostics, Julius-Kühn-Institut–Federal Research Centre for Cultivated Plants (JKI), Braunschweig, Lower Saxony, Germany; University of California Riverside, UNITED STATES

## Abstract

Fungal communities in agricultural soils are assumed to be affected by soil and crop management. Our intention was to investigate the impact of different tillage and fertilization practices on fungal communities in a long-term crop rotation field trial established in 1992 in Central Germany. Two winter wheat fields in replicated strip-tillage design, comprising conventional *vs*. conservation tillage, intensive *vs*. extensive fertilization and different pre-crops (maize *vs*. rapeseed) were analyzed by a metabarcoding approach applying Illumina paired-end sequencing of amplicons generated by two recently developed primer pairs targeting the two fungal Internal Transcribed Spacer regions (ITS1, ITS2). Analysis of 5.1 million high-quality sequence reads uncovered a diverse fungal community in the two fields, composed of 296 fungal genera including 3,398 Operational Taxonomic Units (OTUs) at the 97% sequence similarity threshold. Both primer pairs detected the same fungal phyla (*Basidio*-, *Asco*-, *Zygo*-, *Glomero*- and *Chytridiomycota*), but in different relative abundances. OTU richness was higher in the ITS1 dataset, while ITS2 data were more diverse and of higher evenness. Effects of farming practice on fungal community structures were revealed. Almost two-thirds of the fungal genera were represented in all different soil treatments, whereas the remaining genera clearly responded to farming practice. Principal Component Analysis revealed four distinct clusters according to tillage practice and pre-crop. Analysis of Variance (ANOVA) substantiated the results and proved significant influences of tillage and pre-crop, while fertilization had the smallest and non-significant effect. In-depth analysis of putative phytopathogenic and plant beneficial fungal groups indicated distinct responses; for example *Fusarium* was significantly enriched in the intensively fertilized conservation tillage variants with the pre-crop maize, while *Phoma* displayed significant association with conventional tillage and pre-crop rapeseed. Many putative plant beneficial fungi also reacted differentially to farming practice with the most distinct responders identified among the *Glomeromycota* (arbuscular mycorrhizal fungi, AMF).

## Introduction

Fungi are involved in several soil functions like decomposition of organic material, nutrient cycling, mineral mobilization, formation of soil aggregates, elevated water holding capacity, plant growth promotion and suppression of phytopathogens. Another beneficial feature of especially mutualistic root endophytic fungi is the induction of systemic resistance in host plants *via* ethylene/jasmonate or salicylic acid pathways and thereby increasing tolerance levels of crops to biotic and abiotic stress factors [1]. However, many fungi are also plant pathogens that reside either in soil (soil-borne) or persist on organic debris. The composition of fungal soil communities is influenced by several factors like soil type, physicochemical structure, canopy, plant communities and geo-climatic conditions [2]. Compared to undisturbed natural ecosystems, fungal soil communities in agro-ecosystems are exposed to additional influencing factors related to soil and crop management practices. The impact of different agricultural management regimes on fungal community composition gains rising interest, although, up to date, only few studies were dedicated to determine the effects of tillage, fertilization and crop rotation on microbial diversity [3–5].

Soils are a limited natural resource prone to erosion, degradation and contamination. Intensive land-use increases environmental impacts due to the application of agrochemicals. Overuse or inappropriate handling can cause leakage into adjacent ecosystems and contamination of surface and subterranean water. As a consequence, governments tighten legal regulations by lowering threshold values for agrochemicals and promote sustainable agricultural production procedures. Strict regulations for fertilizer and pesticide applications are announced and already show implications on national research strategies focusing on more extensively managed farmland. Several research programs have been launched to safeguard soil and its long-term economic productivity. Outstanding programs are the Agroscope Program on soil biodiversity of the National Soil Survey (NABO) of Switzerland (www.bafu.admin.ch/bodenschutz), the Soil as Sustainable Resource Program (BonaRes) of the Ministry of Research and Education (www.bonares.de), Germany, and the Sustainable Agriculture Research and Education Program (SARE) of the US Department of Agriculture (www.sare.org).

Analyses of soil-related data to assess soil properties like fertileness, stability and resilience progressively include information about soil microbiology. Commonly, microbial community profiling is addressed by PCR amplification of valid and universal phylogenetic marker sequences and subsequent high-throughput amplicon sequencing on state-of-the-art sequencing platforms. Thus, microbiomes existing in the guts of humans and animals [6], in soil or in the rhizosphere and phyllosphere of plants [7] could be characterized by applying this approach. To resolve fungal community structures, primers targeting the Internal Transcribed Spacer regions ITS1 and ITS2, which are located between rRNA genes in eukaryotes, are routinely applied for amplicon generation. Several studies compared the information content of ITS1 and ITS2 sequences, but results are ambiguous. The ITS2 region was suggested to be more variable than ITS1 [8], but many studies include both ITS regions to avoid underestimation of diversity in the sampled communities [9,10]. An established method to examine fungal diversity is paired-end sequencing of PCR amplicons on the Illumina MiSeq platform and it has proven to reliably reflect fungal diversity from environmental samples. Former studies analyzed mycobiomes from plants, soil [11], decaying organic material as well as aquatic and marine environments [12]. The information content of each single sequence from NGS approaches is limited due to short read lengths, which does not allow for unambiguously identifying fungal species, but is sufficient to identify fungal taxa at the genus level. Sequence clustering is utilized, typically at a threshold of 97% sequence identity, to group sequences into unique operational taxonomic units (OTUs). For taxonomic assignment of fungal OTUs, one of the most comprehensive databases is UNITE [13].

In this study, which was conducted within the DiControl Project (http://dicontrol.igzev.de) of the BonaRes Program, we investigated root-proximate soil layers of two winter wheat fields that are part of a 6 ha large long-term strip-tillage field trial established in Central Germany in 1992. Long-term trials receive high priority over short-term changes in land management practices, because differences in soil microbial communities are expected to be more pronounced and sharply distinguished over time. The main goal of this study is to identify soil and crop management strategies that favor plant beneficial fungi and constrain plant pathogens in terms of soil health and productivity. Each investigated winter wheat field divides in two sub-plots that represent conventional tillage *vs*. conservation tillage. Sub-plots further divide in sub-sub-plots that are managed by intensive *vs*. extensive N-fertilization. The two wheat fields differed in the pre-crop (maize *vs*. rapeseed) according to the crop rotation scheme. Obtained amplicon sequencing data were used for a comparative study to elucidate the effects of tillage, N-fertilization and pre-crops on fungal community structures. Furthermore, the information content of ITS1 *vs*. ITS2 analysis was compared and the consistency of results from the different field replicates examined. Additionally, the impact of our representative sample collection method, sample preparation procedure and PCR strategy for ITS-amplicon generation was evaluated.

## Materials and methods

### Long-term field trial setting and site conditions

Investigations were made in winter wheat plots of a long-term field trial started 1992 in Bernburg, Saxony Anhalt, Germany (51°82’ N, 11°70’ E, 80 m above sea level). The soil is a loess chernozem over limestone with an effective rooting depth of 100 cm, containing 22% clay, 70% silt and 8% sand in the ploughed upper (Ap) horizon. It has a neutral pH (7.0–7.4) and an appropriate P and K supply (45–70 mg kg^-1^ and 130–185 mg kg^-1^ in calcium-acetate-lactate extract, respectively). The 1980–2010 annual average temperature was 9.7°C, the average annual precipitation 511 mm.

**Fig 1:**
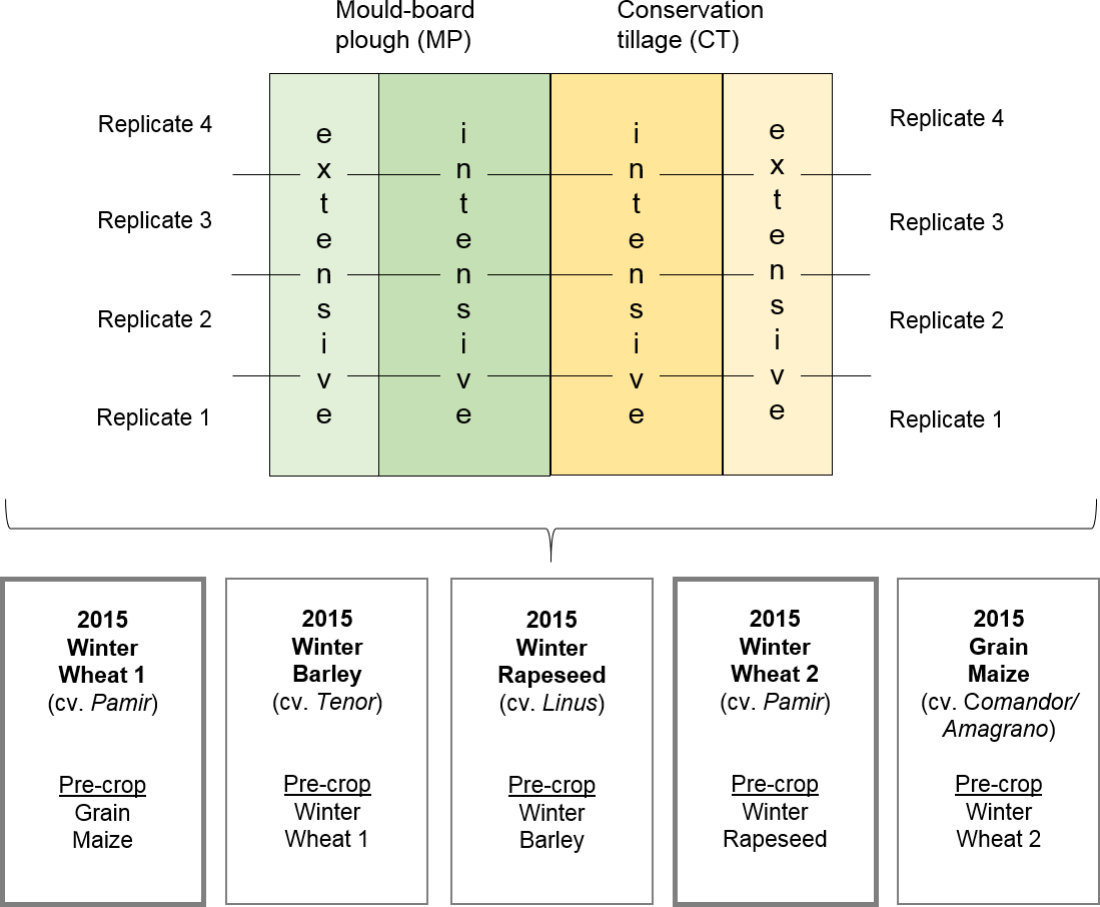
Schematic diagram of the long-term field trial. Experimental design: strip-split-plot design with five fields of crop rotation as main plots (lower panel, rotation cycle from right to left). Management practice (upper panel) for each main plot: tillage MP (left half) and CT (right half) as sub-plots and N-fertilization (int/ext) as sub-sub-plots in four replicates.

On five large parcels (1.2 ha each, main plots) the individual crops–grain maize (*Zea mays*)–winter wheat (*Triticum aestivum*)–winter barley (*Hordeum vulgare*)–winter rapeseed (*Brassica napus* ssp. *napus*)–winter wheat–are rotated (Fig 1). All crop residues remain on the fields. Conservation tillage cultivator (CT, 10 cm flat non-inversion soil loosening) is compared to sub-plots with conventional tillage (MP; mould-board plough, carrier board with combined alternating ploughshares, ploughing depth 30 cm, incl. soil inversion). The differentially managed soils are either intensively (int) operated according to usual practice regarding N supply and pesticide application or extensively managed (ext; reduced N supply, without fungicides and growth regulators). Winter wheat variety “Pamir” has been sown on 06 and 29 October 2014 after rapeseed or maize, respectively. N fertilization was carried out with 220 or 90 kg ha^-1^ N as ammonium sulphate + calcium ammonium nitrate in intensive or extensive treatments, respectively. Fungicides (S1 Table) were used in intensive treatments only: 1.25 L ha^-1^ Credo (100 g L^-1^ picoxystrobin+ 500 g L^-1^ chlorthalonil, DuPont, Germany) + 1.25 L ha^-1^ Opus Top (83.7 g L^-1^ epoxiconazole + 250 g L^-1^fenpropimorph, BASF, Switzerland) at start of stem elongation (BBCH 31; BBCH code, Biologische Bundesanstalt, Bundessortenamt, Chemische Industrie) as well as 1.8 L ha^-1^ Amistar Opti (400 g L^-1^ chlorthalonil+ 80 g L^-1^ azoxystrobin, Syngenta, Germany) + 0.6 L ha^-1^ Gladio (375 g L^-1^ fenpropidin+125 g L^-1^ propiconazole+125 g L^-1^ tebuconazole, Syngenta, Germany) at ear-swelling in BBCH 49. Second application was combined with 1.0 L ha^-1^ Yara Vita (micronutrient fertilizer with 50 g L^-1^ Cu, 150 g L^-1^ Mn and 80 g L^-1^ Zn, Yara, Germany). Growth regulator CCC_720_ (1 L ha^-1^, 720 g L^-1^ chlormequatchloride, Bayer, Germany) was applied at BBCH 30 in intensive treatments. Herbicides and insecticides were applied in all treatments if necessary. Winter wheat was harvested on 30 July 2015. The Bernburg field trial is located and maintained on Anhalt University owned land. Therefore, no specific permissions were required for the indicated research activities. Occurrence of protected or endangered species has never been reported for this agricultural site.

### Soil sampling and DNA isolation

Soil samples of winter wheat field 1 (WW1) with pre-crop maize and winter wheat field 2 (WW2) with pre-crop rapeseed were collected directly after harvest at a depth of 0 to 25 cm using a soil corer. Soil was densely rooted (row width approx. 12.5 cm), root mats were drilled and part of the core sample. In summary, soils with eight different farming histories à 4 replicates were collected (MP *vs*. CT, int *vs*. ext for WW1 and WW2, respectively). Each replicate was represented by 15 soil cores. For homogenization, soil was sieved using 4 mm mesh width to remove root debris and stones. Total DNA was extracted from 1 g of soil in portions of 250 mg by using two different DNA isolation kits. The PowerSoil® DNA Isolation Kit (MoBio, CA, USA) and the FastDNA™ SPIN Kit for Soil (MP Biomedicals, Germany) were utilized equally to avoid bias. DNA was pooled and quality checked on 0.8% agarose gels. Subsequently, aliquots of the isolated DNA were further purified using the Gene Clean Spin Kit (MP Biomedicals). Concentrations of purified DNAs were determined with a Qubit® 3.0 Fluorometer (Invitrogen, CA USA).

### PCR conditions

To avoid over-amplification of specific PCR-fragments and loss of detectable biodiversity, the beginning of the exponential PCR phases with soil DNA templates and ITS1 and ITS2 primers were determined by qPCR using 1x SYBR® Green Nucleic Acid Stain (Lonza, Switzerland). Each reaction consisted of 20 μl including 2x Phusion High-Fidelity PCR Master Mix (Thermo Fisher Scientific, Germany) containing 1.5 mM MgCl_2_, 200 μM of each dNTP, 0.5 μM of each primer, 0.4 U of Phusion DNA Polymerase and 20 ng of template DNA. Reactions were performed in a Piko Real 96 thermal cycler (Thermo Fisher Scientific) with the following conditions: initial denaturation at 94°C for 5 min, followed by 37 cycles at 94°C for 15 sec, 56°C for 25 sec and 72°C for 20 sec. According to qPCR results, the cutoffs for the following preparative PCRs were set in the middle of the exponential phases, which were 22 cycles for ITS1 and 21 cycles for ITS2 primers (S2 Table). Preparative PCRs were performed with sample-specific barcoded NGS primers (Metabion, Germany) using the standard eight nucleotide Illumina barcodes and the primer pairs ITS1-F_KYO2 (5’-TAGAGGAAGTAAAAGTCGTAA-3’) and ITS86R (5’-TTCAAAGATTCGATGATTCAC-3’) for the ITS1 region and ITS86F (5’-GTGAATCATCGAATCTTTGAA-3’) and ITS4 (5’-TCCTCCGCTTATTGATATGC-3’) for the ITS2 region [14–16]. To prevent stoichiometric effects, PCRs were performed at three different annealing temperatures, by using the optimal annealing temperature at 56°C and by 2°C elevated and lowered temperatures, which were 54°C and 58°C, respectively. In summary, per sample 15 PCRs were performed as technical replicates per ITS region (9 PCRs at optimal and 3 PCRs at each of the altered annealing temperatures). ITS fragments were amplified in a Basic & Gradient Labcycler (Sensoquest, Germany) by applying the following conditions: initial denaturation at 94°C for 5 min, followed by 22 (ITS1) / 21 (ITS2) cycles at 94°C for 15 sec, annealing at 54°C, 56°C or 58°C, respectively, for 25 sec and 72°C for 20 sec, followed by a final elongation step at 72°C for 7 min. PCRs were carried out in 25 μl volumes using 2x Phusion High-Fidelity PCR Master Mix (Thermo Fisher Scientific). Each reaction contained 1.5 mM MgCl_2_, 200 μM of each dNTP, 0.5 μM of each primer, 0.5 U of Phusion DNA polymerase and 25 ng template DNA.

### Preparation of sequencing pools

Subsequently, amplicons originating from the same barcode primers were pooled and the concentrations of the 64 pools (32 pools for ITS1 and 32 pools for ITS2) were determined using a Qubit® 3.0 Fluorometer (Invitrogen). Aliquots were mixed with 6x Roti^®^-Load DNA-orange I Dye (Roth, Germany) to track the samples during gel electrophoresis using low-melt agarose (1.5%) and 150 ng GeneRuler 50 bp Ladder (Peqlab, Germany) mixed with 1.5 μl Midori Green Direct Stain (Nippon Genetics Europe, Germany). Amplicons for sequencing were not stained due to possible negative effects on the following DNA sequencing reaction. Unstained amplicons were excised from the gel according to migration of the separately stained and visualized DNA size markers (molecular weight range from 200–500 bp). Gel slices were purified using the MinElute Gel Extraction Kit (Qiagen) and were finally eluted in 12 μl 10 mM Tris·HCl, pH 8.5. Concentrations of each sample were determined using a Qubit® 3.0 Fluorometer (Invitrogen). Amplicon samples were pooled in equimolar amounts separately for ITS1 and ITS2 and diluted to 10 nM. Methods have been deposited at protocols.io: 10.17504/protocols.io.nmgdc3w

### Library preparation and DNA sequencing

Fungal ITS1 and ITS2 amplicon pools were further processed as follows: To determine PCR fragment sizes, aliquots of the pooled amplicons were analyzed on a Bioanalyzer using a DNA 1000 Chip (Agilent, CA, USA). DNA concentrations were measured by means of the Quant-iT™ PicoGreen® dsDNA Assay Kit (Invitrogen, CA, USA) on an Infinite Reader M200 instrument (Tecan, Switzerland). For each amplicon library preparation 100 ng DNA were processed applying the TruSeq Nano DNA Sample Preparation Kit (Illumina, CA, USA) according to the protocol provided by the manufacturer. This step also included sequencing adapter ligation. Sequencing libraries were quantified as described above and then sequenced on a MiSeq System (Illumina) in paired-end mode (2 x 300 bp sequencing cycles) using the MiSeq Reagent Kit v3 (Illumina) following manufacturer’s instructions. For each indexed ITS amplicon pool, 25% of a flow cell was provided. Raw sequencing data are available in the EBI/NCBI/DDBJ database under the project accession number PRJEB19896.

### Bioinformatics and taxonomic assignment

After sequencing, raw data were imported into an in-house processing pipeline [17] for sequencing primer and barcode trimming. Data analysis was conducted by separation and amplicon barcode trimming of pooled ITS1 and ITS2 datasets. This processing step was performed based on the FASTX toolkit (http://hannonlab.cshl.edu/fastx_toolkit/). Separated datasets were further processed and analyzed by a pipeline as recently described [18–20]. Briefly, raw sequences were merged by FLASH [21]. Sequences with > 1 N (ambiguous bases) in the read and expected errors > 0.5 were discarded. The molecular identifier tags and primer sequences were removed allowing 0 and 2 mismatches, respectively. The software package UPARSE was applied for de-noising and chimera detection [22]. Operational taxonomic units (OTUs) were defined at 97% sequence similarity by applying the program USEARCH 8.1 [23]. Processed OTUs were taxonomically classified using the RDP classifier 2.12 [24] with the UNITE database v7.0 [13,25]. Only hits featuring a confidence value of at least 0.8 (phylum rank) were considered. Finally, raw sequences were mapped to the OTU sequences to receive quantitative assignments. Identified genera were compared to entries in the “List of Plant Diseases” published by The American Phytopathological Society. The lists of wheat (actual crop) and rapeseed diseases (pre-crop) were selected (www.apsnet.org/publications/commonnames/Pages/default.aspx) and names of the fungal pathogens (anamorph, teleomorph and synonymous names) adjusted with the index fungorum website: http://www.speciesfungorum.org/Names/Names.asp. Maize diseases were neglected since no evidence for fungal infections was reported for the experimental site analyzed.

### Statistical analysis

Factorial ANOVA for wheat yield was calculated using SPSS Statistics v22 (IBM, Germany). Least significant differences (LSDs) with *p*≤0.05 for a strip-split-plot design were estimated according to Thomas [26]. All OTUs recovered from ITS1 and ITS2 datasets were subjected to principle component analysis (PCA) using ClustVis [27]. For hierarchical clustering based on “City Block” distance and average linkage, Cluster 3.0 was used [28]. PCA was conducted to further describe the similarity between the fungal communities of different samples. Differentially abundant OTUs were identified by application of the metagenomeSeq Bioconductor package (v1.1.16), [29]. The approach implements a novel normalization technique and a statistical model addressing under-sampling in high-throughput sequencing projects. The bias in the calculation of differential abundance introduced by total-sum normalization is corrected by cumulative sum scaling (CSS) [29]. Secondly, under-sampling of microbial communities is reassessed by implementation of a zero-inflated Gaussian distribution mixture model [29]. The first step was a non-parametric permutation test on t-statistics. From each replicate, 40,000 sequence reads were randomly picked in 100 permutations followed by calculation of average diversity indices considering all permutations. Secondly, a non-parametric Kruskal-Wallis test was performed followed by the Wilcox rank-sum tests on subgroups to avoid positive discoveries of differentially abundant features driven by potential cofounders. PermANOVA was performed using PAST [30] and the Shannon and Simpson indices were calculated based on in-house perl scripts inside the pipeline. Venn diagrams were created with Venny [31]. The analyses of significant differences in the datasets were calculated with SigmaPlot (Systat Software, San Jose, CA). For selected putative phytopathogenic or plant beneficial fungal genera raw data were transformed into ranks to achieve normalized distribution for Three-Way-ANOVA (Shapiro-Wilk, p = 0.05; Brown-Forsythe, p = 0.05) with post-hoc test Holm-Sidak (p = 0.05). Krona plots (S1–S16 Files) to zoom into taxonomic assignments of treatment replicates were designed by using the interactive metagenomic visualization web browser [32].

## Results

### Long-term influence of agricultural management on winter wheat yield

**Fig 2:**
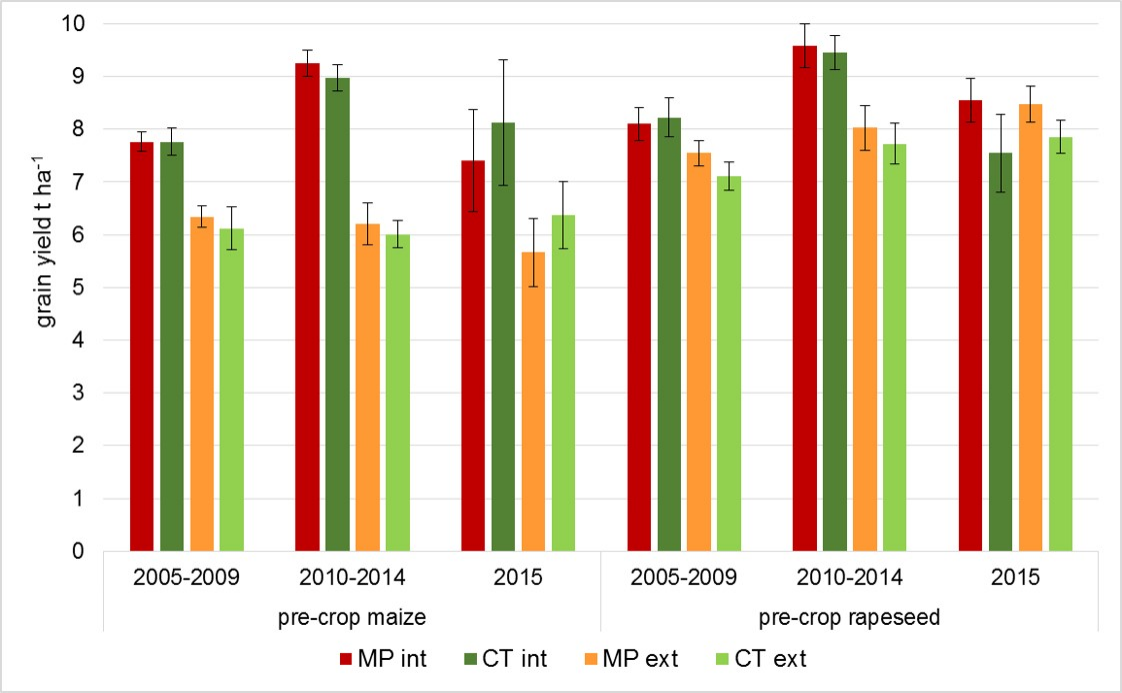
Wheat grain yield depending on pre-crop and soil management. MP, mould-board plough *vs*. CT, conservation tillage cultivator and int, intensive *vs*. ext, extensive N fertilization.

In long-term average (2005–2014) crop yield from the long-term field trial was comparable in both tillage treatments, but varied within single years depending on weather conditions. Extensive production resulted in significant yield reduction, especially in wheat with maize as pre-crop. Wheat grain yield of the analyzed two crop rotations in long-term averages and the sampling year 2015 are presented in Fig 2.

Wheat grain yield increased from period 2005–2009 to period 2010–2014 in intensive treatments because of improved crop varieties and an increase of N supply to 220 kg ha^-1^. Within the same pre-crop and intensity setting, tillage had only minor effects. Pre-crop rapeseed resulted in higher wheat yield, especially in extensive treatments. Lower N supply and shortened pesticide use reduced the wheat grain yield with maize as pre-crop considerably. This effect was much lower in case of rapeseed as pre-crop, but still reached significant levels (Table 1).

**Table 1:** Least Square Differences (LSD) of diverse soil management effects.

	2005–2009	2010–2014	2015
LSD (p<0.05) effect preceding crop	0.42	0.30	1.10
LSD (p<0.05) effect tillage	0.52	0.21	0.87
LSD (p<0.05) effect fertilization intensity	0.44	0.22	0.90

Effects of pre-crop, tillage and fertilization in the long term (2005–2009 and 2010–2014) and in the sampling year 2015. Because of multiple interactions between experimental factors, LSDs were calculated for comparison of A×B×C means on the same level of other factors only.

Growing season 2015, when sampling took place, was characterized by a strong water deficit from February to June, but above average precipitation in July. With 7.8 and 8.0 t ha^-1^ (in intensive treatments after maize and rapeseed, respectively) grain yield was about 15% lower than the average of former five years with increased variability between the replicates. Tillage and pre-crop had no significant effect. Extensive treatments reached middle-rate grain yield of 8.2 and 6.0 t ha^-1^ after rapeseed or maize, respectively. The difference to intensive treatments was only significant with maize as pre-crop (Fig 2). Physicochemical soil properties were described previously [33]. Basic soil parameters are presented in S3 Table.

Common fungal diseases observed visually in the investigated winter wheat fields during the last years were, besides Fusariosis, eyespot (*Pseudocercosporella herpotrichoides* (Fron) Deighton, syn. *Oculimacula yallundae* Crous & W. Gams), take-all disease (*Gaeumannomyces graminis* (Sacc.) Arx & D.L. Olivier, syn. *Ophiobolus graminis* Sacc.), sharp eyespot (*Rhizoctonia cerealis* E.P. Hoeven, syns. *Ceratobasidium cereale* D.I. Murray & Burpee, *Ceratorhiza cerealis* (E.P. Hoeven) R.T. Moore), stripe rust / yellow rust (*Puccinia striiformis* Westend.), *Septoria* blotch (*Septoria tritici* Berk. & M.A. Curtis, syn. *Mycosphaerella graminicola* (Fuckel) J. Schröt.), *Stagonospora* blotch (*Septoria nodorum* Berk., syn. *Para*s*tagonospora nodorum* (Berk.) Castell. & Germano) and yellow leaf spot / tan spot (*Helminthosporium tritici-repentis* Died., syns. *Drechslera tritici-vulgaris* (Y. Nisik.) S. Ito, *Pyrenophora tritici-repentis* (Died.) Drechsler). Concerning the pre-crops of the two winter wheat fields, fungal infections caused only minor problems in maize cultivation (no fungicides, only insecticides are routinely applied). For the pre-crop rapeseed, the most important fungal diseases observed were stem canker (*Phoma lingam* (Tode) Desm.), stem-rot (*Sclerotinia sclerotiorum* (Lib.) De Bary, syn. *Peziza sclerotiorum* Lib.), wilt (*Verticillium longisporum* (C. Stark) Karapapa, Bainbr. & Heale), as well as black leg (*Alternaria brassicae* (Berk.) Sacc.), and grey-mold (*Botrytis cinerea* Pers., syn. *Botryotinia fuckeliana* (de Bary) Whetzel).

### Fungal community profiling by high-throughput ITS1 and ITS2 amplicon sequencing

According to the deployed sequencing barcodes, 64 datasets were obtained, 32 datasets for ITS1 and ITS2 amplicons, respectively. Each of the 32 data subsets represented four replicates of the eight investigated soil treatments including mould-board plough *vs*. conservation tillage (cultivator) and intensive *vs*. extensive fertilization in the two winter wheat fields WW1 (pre-crop maize) and WW2 (pre-crop rapeseed). Roughly, between 60,000 and 120,000 sequences were obtained per sample. This demonstrated that the equimolar pooling procedures worked satisfactory and no serious discrepancies occurred in the datasets. About 10% of all sequences could not be correctly matched during paired-end read merging. Another 20% of the sequences were discarded due to incorrect bases in primers and/or barcodes and by chimera filtering. All rarefaction curves were close to saturation (S1 Fig), which indicated that the great majority of taxonomic diversity was covered at this sequencing depth. After quality filtering, roughly 5.1 Mio high quality reads remained. Approximately, 2.85 Mio reads were obtained for the ITS1 amplicons and approx. 2.25 Mio reads were achieved for the ITS2 amplicons, respectively (S4 Table). ITS1 and ITS2 primer specificities to detect fungal sequences in environmental samples were in both cases >99.8%. The size distribution of the obtained amplicons was between 269 bp and 388 bp (with the majority of reads at 325 bp) for the ITS1 fragments and between 200 bp and 390 bp (majority of reads at 298 bp) for the ITS2 amplicons.

### Clustering of fungal OTUs

**Fig 3:**
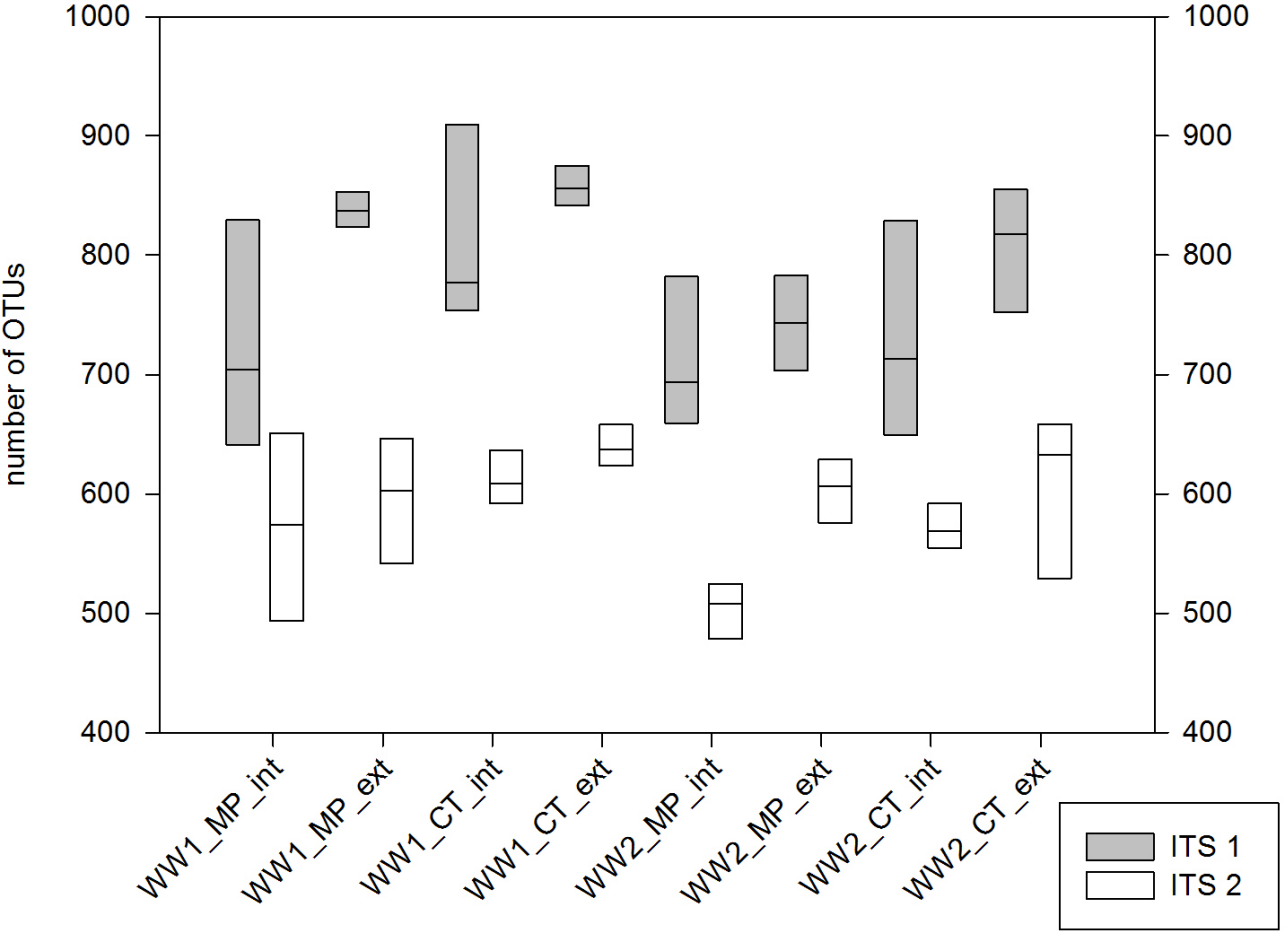
Fungal OTU richness according to agricultural management practice. Median values for the numbers of OTUs in the 4 replicates per treatment were calculated for the pre-crop maize (WW1), pre-crop rapeseed (WW2), regarding mould-board plough (MP) *vs*. conservation tillage cultivator (CT), extensive (ext) and intensive (int) N fertilization.

All high quality reads were subjected to cluster analysis based on 97% sequence similarity and classified with the UNITE database. All obtained OTUs presented in S5 and S6 Tables (2,000 OTUs for ITS1 and 1,398 OTUs for ITS2, p<0.001) could be assigned to the kingdom of Fungi. ITS1 OTU richness was significantly higher in WW1 (mean 777 OTUs) compared to WW2 (mean 750 OTUs, p = 0.035). When taking ITS2 sequences into account, a similar trend was observed. Besides pre-crop, extensive and intensive fertilization exerted a significant influence on OTU richness in both wheat fields (p = 0.018). In WW1, all extensive treatments yielded around 850 ITS1 OTUs in every replicate, while in intensive treatments only two field plots reached this value (mean 770). Likewise, WW2 extensive treatments accounted for higher ITS1 OTU richness (mean 780) while in intensive treatments a mean of only 745 OTUs was found. ITS2 data also revealed significant differences between extensive *vs*. intensive treatments in the two fields. The tillage effect on alpha-diversity was not significant. The highest OTU richness was observed for ITS1 sequences from WW1 with the most consistent results in extensive treatments. This was also true for the pre-crop rapeseed, but less pronounced (Fig 3).

OTU data were utilized to calculate the Simpson and Shannon diversity indices. The Simpson Index describes the probability of two randomly chosen sequences belonging to different OTUs. By looking at the pre-crop effect, this probability was higher for WW1 (Table 2). When taking mould-board plough (MP) *vs*. conservation tillage (CT) into account, the highest probabilities were found for CT, especially in WW1. In both cases, ITS2 data were clearly more diverse than ITS1. In extensive WW1 treatments, higher diversity was also detected with ITS2 sequences, although the overall number of OTUs (1,398) and sequencing depth (2.25 Mio sequences) were significantly lower. The highest and lowest diversity in the soil mycobiome was detected with ITS2 in “WW1 CT ext” and “WW2 MP int” treatments with 0.975 and 0.902 as means of the four replicates, respectively (S7 Table).

**Table 2:** Fungal community diversity indices according to Simpson and Shannon.

Treatment	ITS Region	Field	Simpson-Index	Std. Deviation	Shannon-Index	Std. Deviation
**Pre-cop (all variants)**	ITS1	WW1	0.946	0.023	3.94	0.22
		WW2	0.935	0.024	3.76	0.26
	ITS2	WW1	0.954	0.036	4.12	0.36
		WW2	0.917	0.066	3.73	0.48
**Plough (MP)**	ITS1	WW1	0.953	0.013	3.97	0.20
		WW2	0.937	0.031	3.81	0.34
	ITS2	WW1	0.939	0.045	3.95	0.43
		WW2	0.905	0.061	3.59	0.47
**Cultivator (CT)**	ITS1	WW1	0.938	0.029	3.90	0.25
		WW2	0.933	0.015	3.72	0.17
	ITS2	WW1	0.970	0.009	4.30	0.13
		WW2	0.929	0.072	3.88	0.47
**Intensive**	ITS1	WW1	0.954	0.007	3.99	0.15
		WW2	0.925	0.027	3.66	0.28
	ITS2	WW1	0.956	0.015	4.10	0.18
		WW2	0.926	0.056	3.80	0.44
**Extensive**	ITS1	WW1	0.937	0.030	3.88	0.27
		WW2	0.945	0.015	3.87	0.21
	ITS2	WW1	0.953	0.050	4.15	0.49
		WW2	0.908	0.077	3.66	0.53

The pre-crop effect was calculated for ITS1 and ITS2 separately and based on 16 values for each pre-crop (two tillage and two fertilization treatments, four replicates each). Effects of the other factors were based on eight values per ITS region and field, e.g. effect intensive (two tillage practices, four replicates each). WW1: winter wheat pre-crop maize, WW2: winter wheat pre-crop rapeseed. Sequences were normalized with metagenome Seq [29].

The Shannon Index determines OTU quantities and sequence abundances and is a measure for the evenness of sequence distribution among OTUs. Analysis revealed medium evenness for all soil treatments ranging between 3.61 and 4.31 (Table 2). Noteworthy, in almost all comparisons between ITS1 and ITS2 data, sequences were more evenly distributed in the ITS2 dataset. The only exceptions were the MP variants, with almost identical indices. The highest value was obtained for “WW1 CT ext” treatments with 4.39 on average in the ITS2 dataset. The lowest diversity was determined in “WW2 MP int” treatments with 3.58 (S7 Table).

### Taxonomic assignments based on ITS1 and ITS2 sequences

**Fig 4:**
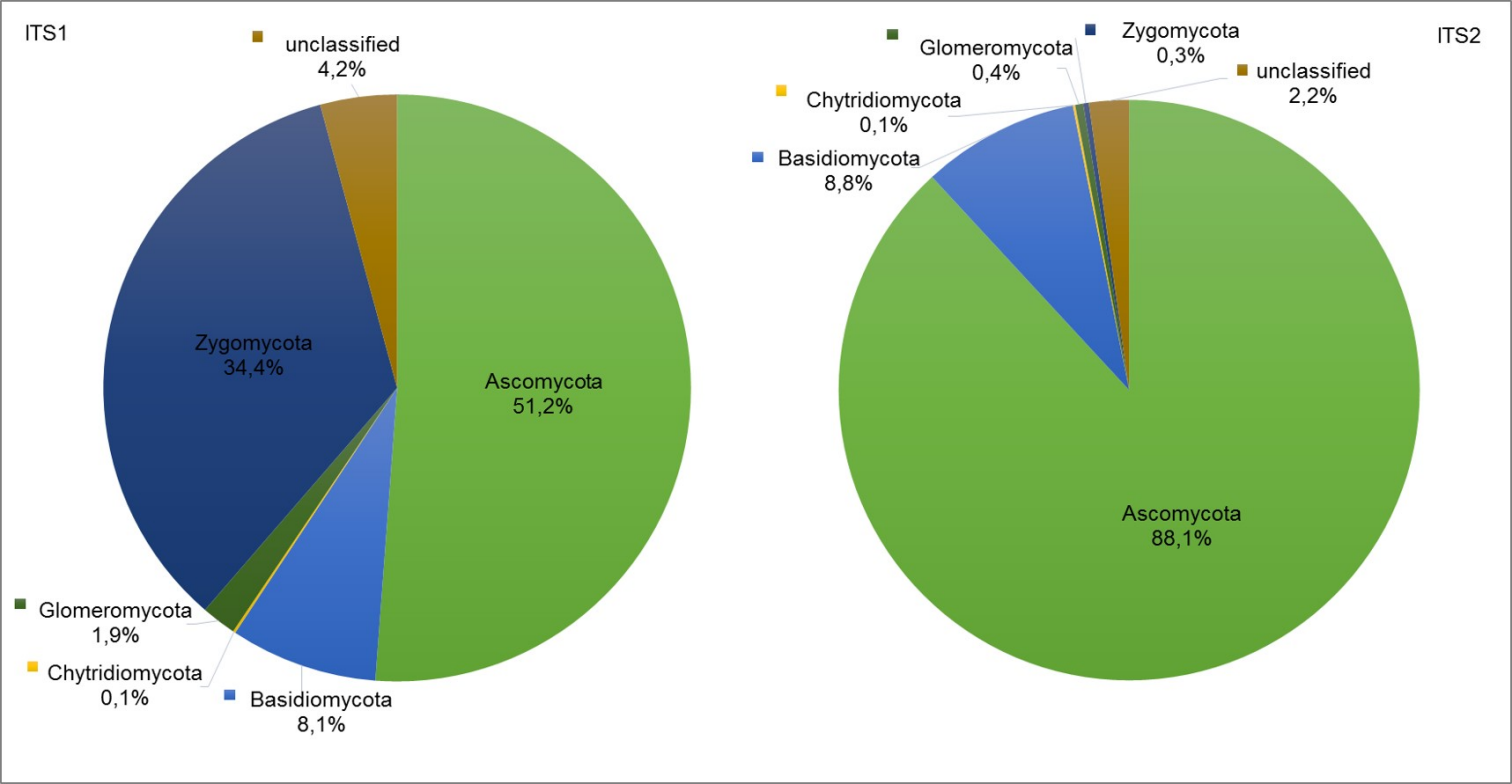
**Ratios of detected fungal phyla by applying the ITS1 (left) and ITS2 primers (right).** ITS1 sequences were more evenly distributed across the different phyla, while ITS2 sequences featured higher resolution for characterization of the *Ascomycota*.

PCR amplification with the primer combinations for the ITS1 and the ITS2 region resulted in the detection of five fungal phyla (Fig 4). Roughly, 51% *Asco*-, 35% *Zygo*-, 8% *Basidio*-, 2% *Glomero*- and 0.1% *Chytridiomycota* were detected by the ITS1 primers (unclassified fungi 4%) and about 88% *Asco*-, 9% *Basidio*- and together 1% *Zygo*-, *Glomero*- and *Chytridiomycota* by the ITS2 primers (unclassified fungi 2%). This indicates a strong primer bias towards the *Ascomycota* in the ITS2 dataset. The *Zygomycota*, which are not a monophyletic group, displayed the strongest differences in the two datasets (ITS1 34.6% and ITS2 0.3% *Zygomycota*, respectively). These included within the ITS1 data the orders *Entomophthorales*, *Basidiobolales*, *Mortierellales* and *Mucorales* (54 OTUs in total), whereas within the ITS2 sequences only the latter two orders were identified (14 OTUs in total). Additionally, the relative abundance of the *Glomeromycota* differed widely between the ITS1 and ITS2 datasets. The number of sequences belonging to the *Glomeromycota* within the ITS1 data was almost five times higher than within ITS2 sequences (1.9% *vs*. 0.4% *Glomeromycota*, respectively).

Over all phyla, there were 20 orders, 35 families and 212 genera based on ITS1 sequences and within the ITS2 dataset we found 18 orders, 47 families and 220 genera. In total, 76 genera were unique in the ITS1 and 84 genera in the ITS2 dataset; 136 genera were common to both and in total, 296 genera could be assigned in the two winter wheat fields. This indicates a slightly higher information content of the ITS2 amplicons, but this contributed mainly to describe the diversity among the *Ascomycota*. The ITS1 primers detected a broader range of genera in the other phyla, mainly in the *Zygo*- and *Glomeromycota*. In summary, the applied primers for ITS1 were able to detect a similar number of genera as compared to the ITS2 primers, but genera were more evenly distributed across different phyla and the ITS1 primers detected a higher portion of so far uncharacterized fungal taxa.

**Fig 5:**
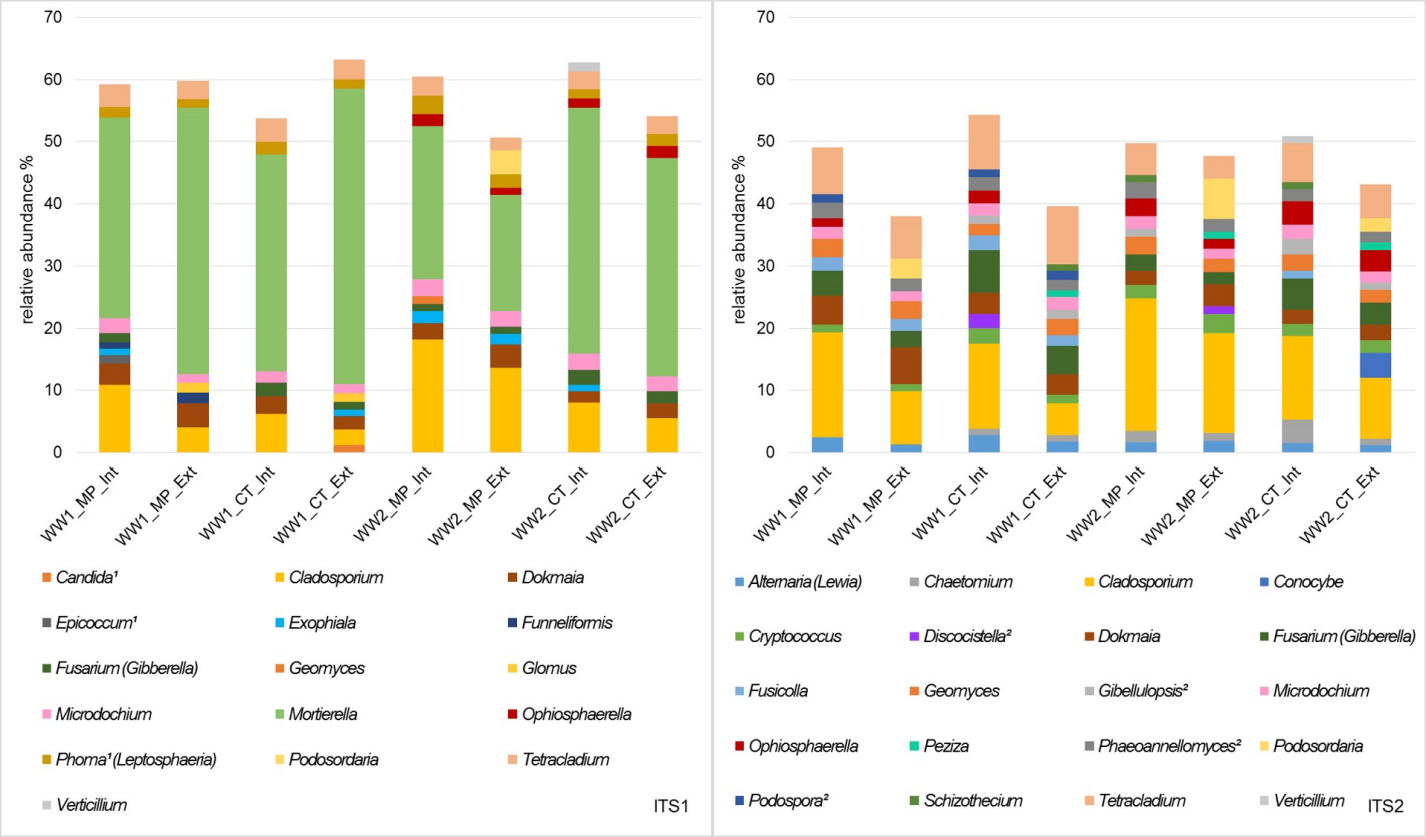
Predominant fungal genera in two winter wheat fields with different pre-crops and soil management practices. Genera are listed as median of four replicates at a minimum relative abundance threshold of 1%. ITS1 predominant genera (left) added up to 50–64% of the total genera detected, while in ITS2, predominant genera (right) accounted only for 38–55% of the total (^1^only detectable in the ITS1 and ^2^only detectable in the ITS2 dataset). For abbreviations refer to Fig 3.

Relative ratios of genera were correlated to the different soil treatments and pre-crops (Fig 5). In general, the great majority of ITS1 and ITS2 OTUs displayed relative abundances below 0.1%, followed by the OTUs with medium abundance between 0.1 and 1%. Highly abundant OTUs (1 - >10%) were rare and accounted for less than 6.57% of the total sequences (5.87% within ITS1 and 7.22% within ITS2 sequences). Highly abundant genera were distributed among three phyla in ITS1 (*Asco*-, *Zygo*- and *Glomeromycota*) and only two phyla in the ITS2 data (*Asco*- and *Basidiomycota*). The predominant genus within the ITS1 sequences was the *Zygomycete Mortierella* (represented by 27 OTUs), which accounted within WW1 for 32% to 47% of all sequences and within WW2 between 18% and 39%. The second most abundant genus was *Cladosporium* (6 OTUs) ranging between 2.5% and 10% in WW1 and between 5.5% and 18% in WW2. The third most predominant genera were *Tetracladium* and *Dokmaia*, both accounting for ca. 3% in WW1 and WW2 (based on 3 and 5 OTUs, respectively). All other genera detected by the ITS1 primers ranged below 3% with the highest values reached by *Microdochium* (2.2%), *Phoma* (1.9%) and *Fusarium*/*Gibberella* (1.6%).

Different dominant genera were found within the ITS2 data. This was mainly due to the fact that ITS2 primers rarely detected zygomyceteous fungi, which were most predominant in the ITS1 dataset. The most prevalent genus in the ITS2 dataset was *Cladosporium* (represented by only 2 OTUs), which accounted for 5% to 16.5% in WW1 and 10% to 21% in WW2, followed by the genus *Tetracladium* (represented by 8 OTUs) ranging between 7% and 9.5% in WW1 and 3.5% and 6.5% in WW2, respectively. The third most abundant genera were *Dokmaia* (3 OTUs) exhibiting relative abundances between 3.5% and 6% within WW1 and 2.5% and 3.5% in WW2, as well as *Fusarium*/*Gibberella* (represented by 9 OTUs) with values between 2.5% and 7% in WW1 and 2% and 5% in WW2, respectively. All other genera detected by the ITS2 primer pair ranged below 3% with the highest values reached by *Geomyces* (2.5%), *Phaeoannellomyces* (2.1%) and *Cryptococcus* (1.95%).

### Effects of farming practices on fungal community structures

**Fig 6:**
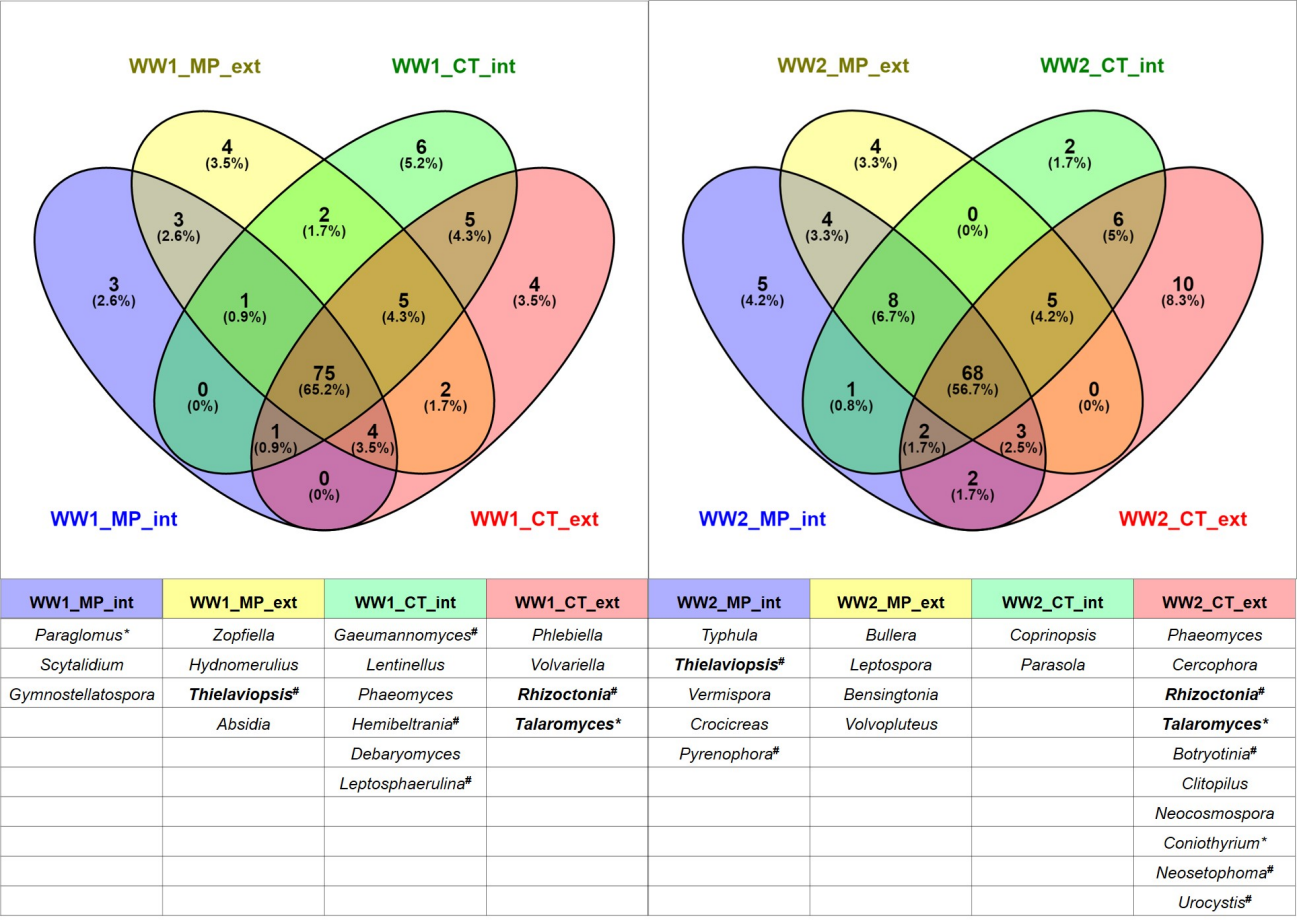
Venn diagrams depicting the numbers and percentages of fungal genera in differently managed soils. For the two winter wheat fields WW1 pre-crop maize (left) and WW2 pre-crop rapeseed (right), absolute numbers and percentages are given. Soil samples were investigated from mould-board plough intensive (MP_int), mould-board plough extensive (MP_ext), conservation tillage intensive (CT_int) and CT extensive variants (CT_ext) in four replicates. Two threshold values were applied (minimum relative abundance 0.01% and reproducibility with minimum presence in two out of four replicates). Genera are listed in descending order of relative abundance (*genera including plant beneficial or ^**#**^plant pathogenic species). Unique genera in distinct soil treatments are shown below the respective figure panels. Genera in bold appeared in both pre-crops.

Synonymous names and anamorphic *vs*. teleomorphic denominations were revised and adjusted for compilation of ITS1 and ITS2 data. For evaluating the impact of agricultural management practices on the fungal community structure, compiled ITS1 and ITS2 data were filtered for two cut-off values. The mandatory conditions chosen were (i) relative abundance (a genus had to be represented with at least 0.01% of the total sequences per treatment) and (ii) reproducibility (a genus had to be present in at least two out of four replicates). For construction of the diagrams (Fig 6), the highest and most consistent values for either ITS1 or ITS2 data were chosen to avoid adding up genera that were detected by ITS1 and ITS2, simultaneously. Analysis was performed for the two winter wheat fields separately. The applied criteria were fulfilled by 115 genera within WW1 and 120 genera within WW2. The comparisons for MP *vs*. CT as well as ext *vs*. int treatments including the names of unique fungal genera are deposited in S2 Fig.

Within WW1, 65.2% of the identified genera were detectable in all four soil treatments by applying the indicated threshold values. The remaining 34.8% showed differential appearance according to agricultural practice with none of the observed genera being common between the soil treatments MP int and CT ext, as well as among MP int and CT int variants (Fig 6, left panel). Remarkable for common genera between different soil treatments in WW1 was firstly the pathogen *Stagonospora*, which was observable in the CT int and ext variants, and secondly the plant beneficial genera *Claroideoglomus* and *Rhizophagus* (both *Glomeromycota*). The latter two were not detectable in WW1 MP int, whereas *Entrophospora* (*Glomeromycota*) and *Sebacina* (*Basidiomycota*) could not be found in WW1 CT int treatments. Between three and six unique genera were found in every soil treatment (Fig 6). In contrast to WW1, in WW2 only 56.7% of the detected genera were common to all soil treatments. The remaining 43.3% were responding to farming conditions with no common genera shared among the extensive treatments of MP and CT, as well as between the MP ext and CT int variants (Fig 6, right panel).

Common genera between the different soil treatments were also detectable in WW2. Only one genus containing phytopathogens has been found among them, *Gaeumannomyces* (take-all). As one of the most important pathogens in winter wheat, it was above the threshold in both ploughed variants as well as in CT int management in WW2. Apart from that, four plant beneficial and at the same time root-endophytic genera were among the common taxa between soil treatments. *Entrophospora* was observed exclusively in the ploughed variants, while *Diversispora* was above the threshold only in the CT variants (both *Glomeromycota*). *Claroideoglomus* (*Glomeromycota*) was found in all treatments except MP int and *Sebacina* (*Basiodiomycota*) was the only common genus in all intensive treatments of WW2. Notably, in WW2 between two and ten unique genera were exclusively detectable in single soil treatments.

**Fig 7:**
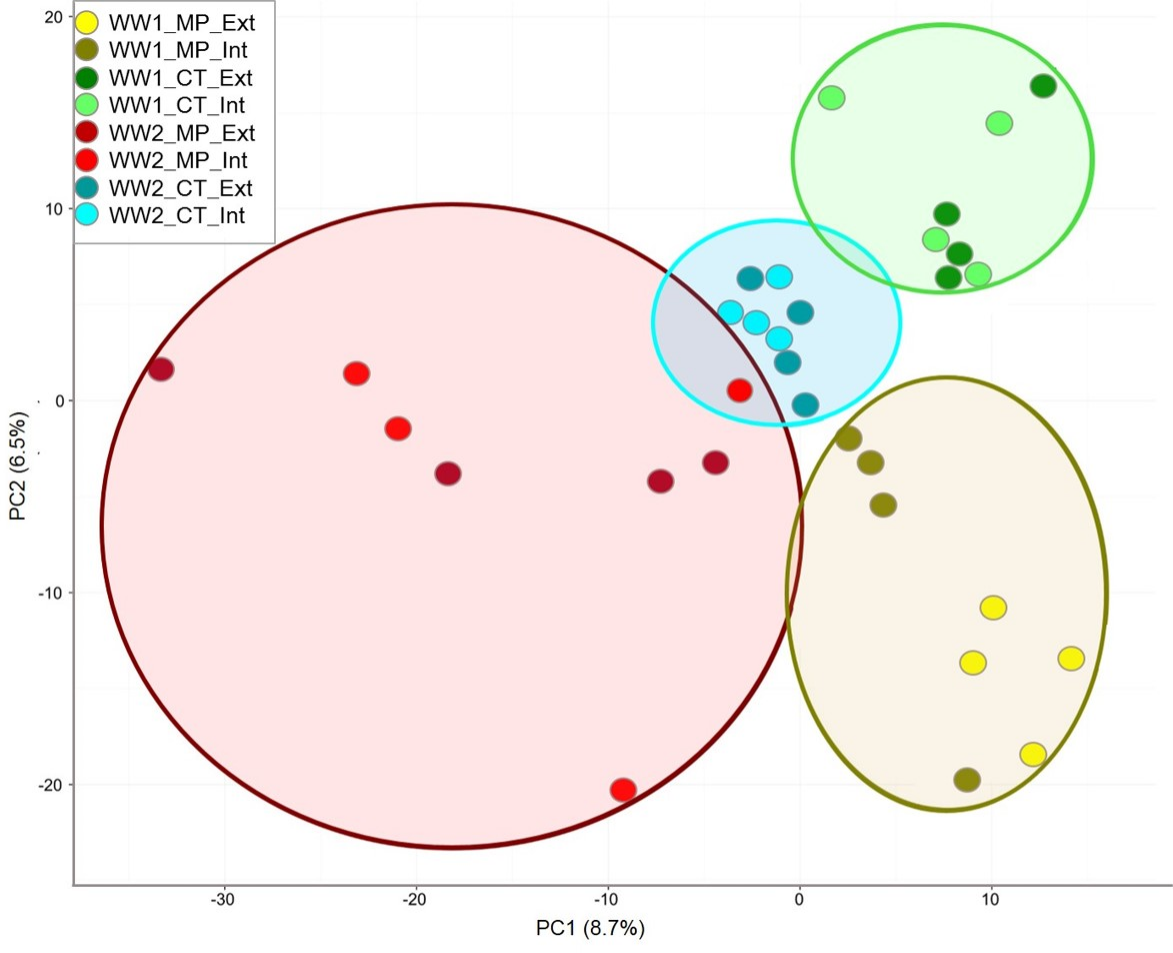
Two-dimensional principal component analysis (PCA) including all differentially abundant OTUs retrieved from the ITS1 dataset. Each colored dot represents one dataset originating from a distinct replicate of a specific soil treatment. Data points representing the same cluster are color-coded and framed by circles based on Ward’s minimum variance. For abbreviations refer to Fig 3.

A principal component analysis (PCA) was performed to describe the similarity within and variability between fungal soil communities as influenced by tillage practice, different pre-crops and N-fertilizer intensity. The first component explained 8.7% (ITS1) or 8.5% (ITS2), whereas the second component accounted for 6.5% (ITS1) or 7.5% (ITS2) of the total variance, respectively. Remarkably, distinct clusters for each treatment were found, showing high homogeneity among replicates. The greatest effects on fungal community structures were caused by soil tillage and pre-crop resulting in four distinct clusters (WW1_MP, WW1_CT, WW2_MP and WW2_CT). A greater variance was observed in the MP treatments, mainly due to variation within WW2. No differentiation of samples depending on fertilizer intensity was observable (Fig 7). Results were similar when based on ITS2 data with the exception that the greatest variance was found within the WW1 MP variant (S3 Fig). PCA analysis was confirmed by a PermANOVA analysis. Tillage and pre-crops had significant impact on the fungal community composition, while fertilization had the lowest and non-significant effect (Table 3).

**Table 3:** PermANOVA analysis of ITS1 and ITS2 data based on Bray-Curtis dissimilarities (10,000 permutations).

Factors	ITS1 Variance %	ITS2 Variance %
Tillage	28.93***	27.11***
Pre-crop	28.79***	26.87***
Fertilization	9.11	8.75
Pre-crop x Tillage	15.87**	13.68**

***p<0.001

**p<0.01

### Distinct effects of agricultural management on selected fungal genera

**Fig 8:**
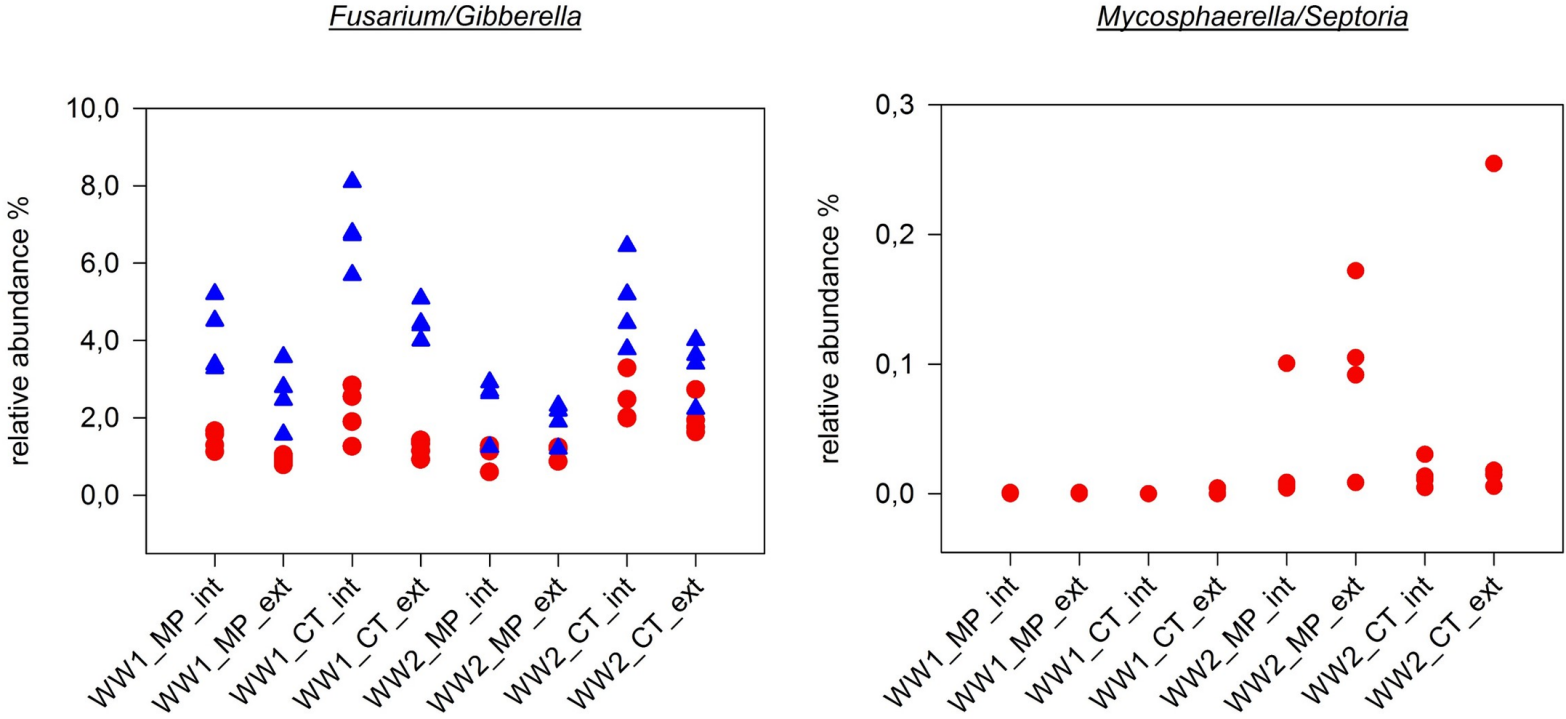
Fungal genera containing relevant phytopathogens combated with effective fungicides. *Fusarium*/*Gibberella* spp. were not effectively suppressed by fungicide application and accumulated in all intensive treatments, particularly in pre-crop maize samples (WW1). In contrast, *Mycospharella*/*Septoria* spp. were only enriched in pre-crop rapeseed samples (WW2) and more abundant in the MP extensive treatment that never received fungicides in the long-term field trial. *Fusarium*/*Gibberella* were detectable with the ITS1 (designated with a red dot) and ITS2 (designated with a blue triangle) primers, and *Mycosphaerella*/*Septoria* only with the ITS1 primers. For abbreviations refer to Fig 3.

To further detail the effects of soil management and pre-crop on specific groups of soil fungi, important genera including relevant phytopathogenic, mycoparasitic and plant beneficial species were selected from the ITS1 and ITS2 datasets. In the first instance, these were genera containing wheat pathogens. Concerning the pre-crop, only genera including rapeseed pathogens have been selected, since for pre-crop maize no relevant pathogen attacks have been reported in the past years of the long-term trial. In case of genera comprising numerous wheat pathogens, *Stagonospora* responded positively to the pre-crop maize and CT, and *Fusarium* was additionally enriched in intensive treatments (Table 4, Fig 8). These results were more pronounced in the ITS2 dataset. In the ITS1 data, *Fusarium* was significantly enriched in CT treatments of both pre-crops. *Gaeumannomyces* was only traceable within the ITS1 dataset and showed a significant positive response to intensive treatments and additionally within WW2 to MP. *Mycosphaerella* (Fig 8) mainly prevailed in the wheat field with the pre-crop rapeseed and was positively associated with plough and intensive fertilization (not significant). *Rhizoctonia* was significantly increased by extensive fertilization. With respect to genera including frequently observed rapeseed pathogens, all three primarily detected groups were consequently responding to the preceding crop rapeseed, in which *Phoma* was mainly present in the plough and *Verticillium* in the cultivator tillage variants. *Botryotinia* was enriched in the WW2 field plots, and in WW1 only found in the CT variants (Table 4).

**Table 4:** Selected putative phytopathogenic or plant beneficial fungal genera.

**Genera**	**ITS**	pre-crop	tillage	fertili-zation	pre-crop x tillage				pre-crop x fertilization				tillage x fertilization			
					within		within	within	within		within		within		within	within		within	within	within	within
					M	R	MP	CT	M	R	int	ext	MP	CT	int	ext
***Wheat pathogens***																
*Fusarium*	ITS1	-	-	-	CT	**CT**	-	R		int		-		-	R	-			
					p = 0.005	**p<0.001**		p = 0.008	p<0.001	p = 0.003									
	ITS2	M	**CT**	int	-				-				-			
		p<0.001	**p<0.001**	p<0.001												
*Gaeumanno-myces*^*1*^	ITS1^**†**^	-	-	int		-		**R**	-	-				-			
				p = 0.007	**p = 0.003**												
*Mycosphaerella*^*1*^	ITS1	**R**	-	-	-				-				-			
		**p<0.001**														
*Rhizoctonia*	ITS1	-	-	**ext**		-				-				-			
				**p<0.001**													
*Stagonospora*	ITS1	-	-	-	CT		-	-	**M**		-				-			
					p = 0.001	**p<0.001**												
	ITS2^**†**^	-	-	-	**CT**		-	-	**M**		-				-			
					**p<0.001**	**p<0.001**												
***Rapeseed pathogens***																
*Phoma*	ITS1	-	-	-	-	MP	**R**	-	-				-			
						p<0.001	**p<0.001**									
*Verticillium*	ITS1	**R**	CT		-	-				-				-			
		**p<0.001**	p = 0.001														
	ITS2	R	**CT**		-	-				-				-			
		p = 0.004	**p<0.001**														
*Botryotinia*^*1*^	ITS1	**R**	-	-	-				-				-			
		**p<0.001**														
***Plant beneficial fungi***																
*Trichoderma*	ITS1	**M**	-	-	-				-				-			
		**p<0.001**														
	ITS2	-	-	-	-				-				-			
*Coniothyrium*^*1*^	ITS1	R	-	**ext**		-				-				-			
		p = 0.014		**p = 0.006**													
***Yeast-like Heterobasidiomycetes*, *mycoparasites with Colacosomes***																
*Leucosporidiella*	ITS1	**R**	-	-	-				-				-			
		**p = 0.002**														
	ITS2	-	-	-	-	CT	-	**R**		-				-			
						p = 0.013		**p = 0.003**									
*Rhodotorula*	ITS1	R	**MP**		-	-				-				-			
		p<0.001	**p<0.001**														
	ITS2	-	**MP**		-	-				-				-			
			**p<0.001**														
***Glomeromycota*, *plant endosymbiotic fungi***																
*Ambispora*	ITS1	M	-	**int**		-				-				-			
		p = 0.004		**p<0.001**													
	ITS2	M	-	**int**		-				-				-			
		p = 0.001		**p<0.001**													
*Archaeospora*^*1*^	ITS1	**M**	CT		-	-				-				-			
		**p<0.001**	p = 0.004														
*Diversispora*	ITS1	**M**	-	-	-				-				-			
		**p<0.001**														
	ITS2	**M**	-	ext		-				-				-			
		**p<0.001**		p<0.001													
*Entrophospora*	ITS1	**M**	MP		-	-				-				-			
		**p<0.001**	p = 0.01														
	ITS2	-	-	-	-	**MP**	-	M		-				-			
						**p<0.001**		p = 0.008									
*Funneliformis*	ITS1	-	-	ext		MP		-	**M**	-	-				-			
				p<0.001	p = 0.002	**p<0.001**												
	ITS2	**M**	-	ext		-				-				-			
		**p<0.001**		p = 0.003													
*Glomus*	ITS1^**‡**^	M	-	-	-				-				**ext**	-	CT	-
		p<0.001											**p<0.001**		p = 0.002	
	ITS2^**‡**^	M	-	-	-				-				**ext**	-	CT	-
		p<0.001											**p<0.001**		p<0.001	
*Paraglomus*^*2*^	ITS2^**†**^	-	-	int		**MP**		-	M	-	-				-			
				p = 0.003	**p<0.001**	p<0.020												
*Rhizophagus*	ITS1^**†**^	**M**	-	-	-				-				-			
		**p<0.001**														
	ITS2	-	-	-	-				ext		-	-	**M**		-			
									p = 0.019	**p = 0.003**								
*Septoglomus*	ITS1	**M**	-	**-**	-				-				-			
		**p<0.001**														
	ITS2	**M**	-	**-**	-				-				-			
		**p = 0.041**														

***Genera***	**ITS**	**pre-crop x tillage x fertilization**								
		**within**	**within**	**within**	**within**	**within ext (averaging over levels of tillage)**				**within ext (averaging over both pre-crops)**
		**MP & int**	**CT & int**	**M & int**	**R & int**					
*Claroideoglomus*	ITS1	-	M	**CT**	-	M	-	-	-	-
			p<0.001	**p<0.001**		p<0.001				
	ITS2	-	M	**CT**	-	M		-	-	-	-
			p<0.001	**p<0.001**		p<0.001					

Potential phytopathogens have been selected according to long-term visible observations in the field trial. Additionally, putative plant beneficial fungi, prominent biocontrol agents, mycoparasites and plant endosymbiotic arbuscular mycorrhizal fungi (AMF, *Glomeromycota*) were selected (^1^only detectable in the ITS1 and ^2^only detectable in the ITS2 dataset). M (maize as pre-crop in winter wheat 1); R (rapeseed as pre-crop in winter wheat 2); MP–mould-board plough; CT–cultivator; int–intensive, ext–extensive N fertilization. Significance values describe enhanced fungal abundances. Significance levels are precise in case of response to only one factor; in case of more influencing factors the significance level of each comparison is given. Values in bold show the factor or the combination of factors with the highest difference between means and represent the highest effect per genus and ITS region.

† Normality test failed (p<0.050).

‡ Equal Variance test (Brown-Forsythe) failed (p<0.050). No significant differences were detected (-).

**Fig 9:**
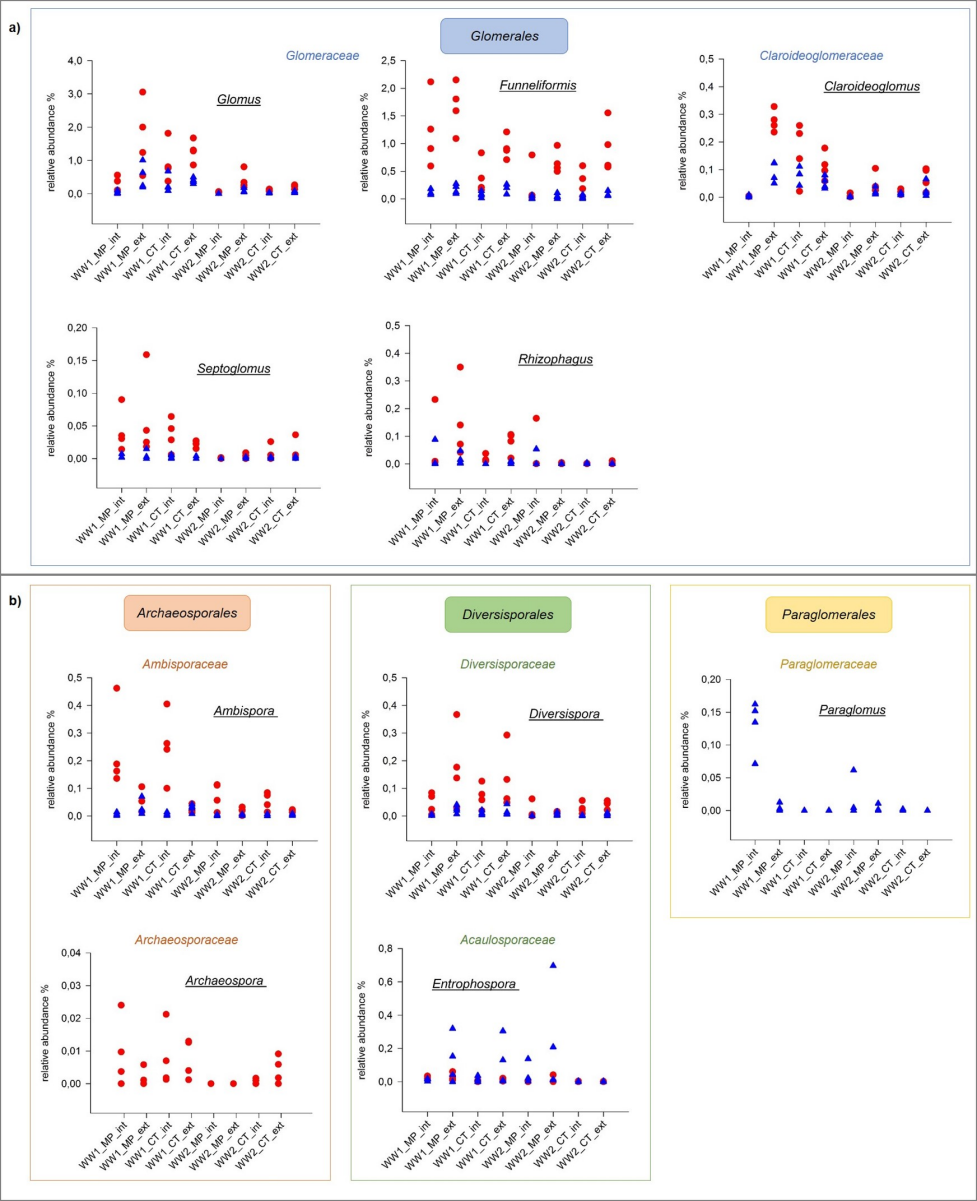
AMF genera detected in two winter wheat fields with different pre-crops. Each dot/triangle represents one DNA sample originating from the separately analyzed 4 replicates per soil treatment (WW1 pre-crop maize, WW2 pre-crop rapeseed). *Archaeospora* was only detectable with the ITS1 (designated with a red dot) and *Paraglomus* only with the ITS2 (designated with a blue triangle) primers. For abbreviations refer to Fig 3. Members of the order *Glomerales* are shown in the upper panel (a) and *Archaeosporales*, *Diversisporales* and *Paraglomerales* are shown in the lower panel (b).

Besides the *Glomeromycota* (Fig 9) four potentially plant beneficial fungal genera were detected and included prominent biocontrol agents. *Sebacina* did not exhibit a significant response (see also Section 3 of Discussion) and *Talaromyces* only featured a moderate correlation to agricultural management in the pre-crop rapeseed (p = 0.037) and was even more influenced by CT extensive treatments (p = 0.019). Regarding the other two putative plant beneficial genera (Table 4), the presence of the genus *Trichoderma* was strongly depending on the pre-crop maize. Although *Trichoderma* is an ubiquitously present, large genus (314 records in species fungorum) with high intra-species variability [34] uncertainties remain in resolving complex clades [35]; only few *Trichoderma* OTUs could be characterized (2 OTUs within ITS1 and 3 OTUs within ITS2 sequences). However, among this genus, several species with antagonistic potential and mycoparasitic life-style have been described, including well-known species like *T*. *harzianum*, *T*. *virens*, *T*. *asperellum* and many others [36]. *Coniothyrium* also represents a very large genus (663 records in species fungorum), but includes e.g. *Coniothyrium minitans*, a prominent and highly specific antagonist of *Sclerotinia sclerotiorum*, which causes i.a. stem-rot in rapeseed [37]. *Coniothyrium* was detected especially in the fields with the pre-crop rapeseed, but was, to a lower extent, also present in plots with the pre-crop maize (Table 4). Additionally, *Coniothyrium* showed significant positive association to extensive fertilization in both pre-crops.

Further mycoparasitic fungi detected in the dataset were members of the *Heterobasidiomycetes*. Five genera with yeast-like growth habit and the formation of colacosomes, also called lenticular bodies [38], appeared in the datasets. Colacosomes are regarded as strong indicators for mycoparasitism, since they are only developed during mycoparasitic interaction with prey fungi. From the identified genera, *Sporidiobolus* did not show a response to agricultural management, while *Platygloea* displayed positive correlation with the pre-crop maize, intensive fertilization and conservation tillage (p = 0.005). *Mastigobasidium* weakly responded within pre-crop maize to conservation tillage (p = 0.013 for ITS2). Two further genera (*Leucosporidiella* and *Rhodotorula*) displayed highly significant positive responses to the pre-crop rapeseed, with the first correlating to conservation tillage and the latter positively associated with MP (Table 4), in which *Rhodotorula* was by far the most prevalent genus.

Detailed analysis of endosymbiotic plant beneficial arbuscular mycorrhizal fungi (AMF) revealed strong responses to agricultural management (Table 4). The detected OTU richness was rather surprising, since 95 *Glomeromycota* OTUs have been detected within the ITS1 and 72 OTUs among the ITS2 sequence reads. OTUs per genus (ITS1/ITS2) were as follows: *Ambispora* (2/2), *Archaeospora* (2/0), *Claroideoglomus* (21/12), *Diversispora* (11/15), *Entrophospora* (3/1), *Funneliformis* (12/7), *Glomus* (36/25), *Paraglomus* (0/1), *Rhizophagus* (7/7) and *Septoglomus* (1/2). Besides the high variability, high number of OTUs, large number of obtained sequences (almost 63,000 sequence reads in total), distinct responses to agricultural management were verifiable. All genera were strongly enriched by pre-crop maize. Only the taxon *Entrophospora* was significantly correlated to rapeseed and MP tillage in the ITS2 dataset, whereas its correlation to the pre-crop maize was very high when considering the ITS1 data (Table 4). *Ambispora* and *Paraglomus* were the only AMF genera clearly correlated to intensive fertilization, whereas *Diversispora* and *Funneliformis* were associated to extensive N-donation. *Glomus* displayed differential prevalence with correlation to CT int and MP ext treatments. *Claroideoglomus* was the only genus, which showed significant interaction with all three factors at the intensive fertilization level and was mainly enriched in WW1-CT. Additionally, within the extensive fertilization plots, pre-crop maize had a positive effect on this genus independent of the applied soil management. MP *vs*. CT exerted the lowest single effects within the *Glomeromycota* and only *Archeospora* was directly correlated to conservation tillage. A combined impact of soil management and pre-crop maize was detected within the genera *Entrophospora* and *Paraglomus* in the ITS2 data and for *Funneliformis* within the ITS1 dataset. In this context, the latter two genera responded to pre-crop maize under MP (Table 4).

## Discussion

### Sampling, sample preparation and PCR strategy

Spatial variation in soil composition can have a significant impact on the detectable microbial diversity. To avoid bias, representative DNA of 1 g of soil was isolated [39] by using two different methods, and 15 individual PCRs at three different annealing temperatures were applied per sample to prevent stoichiometric effects [40]. Likewise, primer choice may also have a significant impact on the outcome of high-throughput amplicon sequencing studies. The constantly growing information in public databases, concerning fungal rDNA sequences, enables continued reassessment of primer design by *in-silico* analysis. One such study [15], based on roughly 135,000 fungal SSU rRNA gene sequences that contain the ITS1F priming site, revealed that the commonly utilized ITS1F primer covers less than 90% of the so far sequenced fungal taxa, even when one mismatch was allowed (*Ascomycota* 92.2%, *Basidiomycota* 80.5%, ‘non-dikarya’ 85.5%, total 89.8%). In contrast, the alternatively developed primer ITS1F-KYO2 [15] covers almost 99% of these taxa (*Ascomycota* 99.1%, *Basidiomycota* 99.2%, ‘non-dikarya’ 94.5%, total 98.8%). A subsequent study [16] also indicated profound mismatches with the commonly used ITS1F primer at the 3’-end among 44% of more than 4,000 tested sequences. Primer scores [41] were obtained for ITS1F (4.6) and the best score was found for ITS86F (0.5). The score for the primer ITS4 was rather poor (3.96, mismatches at the 3’-end in 16% of all cases). However, determination of amplification efficiencies was highest for the ITS86F/ITS4 primers (97.5%), which outperformed the other tested primer combinations targeting the ITS2 region. Additionally, the ITS86F primer featured mismatches at the 3’-end in only 3% of the sequences. The start of the exponential phase in qPCR analysis was determined at PCR cycle 20 [16]. In our study 21 cycles for the midpoint of the exponential phase were determined and set as the cut-off in our preparative ITS2 PCR experiments. We consider the upstream qPCR experiments as a very important measure prior to amplicon sequencing, since our results rarely revealed multiple primer occurrences [42], little nonsense sequences due to chimera formation or template switching [43], and no heavily overrepresented OTUs caused by over-amplification of individual PCR fragments in late PCR cycles [44].

### Differences in detected fungal diversity with ITS1 *vs*. ITS2 datasets

Detailed analysis of the two fungal ITS regions revealed a higher richness within the ITS1 dataset. However, diversity and evenness indices were higher in the ITS2 dataset, as was the number of detected fungal families and genera. Other metabarcoding studies also found ITS2 data from fungal communities being more diverse compared to ITS1 sequences. This was found e.g. for leaf-associated mycobiomes, when using the ITS1F and ITS4 primer pair in a pyrosequencing approach and analyzing the two ITS sequences separately [8]. Another study [45] on wood-decaying fungi achieved similar results. The primer pairs ITS1F and ITS2 as well as ITS3 and ITS4 were utilized to amplify the ITS1 and ITS2 regions, respectively. In that study ITS2 analysis yielded almost two times more OTUs as compared to the ITS1 analysis. This may be due to the different primers used for ITS1 compared to our study. Indeed, the primer pairs applied in our study had not been tested intensively in metabarcoding studies before. However, we also found higher diversity and evenness in the ITS2 dataset, but with the lack of ITS1 data the tremendous diversity among the *Zygo*- and *Glomeromycota* would have remained uncharacterized. Additionally, ITS1 information contributed two unique taxonomic orders and 76 unique genera, which were not detectable with the ITS2 primers. An argument for including ITS2 data in ecological studies addressing fungal communities is, besides higher diversity and evenness, the higher resolution to unravel influencing factors. In our ITS1 dataset, no significant correlation was found within six comparisons (Table 5). In contrast, we identified four significant correlations with the same number of comparisons analyzing the ITS2 data. The Simpson Index was highly significant for the factors pre-crop and tillage practice in both fields and the same was true for the Shannon Index. Fertilization practice did not show a significant impact on fungal community composition, neither with the ITS1 nor with the ITS2 data (Table 5). The little fertilization effect observed may be due to the nutrient-rich soil analyzed (Loess Chernozem).

**Table 5:** Influence of ITS1 and ITS2 data on significance levels of Simpson and Shannon diversity indices.

Factors	ITS1		ITS2	
	Simpson-Index	Shannon-Index	Simpson-Index	Shannon-Index
**Pre-crop**	n.s.	n.s	p = 0.011 *	p = 0.004 *
			WW1: 0.964	WW1: 4.174
			WW2: 0.940	WW2: 3.846
**Tillage**	n.s.	n.s.	p = 0.007 *	p = 0.011 *
			MP: 0.946	MP: 3.923
			CT: 0.967	CT: 4.183
**Fertilization**	n.s.	n.s.	n.s.	n.s.

*Significant differences according to Mann-Whitney-Rank Sum Test (p< 0.05)

n.s.–not significant; WW1 winter wheat–pre-crop maize; WW2 winter wheat–pre-crop rapeseed; MP–mould-board plough; CT–cultivator.

On the other hand, general considerations about ITS markers have to be included. High intra-species variation within ITS sequences has been reported for the *Glomeromycota* and a preferable marker gene (*RPB1*) has been suggested [3]. High intra-species variability most probably represents different genotypes in AMF species and the question remains, whether this variability is due to sexual reproduction or the multi-nucleate heterokaryotic nature of AMF mycelia [46]. An opposite situation has been reported for *Fusarium*/*Gibberella*. Many species cannot be resolved by using this marker type and the alternative marker gene *TEF1α* has been suggested [47]. In our study, *Fusarium*/*Gibberella* were represented by only three OTUs within the ITS1 and six OTUs within the ITS2 sequences. Such low diversity within the *Fusarium*/*Gibberella* community was not expected, especially not in the wheat field with maize as pre-crop, since this crop rotation is known to accumulate Fusariosis in both crops [48]. *Fusarium*/*Gibberella* accounted for up to 7% of all sequences in WW1 and all nine OTUs were detected, whereas in the wheat field with rapeseed as pre-crop, eight OTUs were found (5.25%). Thus, the high number of OTUs in the *Glomeromycota* would represent less species and the low number of OTUs in *Fusarium*/*Gibberella* would represent more species as suggested by the mere OTU counts.

### Fungal genera potentially relevant for plant health and yield in long-term farming practice

Taxonomic assignments from compiled ITS1 and ITS2 data were analyzed at the genus level. The following eight potentially phytopathogenic genera, namely *Alternaria*, *Bionectria*, *Epicoccum*, *Fusarium*, *Olpidium*, *Ophiosphaerella*, *Phoma* and *Verticillium* appeared in all soil samples at the 0.01% threshold. Hence, they belong to the core mycobiome of the investigated field site. Besides these eight genera common to all field sites, also potentially plant pathogenic fungal groups strongly affected by farming practice were detected. *Stagonospora* sp., including *S*. *nodorum*, the causal agent of *Stagonospora* blotch in wheat, was only present in the cultivator tillage variants of pre-crop maize, but therein highly abundant in the fields with intensive and extensive fertilization (Table 6). Although, studies on the development of infectious soilborne propagules and fungal plant diseases in response to farming practice are still rare, some reports exist for different cropping systems. Regarding a wheat monoculture and crop rotation (rapeseed) comparison, effects have been shown for *Fusarium* crown and root rots. Rapeseed in the crop rotation reduced these diseases by 10% (p<0.05), while the tillage effect was not significant [49]. In our analysis, *Fusarium*/*Gibberella* accumulated in all intensive treatments with reduced tillage and were less represented in pre-crop rapeseed samples. They appeared in the intensive variants despite the application of fungicides, while *Mycospharella*/*Septoria* were reduced by fungicide application (Fig 8). Additionally, the genus *Botryotinia*, which includes *B*. *cinerea* causing grey-mold in rapeseed, was above the threshold in pre-crop rapeseed and therein only abundant in the extensively fertilized CT variants. The genus *Rhizoctonia* that includes *R*. *solani* causing root and stem rot in rapeseed, showed an equal pattern of occurrence, while the genus *Thielaviopsis* (incl. *T*. *basicola*, causing black root rots in rapeseed) showed no such distinct response and appeared in WW1 and WW2 exclusively in the ploughed variants of intensive and extensive fertilization (Table 6). However, a comparable study in Northern Europe achieved different results. Here, lower incidence of *R*. *solani* and *T*. *basicola* was found in the variants with reduced tillage and fertilization [50], which may be due to different geo-climatic conditions.

**Table 6:** Putative phytopathogenic and plant beneficial fungal genera present in different soil treatments.

TREATMENTGroup	GENERA			
	common to all	common to all	unique, differential	unique, differential
PRE-CROP	WW1	WW2	WW1	WW2
pathogenic	#	#	*Monographella*	*Botryotinia*^*1*^
			*Rhizoctonia*^*1*^	*Mycosphaerella*^*1*^
			*Stagonospora*	*Pyrenophora*
			*Thielaviopsis*^*1*^	*Rhizoctonia*^*1*^
				*Thielaviopsis*^*1*^
				*Urocystis*^*1*^
beneficial	#	#	*Claroideoglomus*	*Coniothyrium*
	*Diversispora*		*Entrophospora*	*Claroideoglomus*
	*Septoglomus*		*Paraglomus*^*2*^	*Diversispora*
			*Rhizophagus*	*Entrophospora*
			*Talaromyces*^*2*^	*Talaromyces*^*2*^
**TILLAGE**	**CT**	**MP**	**CT**	**MP**
pathogenic	#	#	*Botryotinia*^*1*^	*Pyrenophora*
			*Monographella*	*Mycosphaerella*^*1*^
			*Mycosphaerella*^*1*^	*Thielaviopsis*^*1*^
			*Rhizoctonia*	
			*Stagonospora*	
			*Urocystis*^*1*^	
beneficial	#	#	*Entrophospora*	*Claroideoglomus*
	*Claroideoglomus*	*Entrophospora*	*Rhizophagus*	*Diversispora*
	*Diversispora*		*Septoglomus*	*Paraglomus*^*2*^
			*Talaromyces*^*2*^	*Rhizophagus*
				*Septoglomus*
**FERTILIZER**	**int**	**ext**	**int**	**ext**
pathogenic	#	#	*Mycosphaerella*^*1*^	*Botryotinia*^*1*^
			*Pyrenophora*	*Monographella*
			*Stagonospora*	*Mycosphaerella*^*1*^
			*Thielaviopsis*^*1*^	*Rhizoctonia*^*1*^
				*Stagonospora*
				*Thielaviopsis*^*1*^
				*Urocystis*^*1*^
beneficial	#	#	*Claroideoglomus*	*Coniothyrium*^*1*^
		*Claroideoglomus*	*Diversispora*	*Diversispora*
			*Entrophospora*	*Entrophospora*
			*Septoglomus*	*Rhizophagus*
				*Septoglomus*
				*Talaromyces*^*2*^

Combined ITS1 and ITS2 datasets were applied. Specific fungal genera had to be detected in at least two out of four replicates at an abundance >0.01%.

^1^only present in the ITS1 and

^2^only present in the ITS2 datasets. WW1/2 winter wheat pre-crops maize *vs*. rapeseed, CT/MP cultivator *vs*. plough tillage, int/ext intensive *vs*. extensive N-fertilization.

# represents the eight common genera mentioned in the text above.

From the visually classified fungal pathogens in the two wheat fields, only *Fusarium*/*Gibberella* (1–7%), *Phoma* (~2%) as well as *Verticillium* and *Alternaria* (both >1%) were highly prevalent. Fungicides were deployed against *Mycosphaerella*/*Septoria* and *Fusarium*/*Gibberella* (S1 Table). Intensive farming practice includes application of fungicides as well as high donations of N-fertilizer. Hence, the combined factors ‘intensive’ and ‘fungicides’ do not allow for separating their influences on yield and fungal communities (Fig 8).

Some potentially plant beneficial fungal genera (*Ambispora*, *Funneliformis*, *Glomus*, *Rhodotorula* and *Trichoderma*) were found to be common to all soil treatments and thus, did not severely respond to farming practice. However, other genera with known plant beneficial potential did show differential responses (Table 6). The investigation of plant beneficial fungi in agricultural soils–except AMF–is still in its infancy and limited to research on well-defined biocontrol agents like *Trichoderma* spp. or *Coniothyrium minitans* with designated efficacy against phytopathogens [36]. In our case, the genus *Trichoderma* was common to all field sites and *Coniothyrium* was only found in WW2 extensive treatments. Both are mycoparasitic, feeding on hyphae or sclerotia, respectively. Other mycoparasitic fungi (*Heterobasidiomycetes*) were in case of the genera *Rhodotorula* and *Platygloea* common to all field sites. The genus *Talaromyces* (*Ascomycota*), including *T*. *flavus*, a biocontrol agent against *Sclerotium rolfsii* and *Verticillium dahliae* [51], was not found in the ploughed and at the same time intensively fertilized soils. *Sebacina* spp. were present in both wheat fields, but were not detectable in WW1 CT int, WW2 MP ext and CT ext samples. A representative species for this group (*Piriformospora indica*) was discovered recently [52] and its plant beneficial potential was recognized contemporarily [53], which moved the complete order of the *Sebacinales* into the focus of intense research [54].

With regard to yield the intensive treatment was the most effective farming practice in the long term and independent from tillage practice or pre-crop (Fig 2). In the sampling year, slight differences appeared compared to the long-term average due to extreme weather conditions. Ploughing of soils tended to increase yield in pre-crop rapeseed, while higher wheat yield with pre-crop maize was depending on intensive fertilization combined with fungicides, regardless of the tillage practice. In contrast, tillage practice and pre-crop appeared to be the major drivers of fungal community composition, while the fertilization regime had no significant impact. Although higher yield is in many cases obtained with intensive farming practice, trade-offs between extensive land use and environmental impacts or ecosystem services that compensate yield losses should be considered when aiming at sustainable agriculture. Numerous reports exist about environmental benefits of extensive land use regarding e.g. filtering and availability of freshwater, increased biodiversity, prevention of soil erosion and degradation, avoidance of eutrophication, ecotoxicity and emission of greenhouse gases, savings in agro-chemicals, and less polluted food [55–56].

### AMF genera with distinct responses to tillage, fertilization or pre-crop

AMF communities have been frequently investigated in agro-ecosystems and in largely undisturbed environments. Reports addressed various crops [57], tillage practices [58] and fertilization regimes [59] for different farming soils; grasslands and forests are examples for largely undisturbed ecosystems [60–61]. Evidence was provided and the opinion prevailed that AMF preferably develop high diversity in soils with low pH, low available phosphorus and low organic matter content [62]. In contrast, the agricultural soil investigated in the present study has a neutral to alkaline pH with high amounts of phosphorus and organic matter (S3 Table). Additional P-underfoot fertilization was routinely applied for maize (100 kg di-ammonium hydrogen phosphate/ha). The other crops of the crop rotation did not receive additional P-fertilization. Despite the presumably unfavorable soil conditions for pronounced AMF development, unexpected high AMF diversity was detected, especially in pre-crop maize field plots. Taking all amplicon sequence reads into account, 1.235% belonged to the *Glomeromycota*, which may also be due to the new primer combinations tested. All genera displayed distinct responses to farming practice (Fig 9). Generally, AMF were by far more abundant in WW1 as compared to WW2 (except for *Glomus* and *Funneliformis*). This is in concordance with published results, were cultivation of *Brassica* sp. that are non-hosts for AMF reduced their prevalence [63]. This was often implicated to allelopathic, antifungal glucosinolates in root exudates of the *Brassicaceae*, although contradictory results on the efficacy of these allelochemicals on AMF communities have been reported [64–65].

The four detected AMF genera belonging to the *Glomeraceae* (Fig 9A) did not display homogeneous distribution. *Glomus* was more abundant in WW1 (Table 4) and in WW2 MP ext treatments. *Rhizophagus* showed a similar distribution pattern with striking differences concerning the MP variants of WW2, but with adverse appearance as compared to *Glomus*. *Funneliformis* was highly prevalent in all samples and highest in the MP WW1 plots. *Septoglomus* was mainly traceable in WW1 and therein slightly higher abundant in the MP variants, while it dominated in WW2 in the CT treatments. In face of the strong pre-crop effect, ploughing did not have a strong impact on the abundance of the *Glomeraceae* in general. However, *Glomus* and *Funneliformis* were significantly higher represented in MP samples. The assumption, that hyphal networks are disrupted by ploughing, which leads to hampered dispersal of the *Glomeraceae* [66] could not be verified at our field site in Central Germany. *Septoglomus* was the only member of the *Glomeraceae* that was negatively affected by ploughing and instead accumulated in the cultivator treatments. A comparable study in a maize field in Central Italy obtained similar results and found the *Glomeraceae* as main representatives of the *Glomeromycota* regardless of N-fertilization and tillage practice [67].

Besides the *Glomeraceae*, the *Glomerales* and *Paraglomerales* were represented with one family each (*Claroideoglomeraceae* and *Paraglomeraceae*, respectively) and for each family one genus was detected. *Claroideoglomus* was more abundant in the wheat field with maize as a pre-crop. Considering both pre-crops, it was clearly less abundant in the ploughed soils with intensive fertilization (Table 4). Herein, it was hardly detectable, while it was clearly present in all other soil treatments (Fig 9A). Taxonomic DNA profiling studies on *Claroideoglomus* are lacking, but it was frequently detected by microscopic methods and found among the most prevalent AMF genera in e.g. cowpea, maize and wheat fields [68]. *Paraglomus* (Fig 9B) was found in the present study to be highly abundant in the ploughed variants with intensive fertilization and herein strongly enriched in the wheat field with pre-crop maize (0.13%), but was completely absent in all cultivator tillage treatments with the exception of WW2 CT int, where it appeared at a very low rate (0.0013%). In contrast, other studies found a negative impact of intensive agricultural management on *Paraglomus* [69], or a positive impact of crop rotation and organic fertilization on the variability within this genus [70].

The *Diversisporales* were represented by two families (*Diversisporaceae*, *Acaulosporaceae*). The respective genera *Diversispora* and *Entrophospora* displayed a very similar pattern of distribution within WW1, where they were strongly enriched in the field plots with extensive fertilization with no influence of tillage practice (Fig 9B). In WW2, distribution patterns were scattered with *Diversispora* being slightly more abundant in the CT and *Entrophospora* with elevated levels in the MP tillage variants (Table 4). Previously it has been reported that the pre-crop had no significant influence on the prevalence of these genera, but both genera were preferably detected in the non-tillage variants [71]. Another study on a fertilization gradient in pasture grassland found a negative correlation between mineral N-fertilization and the number of *Diversispora* rRNA gene sequences [72]. This is in accordance with our results, where *Diversispora* together with *Funneliformis* were the only AM fungi that positively responded to reduced N-fertilization (Table 4). In contrast, the *Archaeosporales* (*Ambispora*, *Archaeospora*) in our study reacted adversely to fertilization as compared to the *Diversisporales*, and were positively correlated to intensive fertilization (Table 4, Fig 9B), independent of the pre-crop. Furthermore, *Archaeospora* was positively related to cultivator tillage. The latter genus was only detectable within the ITS1 dataset and represented by two OTUs. A more recent study reported similar results [73]. Among areas with three different fertilization intensities for restoration of local plant communities, they also found *Glomus* and *Funneliformis* being the most predominant genera. *Acaulospora*, *Ambispora*, *Entrophospora* and *Paraglomus* were observed in areas with high fertilization, whereas *Claroideoglomus*, *Diversispora* and *Septoglomus* were more abundant in areas with low fertilization. This is partly in line with the results presented here, where *Diversispora* and *Claroideoglomus* also positively responded to extensive and *Ambispora* as well as *Paraglomus* to intensive fertilization.

## Conclusions

The present study demonstrated alterations in fungal communities of agricultural soils in a long-term field trial due to different tillage and fertilization strategies in two distinct winter wheat fields with maize and rapeseed as preceding crops. Crop rotation is widely accepted to stabilize soil structure and fertility, and to positively affect pathogen and weed control. Furthermore—as described here–it exerts strong selective power on soil mycobiome structures. Although, highest wheat yield was in the long term obtained with conventional practice (ploughing and full N-fertilization, incl. fungicides), fungal genera including relevant pathogens were partly enriched in these field plots. Many potential plant beneficial fungal genera responded positively to pre-crop (e.g. AMF) and only marginally to soil management. The highest AMF diversity was detected in the wheat field with maize as preceding crop, but this had no positive influence on wheat yield, since this was generally higher with pre-crop rapeseed. These rather unexpected results indicate that agro-ecosystem functioning is not yet well understood. Simplistic prospects like high relative pathogen abundance leads to lower yield, or high AMF diversity leads to enhanced plant nutrition and thereby higher yield, fall short in such complex interactions.

The concept to implement both ITS regions for amplicon generation and high-throughput sequencing was justified by about 30% more detected fungal taxa compared to the results with only one ITS region included. Challenging for future improvement will be the achievement of higher taxonomic resolution by developing opportunities to link ITS1 and ITS2 sequence information. This would facilitate species identification, which is not possible with physically separated ITS1 and ITS2 sequence reads. Although the newly tested primer pairs performed well, primer biases or variable PCR efficiencies cannot be excluded. Additionally, limitations for validated reference sequences in databases and lack of information about functional and ecological roles of specific fungal taxa in agricultural soils impede complete comprehension. The challenge to present a survey about the inventory of fungal taxa in almost 25 years differently managed soils at the same farming site was designed as a baseline study focusing on reproducibility in the replicated field plots. Since this investigation embeds in a long-term research program (BonaRes) encouraging further studies until 2024, this work will be replicated to estimate the stability of soil mycobiomes over time. Investigating temporal fungal community dynamics throughout the growing season will be another future issue. The here basically ascertained crucial impact of long-term farming practice on soil mycobiota may be regarded as feasible contribution for further research. This could support attempts for soil biodiversity-based agro-ecosystem management to improve productivity of different soils with a wide variety of crops.

## Supporting information

S1 Table Applied fungicides in the long-term field trial. The overview considers the effectiveness against pathogens of crops, which were cultivated according to the crop rotation cycle.
(PDF)

S2 Table Cq values of upstream qPCR experiments. The numbers of PCR cycles for amplicon generation were determined for the ITS1 and ITS2 primers by qPCR.
(PDF)

S3 Table Physicochemical soil properties of the long-term field trial. Macro- and micronutrients as well as soil texture in the long-term field trial.
(PDF)

S4 Table Numbers of amplicon reads per replicate for ITS1 and ITS2 datasets. High quality reads obtained per replicate and primer pair including means and standard deviations.
(PDF)

S5 Table OTU abundances and taxonomic assignments per replicate based on ITS1 data. (XLS)

S6 Table OTU abundances and taxonomic assignments per replicate based on ITS2 data. (XLS)

S7 Table Shannon and Simpson diversity indices. Alpha diversity based on the ITS1 (a) and ITS2 datasets (b).
(PDF)

S1 Fig Rarefaction curves of OTUs for each soil treatment. Graphs are based on the mean of the four replicates including standard deviations.
(PDF)

S2 Fig Venn diagrams presenting fungal genera in differently managed soils. Graphs were focused on (a) MP *vs*. CT and (b) int *vs*. ext treatments.
(PDF)

S3 Fig Two-dimensional Principal Component Analysis (PCA) based on ITS1 and ITS2 data. Each colored dot represents one dataset originating from a distinct replicate of a specific soil treatment.
(PDF)

S1 File Kronaplot ITS1_WW1_MP_int_R3. (HTML)

S2 File Kronaplot ITS1_WW1_MP_ext_R1. (HTML)

S3 File Kronaplot ITS1_WW1_CT_int_R2. (HTML)

S4 File Kronaplot ITS1_WW1_CT_ext_R4. (HTML)

S5 File Kronaplot ITS1 WW2_MP_int_R4. (HTML)

S6 File Kronaplot ITS1 WW2_MP_ext_R1. (HTML)

S7 File Kronaplot ITS1_WW2_CT_int_R3. (HTML)

S8 File Kronaplot ITS1_WW2_CT_ext_R2. (HTML)

S9 File Kronaplot ITS2_WW1_MP_int_R4. (HTML)

S10 File Kronaplot ITS2_WW1_MP_ext_R1. (HTML)

S11 File Kronaplot ITS2_WW1_CT_int_R2. (HTML)

S12 File Kronaplot ITS2_WW1_CT_ext_R3. (HTML)

S13 File Kronaplot ITS2_WW2_MP_int_R1. (HTML)

S14 File Kronaplot ITS2_WW2_MP_ext_R4. (HTML)

S15 File Kronaplot ITS2 WW2_CT_int_R3. (HTML)

S16 File Kronaplot ITS2_WW2_CT_ext_R2. (HTML)
