# Supplementary material for: Fungal community profiles in agricultural soils of a long-term field trial under different tillage, fertilization and crop rotation conditions analyzed by high-throughput ITS-amplicon sequencing
Source: PLoS One. 2018 Apr 5;13(4):e0195345. doi: 10.1371/journal.pone.0195345 (PMC5886558; doi:10.1371/journal.pone.0195345)
Supplement: S1 Table — The overview considers the effectiveness against pathogens of crops, which were cultivated according to the crop rotation cycle. (PDF) [file pone.0195345.s001.pdf]

S1 Table. Applied fungicides in the long-term field trial.

Pathogen	Synonyms	Disease	Crop	Credo	Opus Top	Amistar Opti	Gladio
Drechslera tritici-vulgaris	*Pyrenophora tritici-repentis* *Helminthosporium tritici-repentis*	yellow leaf spot / tan spot	wheat	●	●	●	●
Erysiphe graminis	*Blumeria graminis, Oidium monilioides*	powdery mildew	wheat, barley	●[1]	●		●
Fusarium sp.	*Gibberella sp.*	Fusariosis	wheat				●
Puccinia recondita	*P. triticina*	brown rust	wheat	●	●	●	●
Puccinia striiformis	*P. glumarum, P. straminis*	stripe rust / yellow rust	wheat, barley	●[2]	●		●[2]
Septoria nodorum	*Parastagonospora nodorum, Stagonospora nodorum Phaeosphaeria nodorum Leptosphaeria nodorum*	*Stagonospora* blotch	wheat		●		●
Septoria tritici	*Mycosphaerella graminicola*	*Septoria* leaf blotch	wheat	●	●	●	●

Fungicides were applied (●) jointly during the sampling year only in the intensive winter wheat variants on BBCH 31 (Opus Top und Credo) and BBCH 49 (Amistar Opti and Gladio), respectively. The overview considers only the effectiveness against pathogens of crops, which were cultivated in the field according to the crop rotation cycle ‘maize, winter wheat (WW1), barley, rapeseed, winter wheat (WW2)’, [1]effective on barley, [2]effective on wheat.
