# Supplementary material for: Fungal community profiles in agricultural soils of a long-term field trial under different tillage, fertilization and crop rotation conditions analyzed by high-throughput ITS-amplicon sequencing
Source: PLoS One. 2018 Apr 5;13(4):e0195345. doi: 10.1371/journal.pone.0195345 (PMC5886558; doi:10.1371/journal.pone.0195345)
Supplement: S2 Table — The numbers of PCR cycles for amplicon generation were determined for the ITS1 and ITS2 primers by qPCR. (PDF) [file pone.0195345.s002.pdf]

S2 Table. Cq values of upstream qPCR experiments. The numbers of PCR cycles for amplicon generation were determined for the ITS1 and ITS2 primers by qPCR.

**Samples**	**C_q of ITS1**	**C_q of ITS2**
WW1_MP_int_R3 1st isolation	20.06	18.55
WW1_MP_int_R2 3rd isolation	18.85	17.95
WW1_MP_int_R4 3rd isolation	19.17	17.93
WW1_CT_int_R1 1st isolation	20.13	18.67
WW1_CT_int_R1 2nd isolation	18.03	18.29
WW1_MP_ext_R1 3rd isolation	19.51	17.74
WW1_CT_int_R1 3rd isolation	20.26	18.60
WW1_MP_ext_R2 1st isolation	19.51	18.05
WW1_CT_int_R4 2nd isolation	18.19	18.13
