# Supplementary material for: Fungal community profiles in agricultural soils of a long-term field trial under different tillage, fertilization and crop rotation conditions analyzed by high-throughput ITS-amplicon sequencing
Source: PLoS One. 2018 Apr 5;13(4):e0195345. doi: 10.1371/journal.pone.0195345 (PMC5886558; doi:10.1371/journal.pone.0195345)
Supplement: S3 Table — Macro- and micronutrients as well as soil texture in the long-term field trial. (PDF) [file pone.0195345.s003.pdf]

S3 Table. Physicochemical soil properties of the long-term field trial. Macro- and micronutrients as well as soil texture in the long-term field trial.

pH	P2O5	K	Mg	N	S	C total.	C org.	C anorg.
CaCl2	CAL	CAL	CaCl2	EA	EA	EA	EA	EA
	mg/100g	mg/100g	mg/100g	%	%	%	%	%
7.4	15	26	11	0.202	0.042	2.95	2.36	0.590

Cu	Fe	Mg	Mn	Zn	K
CAT	CAT	CAT	CAT	CAT	CAT
mg/kg	mg/kg	mg/kg	mg/kg	mg/kg	mg/kg
4.28	20.0	127	63.6	28.9	137

B	Ca	Cu	Fe	K	Mg	Mn	P	Zn
ICP-OES	ICP-OES	ICP-OES	ICP-OES	ICP-OES	ICP-OES	ICP-OES	ICP-OES	ICP-OES
mg/kg	mg/kg	mg/kg	mg/kg	mg/kg	mg/kg	mg/kg	mg/kg	mg/kg
16.7	22,863	26.7	15,337	2,887	4,046	483	459	180

				KGA-3	KGA-3	KGA-3
Carbonate	NH4-N	NO3-N	P Olsen	Sand	Silt	Clay
%	mg/kg	mg/kg	mg/kg	%	%	%
5.89	6.20	68.0	21.9	8	70	22
