# Supplementary material for: Fungal community profiles in agricultural soils of a long-term field trial under different tillage, fertilization and crop rotation conditions analyzed by high-throughput ITS-amplicon sequencing
Source: PLoS One. 2018 Apr 5;13(4):e0195345. doi: 10.1371/journal.pone.0195345 (PMC5886558; doi:10.1371/journal.pone.0195345)
Supplement: S4 Table — High quality reads obtained per replicate and primer pair including means and standard deviations. (PDF) [file pone.0195345.s004.pdf]

S4 Table. Numbers of amplicon reads per replicate for ITS1 and ITS2 datasets. High quality reads obtained per replicate and primer pair including means and standard deviations.

Samples	ITS1	Means ± SD	ITS2	Means ± SD
WW1_MP_int_R1	100,169	91,906 ± 13,673	69,297	69,882 ± 18,924
WW1_MP_int_R2	106,710		60,658	
WW1_MP_int_R3	82,298		96,475	
WW1_MP_int_R4	78,448		53,098	
WW1_MP_ext_R1	89,580	101,498 ± 13,635	80,089	69,359 ± 9,915
WW1_MP_ext_R2	104,576		56,144	
WW1_MP_ext_R3	119,467		69,579	
WW1_MP_ext_R4	92,368		71,623	
WW1_CT_int_R1	99,268	89,919 ± 15,731	51,929	63,776 ± 12,096
WW1_CT_int_R2	79,832		78,723	
WW1_CT_int_R3	106,916		56,296	
WW1_CT_int_R4	73,659		68,157	
WW1_CT_ext_R1	76,829	91,731 ± 15,611	51,068	59,214 ± 12,653
WW1_CT_ext_R2	81,578		57,427	
WW1_CT_ext_R3	111,044		50,737	
WW1_CT_ext_R4	97,474		77,623	
WW2_MP_int_R1	91,243	88,818 ± 12,413	48,193	69,983 ± 19,632
WW2_MP_int_R2	105,105		95,545	
WW2_MP_int_R3	82,295		71,330	
WW2_MP_int_R4	76,629		64,863	
WW2_MP_ext_R1	72,141	76,589 ± 4,178	76,202	84,510 ± 11,509
WW2_MP_ext_R2	81,424		95,436	
WW2_MP_ext_R3	74,259		73,063	
WW2_MP_ext_R4	78,530		93,340	
WW2_CT_int_R1	74,005	79,865 ± 19,443	59,849	72,614 ± 16,286
WW2_CT_int_R2	70,236		60,149	
WW2_CT_int_R3	66,549		94,148	
WW2_CT_int_R4	108,669		76,311	
WW2_CT_ext_R1	91,655	90,153 ± 25,524	80,987	72,094 ± 19,050
WW2_CT_ext_R2	106,374		60,537	
WW2_CT_ext_R3	108,969		94,312	
WW2_CT_ext_R4	53,615		52,538	
