# Supplementary material for: Fungal community profiles in agricultural soils of a long-term field trial under different tillage, fertilization and crop rotation conditions analyzed by high-throughput ITS-amplicon sequencing
Source: PLoS One. 2018 Apr 5;13(4):e0195345. doi: 10.1371/journal.pone.0195345 (PMC5886558; doi:10.1371/journal.pone.0195345)
Supplement: S7 Table — Alpha diversity based on the ITS1 (a) and ITS2 datasets (b). (PDF) [file pone.0195345.s007.pdf]

S7 Table. Shannon and Simpson diversity indices. Alpha diversity based on the ITS1 **(a)** and ITS2 dataset **(b)**.

a) Shannon and Simpson Indices based on ITS1 dataset

Samples	Shannon index	Means ± SD	Simpson index	Means ± SD
WW1_MP_int_R1	4.09	**3.94 ± 0.15**	0,960	**0.954 ± 0.009**
WW1_MP_int_R2	3.97		0,956	
WW1_MP_int_R3	3.74		0,941	
WW1_MP_int_R4	3.98		0,960	
WW1_MP_ext_R1	4.18	**3.99 ± 0.27**	0,960	**0.952 ± 0.017**
WW1_MP_ext_R2	3.92		0,951	
WW1_MP_ext_R3	4.22		0,967	
WW1_MP_ext_R4	3.65		0,928	
WW1_CT_int_R1	4.21	**4.04 ± 0.17**	0,959	**0.953 ± 0.007**
WW1_CT_int_R2	4.03		0,953	
WW1_CT_int_R3	3.81		0,944	
WW1_CT_int_R4	4.09		0,957	
WW1_CT_ext_R1	4.03	**3.77 ± 0.26**	0,954	**0.923 ± 0.036**
WW1_CT_ext_R2	3.92		0,942	
WW1_CT_ext_R3	3.71		0,923	
WW1_CT_ext_R4	3.43		0,873	
WW2_MP_int_R1	3.95	**3.72 ± 0.39**	0,952	**0.929 ± 0.041**
WW2_MP_int_R2	3.14		0,869	
WW2_MP_int_R3	3.98		0,957	
WW2_MP_int_R4	3.80		0,938	
WW2_MP_ext_R1	3.94	**3.90 ± 0.30**	0,954	**0.945 ± 0.020**
WW2_MP_ext_R2	3.47		0,914	
WW2_MP_ext_R3	4.03		0,952	
WW2_MP_ext_R4	4.15		0,958	
WW2_CT_int_R1	3.75	**3.61 ± 0.13**	0,930	**0.921 ± 0.008**
WW2_CT_int_R2	3.53		0,918	
WW2_CT_int_R3	3.47		0,912	
WW2_CT_int_R4	3.67		0,925	
WW2_CT_ext_R1	3.88	**3.83 ± 0.12**	0,950	**0.945 ± 0.009**
WW2_CT_ext_R2	3.94		0,950	
WW2_CT_ext_R3	3.65		0,931	
WW2_CT_ext_R4	3.86		0,949	

Soil samples were investigated from mould-board plough intensive (MP_int), mould-board plough extensive (MP_ext), conservation tillage intensive (CT_int) and CT extensive variants (CT_ext) in four replicates. Values are presented for each of the four replicates (R1-R4) and as means ± standard deviation. Alpha diversity values based on ITS1 dataset (a) and ITS2 dataset (b).

b) Shannon and Simpson Indices based on ITS2 dataset

Samples	Shannon index	Means ± SD	Simpson index	Means ± SD
WW1_MP_int_R1	4.17	**3.98 ± 0.16**	0.960	**0.947 ± 0.016**
WW1_MP_int_R2	3.80		0.925	
WW1_MP_int_R3	4.03		0.956	
WW1_MP_int_R4	3.91		0.948	
WW1_MP_ext_R1	4.43	**3.91 ± 0.64**	0.974	**0.930 ± 0.066**
WW1_MP_ext_R2	4.18		0.967	
WW1_MP_ext_R3	4.07		0.947	
WW1_MP_ext_R4	2.98		0.832	
WW1_CT_int_R1	4.36	**4.22 ± 0.10**	0.973	**0.965 ± 0.009**
WW1_CT_int_R2	4.23		0.971	
WW1_CT_int_R3	4.16		0.959	
WW1_CT_int_R4	4.12		0.956	
WW1_CT_ext_R1	4.33	**4.38 ± 0.10**	0.975	**0.975 ± 0.005**
WW1_CT_ext_R2	4.29		0.970	
WW1_CT_ext_R3	4.40		0.975	
WW1_CT_ext_R4	4.51		0.981	
WW2_MP_int_R1	3.93	**3.57 ± 0.49**	0.952	**0.901 ± 0.069**
WW2_MP_int_R2	2.84		0.799	
WW2_MP_int_R3	3.75		0.931	
WW2_MP_int_R4	3.76		0.923	
WW2_MP_ext_R1	3.62	**3.61 ± 0.52**	0.926	**0.910 ± 0.063**
WW2_MP_ext_R2	2.87		0.817	
WW2_MP_ext_R3	4.00		0.946	
WW2_MP_ext_R4	3.95		0.950	
WW2_CT_int_R1	3.69	**4.04 ± 0.26**	0.912	**0.951 ± 0.027**
WW2_CT_int_R2	4.01		0.957	
WW2_CT_int_R3	4.18		0.964	
WW2_CT_int_R4	4.28		0.973	
WW2_CT_ext_R1	3.68	**3.71 ± 0.61**	0.934	**0.907 ± 0.099**
WW2_CT_ext_R2	4.12		0.965	
WW2_CT_ext_R3	4.19		0.968	
WW2_CT_ext_R4	2.87		0.759	
