# Supplementary material for: Fungal community profiles in agricultural soils of a long-term field trial under different tillage, fertilization and crop rotation conditions analyzed by high-throughput ITS-amplicon sequencing
Source: PLoS One. 2018 Apr 5;13(4):e0195345. doi: 10.1371/journal.pone.0195345 (PMC5886558; doi:10.1371/journal.pone.0195345)

S1 Fig. Rarefaction curves of OTUs for each soil treatment. Graphs are based on the mean of the four replicates including standard deviations.

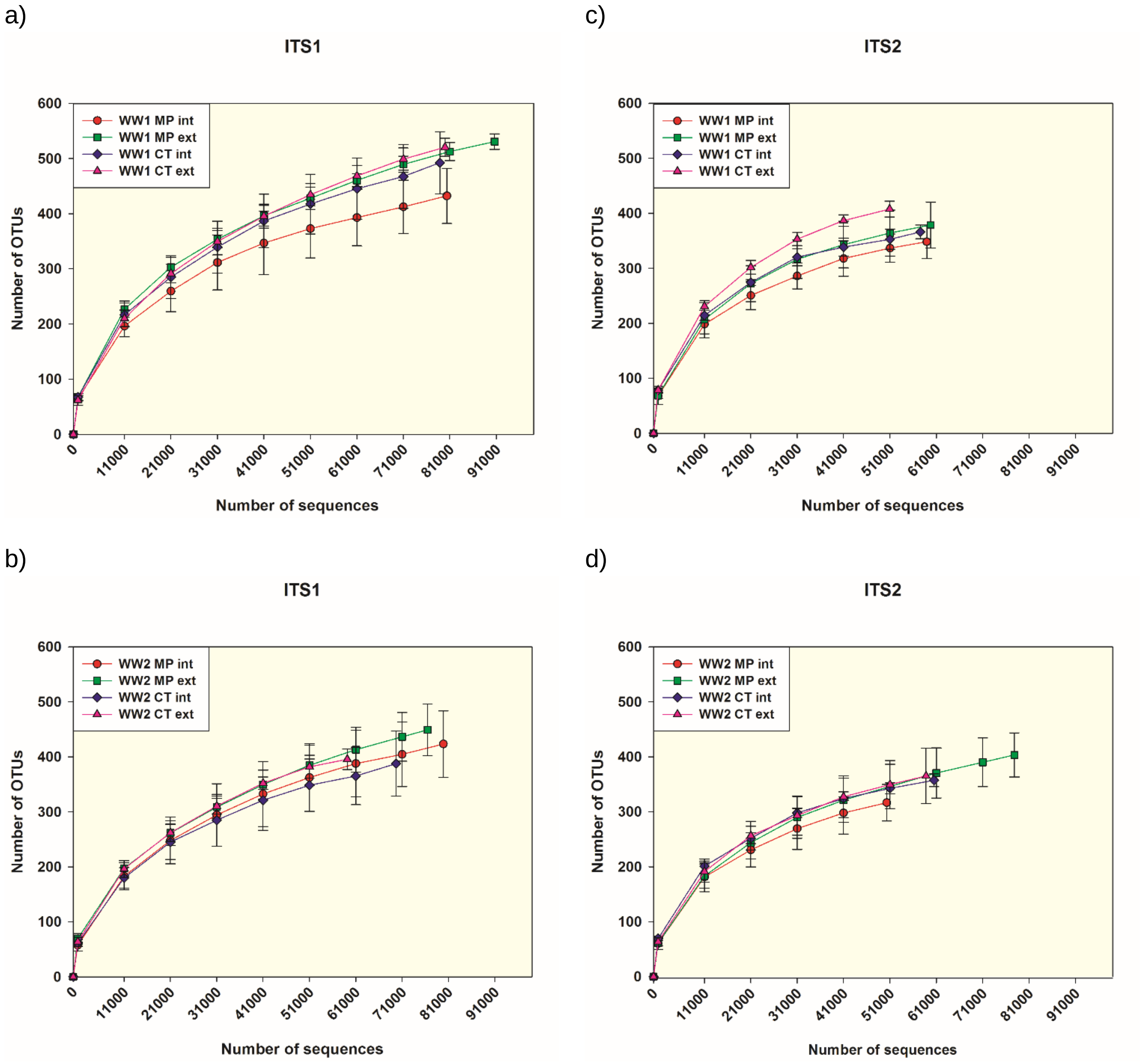

Supplement: S1 Fig — Graphs are based on the mean of the four replicates including standard deviations. (PDF) [file pone.0195345.s008.pdf]
