# Supplementary material for: Fungal community profiles in agricultural soils of a long-term field trial under different tillage, fertilization and crop rotation conditions analyzed by high-throughput ITS-amplicon sequencing
Source: PLoS One. 2018 Apr 5;13(4):e0195345. doi: 10.1371/journal.pone.0195345 (PMC5886558; doi:10.1371/journal.pone.0195345)
Supplement: S2 Fig — Graphs were focused on (a) MP vs. CT and (b) int vs. ext treatments. (PDF) [file pone.0195345.s009.pdf]

S2 Fig. Venn Diagrams presenting fungal genera in differently managed soils. Graphs were focused on **(a)** MP *vs*. CT and **(b)** int *vs*. ext treatments.

a)

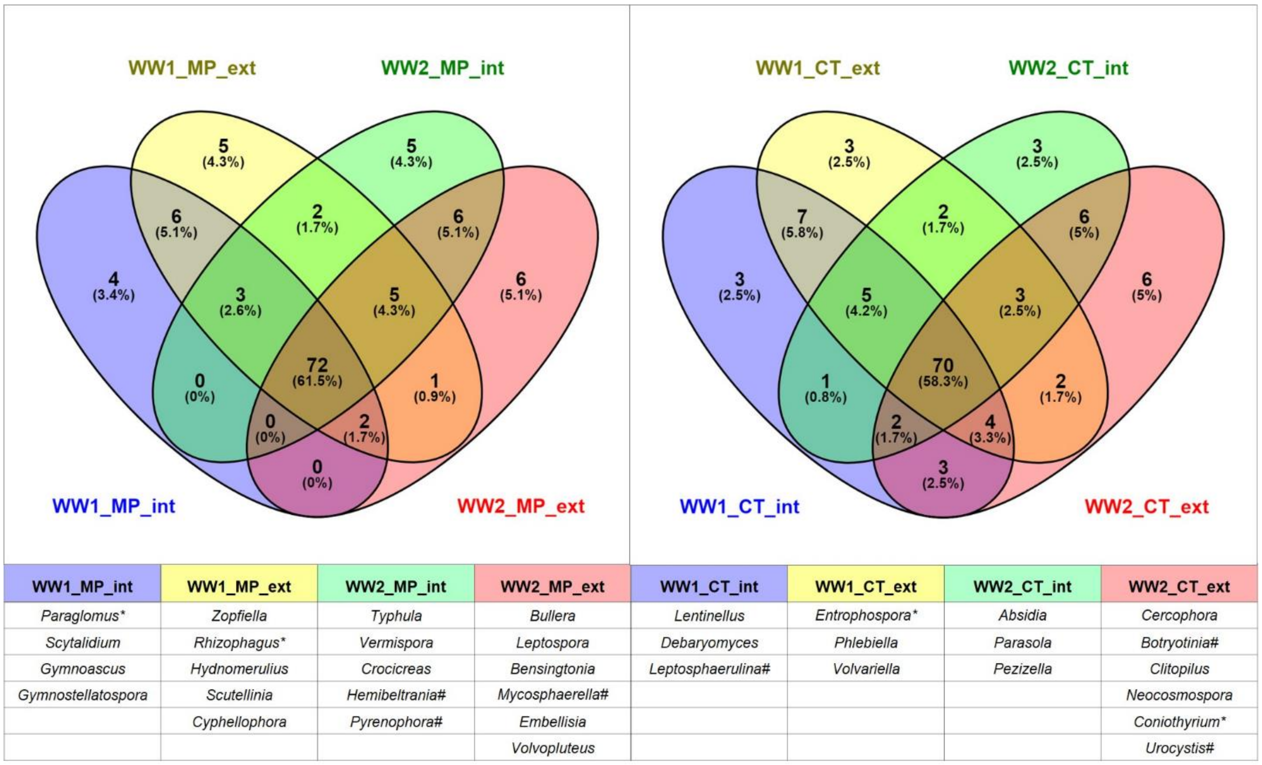

WW1_MP_int	WW1_MP_ext	WW2_MP_int	WW2_MP_ext	WW1_CT_int	WW1_CT_ext	WW2_CT_int	WW2_CT_ext
*Paraglomus**	*Zopfiella*	*Typhula*	*Bullera*	*Lentinellus*	*Entrophospora**	*Absidia*	*Cercophora*
Scytalidium	*Rhizophagus**	*Vermispora*	*Leptospora*	*Debaryomyces*	*Phlebiella*	*Parasola*	*Botryotinia#*
Gymnoascus	*Hydnomerulius*	*Crocicreas*	*Bensingtonia*	*Leptosphaerulina#*	*Volvariella*	*Pezizella*	*Clitopilus*
Gymnostellatospora	*Scutellinia*	*Hemibeltrania#*	*Mycosphaerella#*				*Neocosmospora*
	Cyphellophora	*Pyrenophora#*	*Embellisia*				*Coniothyrium**
			Volvopluteus				*Urocystis#*

b)

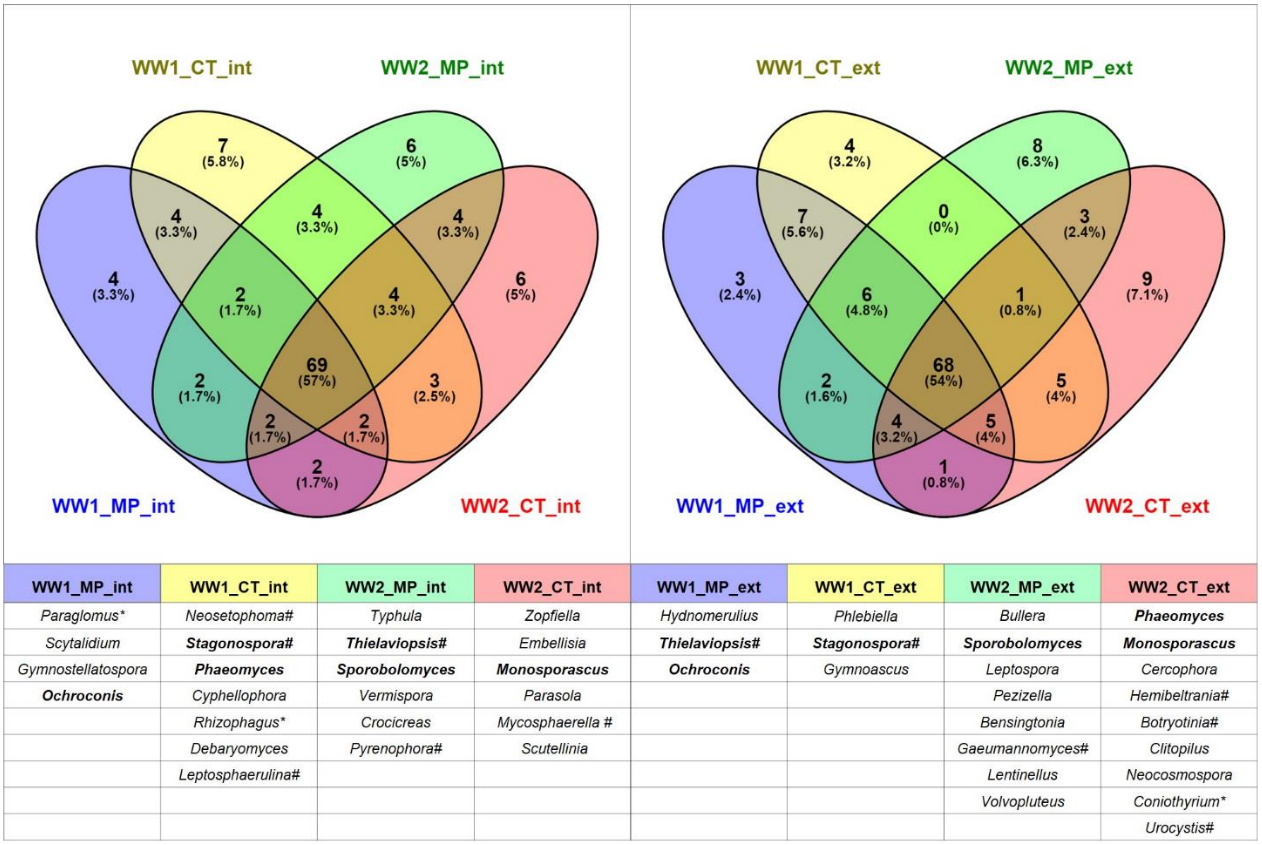

WW1_MP_int	WW1_CT_int	WW2_MP_int	WW2_CT_int	WW1_MP_ext	WW1_CT_ext	WW2_MP_ext	WW2_CT_ext
Paraglomus*	Neosetophoma#	Typhula	Zopfiella	Hydnomerulius	Phlebiella	Bullera	**Phaeomyces**
Scytalidium	**Stagonospora#**	**Thielaviopsis#**	Embellisia	**Thielaviopsis#**	**Stagonospora#**	**Sporobolomyces**	**Monosporascus**
Gymnostellatospora	**Phaeomyces**	**Sporobolomyces**	**Monosporascus**	**Ochroconis**	Gymnoascus	Leptospora	Cercophora
**Ochroconis**	Cyphellophora	Vermispora	Parasola			Pezizella	Hemibeltrania#
	Rhizophagus*	Crocicreas	Mycosphaerella #			Bensingtonia	Botryotinia#
	Debaryomyces	Pyrenophora#	Scutellinia			Gaeumannomyces#	Clitopilus
	Leptosphaerulina#					Lentinellus	Neocosmospora
						Volvopluteus	Coniothyrium*
							Urocystis#
