# Supplementary material for: Fungal community profiles in agricultural soils of a long-term field trial under different tillage, fertilization and crop rotation conditions analyzed by high-throughput ITS-amplicon sequencing
Source: PLoS One. 2018 Apr 5;13(4):e0195345. doi: 10.1371/journal.pone.0195345 (PMC5886558; doi:10.1371/journal.pone.0195345)

S3 Fig. Two-dimensional Principal Component Analysis (PCA) based on ITS1 and ITS2 data. Each colored dot represents one dataset originating from a distinct replicate of a specific soil treatment.

a) Original output based on ITS1 dataset

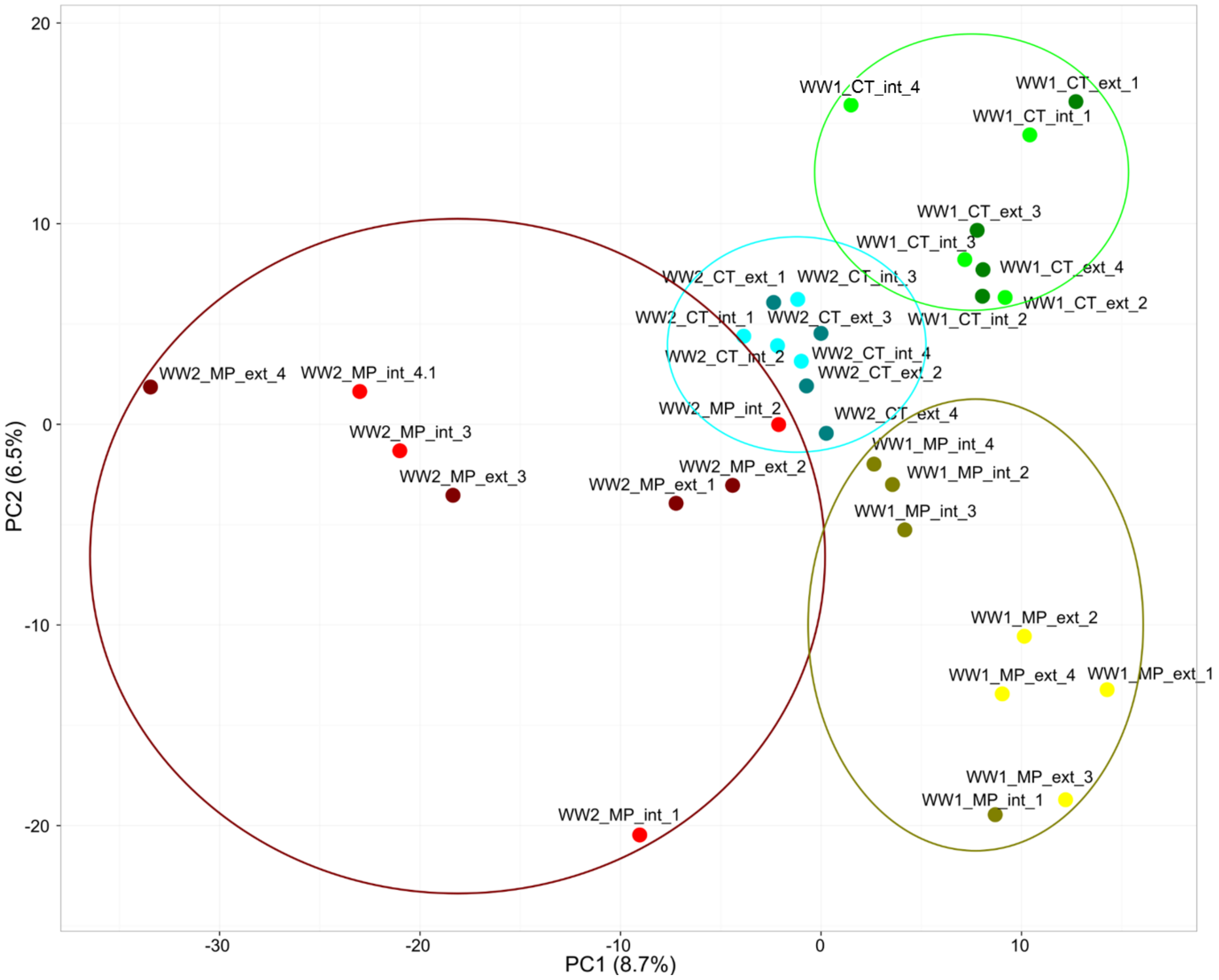

b) Original output based on ITS2 dataset

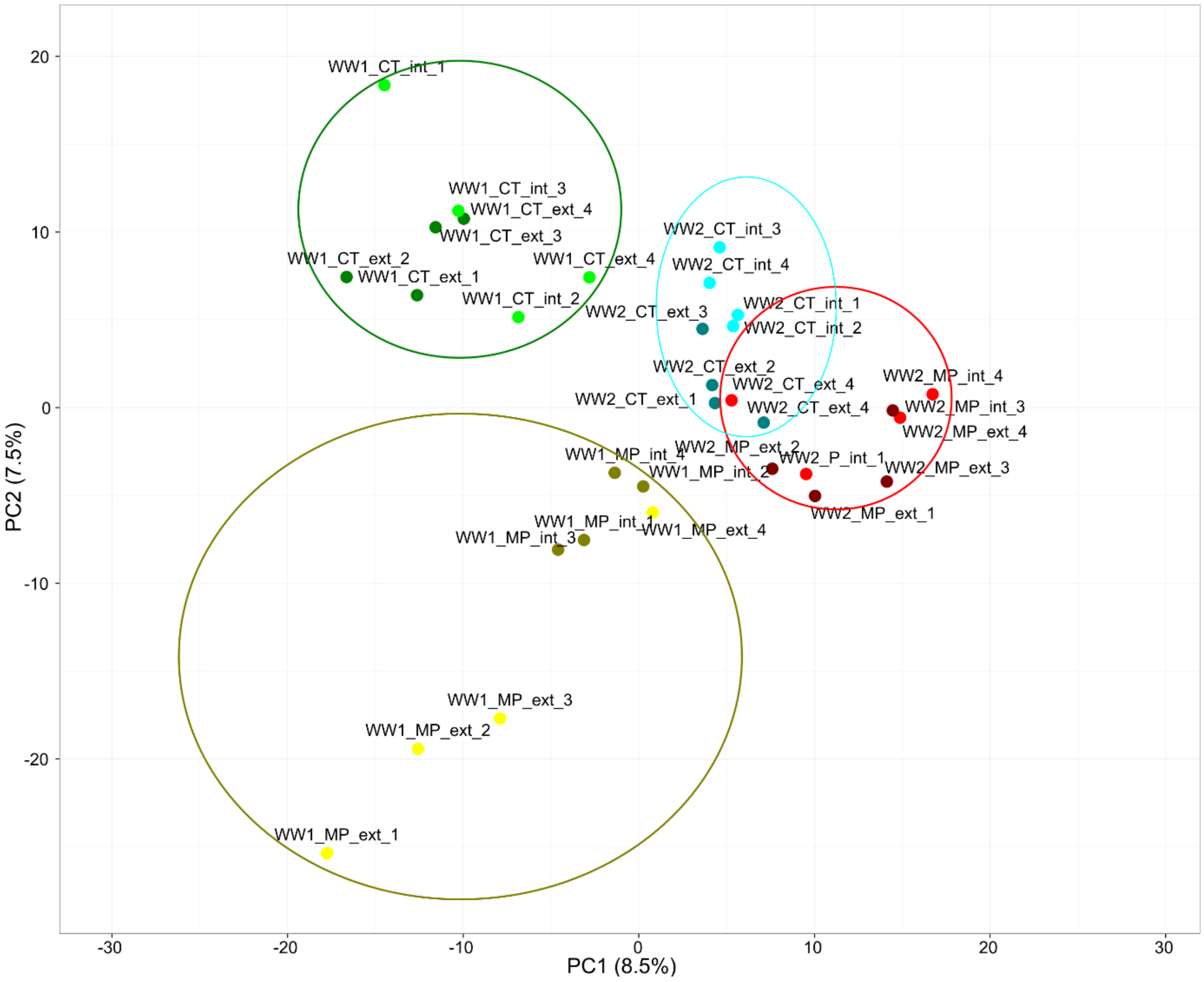

Supplement: S3 Fig — Each colored dot represents one dataset originating from a distinct replicate of a specific soil treatment. (PDF) [file pone.0195345.s010.pdf]
