# Supplementary material for: Fungal community profiles in agricultural soils of a long-term field trial under different tillage, fertilization and crop rotation conditions analyzed by high-throughput ITS-amplicon sequencing
Source: PLoS One. 2018 Apr 5;13(4):e0195345. doi: 10.1371/journal.pone.0195345 (PMC5886558; doi:10.1371/journal.pone.0195345)
Supplement: S1 File — (HTML) [file pone.0195345.s011.html]

Javascript must be enabled to view this page.

members
count
unassigned
score
rank

ITS1BC7.fastq\_final.fastq\_classified\_otusc\_clean


82298

82298
domain
100

phylum
99.3034
1490

class
80
27

80
order
27

family
80
27

genus
80
27
node6.members.0.js

class
96
29

96
order
29

29
family
96

node10.members.0.js
29
96
genus

class
99.2852
1434

163
order
94.8896

8
88
family

88
genus
node14.members.0.js
8

family
95.2452
155

genus
95.2452
155
node16.members.0.js

99.7031
order
1253

99.7031
family
1253

node19.members.0.js
3
genus
80

746
node20.members.0.js
genus
99.3834

genus
88.1429
7
node21.members.0.js

genus
99.6761
497
node22.members.0.js

18
100
order

family
100
3

node25.members.0.js
3
genus
100

family
100
15

100
genus
node27.members.0.js
15

99.3686
phylum
37344

100
class
2

2
order
100

2
100
family

genus
100
2
node32.members.0.js

96.1771
class
4004

911
85.3074
order

family
85.3074
911

node36.members.0.js
911
85.3074
genus

99.2373
order
3093

155
97.9097
family

genus
100
5
node39.members.0.js

100
genus
42
node40.members.0.js

genus
80
node41.members.0.js
108

family
99.686
2838

99.686
genus
node43.members.0.js
2838

80
family
100

80
genus
100
node45.members.0.js

6
100
class

6
100
order

6
100
family

100
genus
6
node49.members.0.js

3311
80
class

order
80
3311

family
80
3311

genus
80
node53.members.0.js
3311

99
class
388

388
99
order

388
93.0696
family

genus
93.0696
388
node57.members.0.js

98.7208
class
11366

order
100
3

family
100
3

node61.members.0.js
3
100
genus

16
order
100

91
family
16

16
node64.members.0.js
genus
91

5881
order
99.6944

family
99.8718
1123

genus
89.7143
node67.members.0.js
14

node68.members.0.js
1101
genus
96

genus
100
node69.members.0.js
8

524
100
family

99.9786
genus
node71.members.0.js
468

56
node72.members.0.js
genus
99.9286

2
family
80

node74.members.0.js
2
genus
80

7
family
100

100
genus
node76.members.0.js
7

27
family
88.5185

88.5185
genus
node78.members.0.js
27

5
100
family

100
genus
5
node80.members.0.js

4193
family
99.6079

92.0916
genus
262
node82.members.0.js

node83.members.0.js
597
100
genus

genus
91.3214
473
node84.members.0.js

80
genus
node85.members.0.js
2861

2018
order
80

family
80
2018

genus
80
2018
node88.members.0.js

167
order
100

167
family
100

genus
100
167
node91.members.0.js

100
order
2468

family
100
126

126
node94.members.0.js
100
genus

2342
100
family

genus
98
node96.members.0.js
2323

100
genus
19
node97.members.0.js

120
99.75
order

99.75
family
120

genus
100
node100.members.0.js
90

30
node101.members.0.js
98
genus

673
88.6256
order

family
100
80

genus
89
80
node104.members.0.js

88
family
197

80
genus
197
node106.members.0.js

family
80
396

genus
80
396
node108.members.0.js

3
100
order

family
100
3

node111.members.0.js
3
genus
100

13
95.1538
order

95.1538
family
13

88.5385
genus
13
node114.members.0.js

4
98.5
order

2
100
family

92
genus
2
node117.members.0.js

2
97
family

2
node119.members.0.js
genus
97

class
99.9169
903

99.9169
order
903

family
99.9169
903

100
genus
898
node123.members.0.js

80
genus
5
node124.members.0.js

99.7232
class
4136

99.7232
order
4136

4136
99.7232
family

genus
99.7232
4136
node128.members.0.js

13073
99.236
class

order
100
24

24
100
family

node132.members.0.js
24
genus
100

97.5775
order
3846

family
100
78

genus
100
78
node135.members.0.js

1474
81.7605
family

1474
node137.members.0.js
genus
81.7605

1099
family
99.1192

node139.members.0.js
4
100
genus

genus
100
node140.members.0.js
2

genus
99
98
node141.members.0.js

node142.members.0.js
11
95
genus

968
node143.members.0.js
genus
99

node144.members.0.js
16
genus
100

8
93
family

93
genus
8
node146.members.0.js

751
80
family

node148.members.0.js
751
80
genus

family
100
28

100
genus
28
node150.members.0.js

54
100
family

54
node152.members.0.js
genus
100

11
family
99

99
genus
9
node154.members.0.js

node155.members.0.js
2
98
genus

337
family
100

genus
100
3
node157.members.0.js

334
node158.members.0.js
genus
100

family
100
6

6
node160.members.0.js
99
genus

80
order
816

816
80
family

node163.members.0.js
816
80
genus

7
order
100

7
100
family

genus
100
node166.members.0.js
7

91
order
9

9
91
family

node169.members.0.js
9
91
genus

order
100
8371

family
100
8371

genus
100
node172.members.0.js
8371

155
95.7677
class

order
95.7677
155

33
family
80

node176.members.0.js
33
genus
80

family
87
5

87
genus
5
node178.members.0.js

family
99.2737
95

44
node180.members.0.js
80
genus

51
node181.members.0.js
99
genus

22
99.5909
family

node183.members.0.js
13
100
genus

node184.members.0.js
9
genus
80

93.1512
phylum
7491

class
99.4
15

order
100
6

6
100
family

100
genus
node189.members.0.js
6

9
99
order

99
family
9

9
node192.members.0.js
genus
99

class
98.6
10

10
98.6
order

10
family
98.6

genus
80
7
node196.members.0.js

genus
100
node197.members.0.js
3

20
class
100

20
order
100

20
family
100

genus
100
20
node201.members.0.js

94.1661
class
554

2
order
100

family
100
2

node205.members.0.js
2
100
genus

471
order
95.8217

100
family
3

node208.members.0.js
3
genus
100

84
family
117

genus
84
117
node210.members.0.js

family
80
351

80
genus
node212.members.0.js
351

80
order
81

81
80
family

80
genus
node215.members.0.js
81

1549
class
96.1194

100
order
90

90
100
family

90
node219.members.0.js
100
genus

94
order
5

80
family
5

5
node222.members.0.js
genus
80

46
order
100

3
80
family

80
genus
3
node225.members.0.js

43
100
family

100
genus
43
node227.members.0.js

5
84
order

82
family
5

5
node230.members.0.js
genus
80

90.3937
order
1105

1105
90.3937
family

node233.members.0.js
1105
90.3937
genus

order
95.5909
132

27
100
family

27
node236.members.0.js
100
genus

80
family
48

80
genus
48
node238.members.0.js

57
family
99.8947

7
node240.members.0.js
100
genus

genus
99.76
50
node241.members.0.js

2
100
order

2
family
100

node244.members.0.js
2
genus
100

80
order
164

80
family
164

node247.members.0.js
164
80
genus

2228
97.7724
class

order
98
198

family
98
198

genus
98
198
node251.members.0.js

32
order
100

32
100
family

genus
100
node254.members.0.js
4

100
genus
node255.members.0.js
28

225
97.0756
order

225
family
97.0756

19
node258.members.0.js
80
genus

genus
90.7861
187
node259.members.0.js

genus
95
19
node260.members.0.js

94.6295
order
359

family
80.2609
161

genus
80.2609
node263.members.0.js
161

80
family
9

9
node265.members.0.js
genus
80

189
100
family

100
genus
node267.members.0.js
189

order
80
1414

family
80
1414

80
genus
1414
node270.members.0.js

3115
80
class

80
order
3115

3115
80
family

node274.members.0.js
3115
80
genus

135
phylum
95.6

135
class
94.2963

52
80
order

52
family
80

80
genus
node279.members.0.js
52

order
100
83

83
100
family

100
genus
83
node282.members.0.js

phylum
99.8724
33300

4
class
96

4
96
order

96
family
4

genus
85
4
node287.members.0.js

class
100
93

order
100
93

93
family
100

genus
100
node291.members.0.js
20

44
node292.members.0.js
80
genus

100
genus
node293.members.0.js
29

33203
99.8725
class

order
99.8725
33203

33203
family
99.8725

99.6582
genus
33117
node297.members.0.js

80
genus
node298.members.0.js
86

phylum
80
2538

class
80
2538

order
80
2538

2538
80
family

2538
node303.members.0.js
genus
80
