# Supplementary material for: Fungal community profiles in agricultural soils of a long-term field trial under different tillage, fertilization and crop rotation conditions analyzed by high-throughput ITS-amplicon sequencing
Source: PLoS One. 2018 Apr 5;13(4):e0195345. doi: 10.1371/journal.pone.0195345 (PMC5886558; doi:10.1371/journal.pone.0195345)
Supplement: S2 File — (HTML) [file pone.0195345.s012.html]

Javascript must be enabled to view this page.

members
count
unassigned
score
rank

ITS1BC9.fastq\_final.fastq\_classified\_otusc\_clean


89580

100
89580
domain

34608
98.9806
phylum

100
8
class

100
8
order

family
8
100

99
8
node6.members.0.js
genus

class
96.2122
12045

order
189
99.7989

99.7989
189
family

genus
node10.members.0.js
98
38

100
151
genus
node11.members.0.js

2
100
order

100
2
family

2
100
node14.members.0.js
genus

4342
99.4051
order

100
9
family

100
9
node17.members.0.js
genus

family
26
100

node19.members.0.js
genus
26
100

80
6
family

80
6
node21.members.0.js
genus

100
742
family

666
99.976
node23.members.0.js
genus

node24.members.0.js
genus
76
100

3226
99.2591
family

node26.members.0.js
genus
100
541

399
91.2005
genus
node27.members.0.js

1943
80
genus
node28.members.0.js

genus
node29.members.0.js
92
343

27
88.2222
family

node31.members.0.js
genus
27
88.2222

99.8137
306
family

genus
node33.members.0.js
280
96

10
94.3
genus
node34.members.0.js

genus
node35.members.0.js
100
16

order
1507
89.3895

250
100
family

genus
node38.members.0.js
250
89

family
1074
80

genus
node40.members.0.js
1074
80

family
88.1967
183

genus
node42.members.0.js
3
100

node43.members.0.js
genus
180
80

order
99.8489
1026

31
80
family

node46.members.0.js
genus
80
31

family
960
100

genus
node48.members.0.js
100
4

node49.members.0.js
genus
956
98

family
2
100

genus
node51.members.0.js
99
2

family
100
33

node53.members.0.js
genus
100
33

order
123
97

123
97
family

node56.members.0.js
genus
97
123

order
80
4759

family
4759
80

genus
node59.members.0.js
4759
80

order
100
67

67
100
family

node62.members.0.js
genus
100
67

order
100
14

family
14
91

genus
node65.members.0.js
91
14

order
16
98.125

family
16
98.125

93.625
16
genus
node68.members.0.js

class
1001
99.8521

99.7992
1001
order

99.7992
1001
family

genus
node72.members.0.js
53
80

100
948
genus
node73.members.0.js

95.155
613
class

95.155
613
order

family
100
2

100
2
node77.members.0.js
genus

family
96
7

86
2
genus
node79.members.0.js

100
5
genus
node80.members.0.js

family
82.5265
604

genus
node82.members.0.js
604
82.5265

3356
99.6451
class

3356
99.6451
order

99.6451
3356
family

3356
99.6451
node86.members.0.js
genus

class
99.1035
7890

2820
99.9989
order

family
99.9989
2820

94
3
node90.members.0.js
genus

2817
100
genus
node91.members.0.js

100
37
order

100
37
family

genus
node94.members.0.js
100
37

30
100
order

family
30
100

genus
node97.members.0.js
30
100

1171
80
order

80
1171
family

1171
80
node100.members.0.js
genus

98.333
3832
order

135
100
family

100
135
node103.members.0.js
genus

99.6364
187
family

node105.members.0.js
genus
184
99.3261

98
3
node106.members.0.js
genus

80.95
1241
family

80.95
1241
node108.members.0.js
genus

family
890
99.409

100
29
genus
node110.members.0.js

99
526
node111.members.0.js
genus

3
80
node112.members.0.js
genus

99
286
node113.members.0.js
genus

genus
node114.members.0.js
99.9259
27

genus
node115.members.0.js
95
19

family
752
97.1915

node117.members.0.js
genus
680
97

99
69
genus
node118.members.0.js

node119.members.0.js
genus
98
3

80
457
family

genus
node121.members.0.js
80
457

62
99.9516
family

node123.members.0.js
genus
62
99.9516

family
100
108

genus
node125.members.0.js
108
100

80
4972
class

order
4972
80

family
80
4972

genus
node129.members.0.js
4972
80

class
93.1132
433

order
433
93.1132

8
80
family

80
8
genus
node133.members.0.js

family
87
177

genus
node135.members.0.js
177
87

93.3831
154
family

node137.members.0.js
genus
99
75

79
80
node138.members.0.js
genus

family
94
99.2766

80
33
node140.members.0.js
genus

100
61
node141.members.0.js
genus

97.6972
4290
class

order
3639
99.8752

family
100
3503

genus
node145.members.0.js
100
3503

123
97
family

genus
node147.members.0.js
123
80

80
13
family

13
80
node149.members.0.js
genus

order
85.3794
651

651
85.3794
family

genus
node152.members.0.js
651
85.3794

phylum
3856
80

3856
80
class

3856
80
order

family
3856
80

80
3856
node157.members.0.js
genus

phylum
249
93.3695

7
80
class

order
7
80

7
80
family

7
80
genus
node162.members.0.js

93.7562
242
class

order
7
100

100
7
family

7
100
node166.members.0.js
genus

order
103
80

family
103
80

103
80
genus
node169.members.0.js

order
99.9545
132

99.9545
132
family

132
99.9545
genus
node172.members.0.js

40123
99.8376
phylum

99.8387
39982
class

99.8387
39982
order

99.8387
39982
family

223
80
node177.members.0.js
genus

node178.members.0.js
genus
39759
99.3763

class
99.3333
12

order
12
99.3333

99.3333
12
family

node182.members.0.js
genus
12
99.3333

class
91.3333
9

91.3333
9
order

family
91.3333
9

80
9
node186.members.0.js
genus

120
100
class

order
100
120

family
100
120

100
37
node190.members.0.js
genus

100
42
node191.members.0.js
genus

genus
node192.members.0.js
80
31

10
100
node193.members.0.js
genus

phylum
98.9067
4960

142
97
class

142
97
order

142
97
family

genus
node198.members.0.js
97
142

80
68
class

order
80
68

family
68
80

68
80
node202.members.0.js
genus

4750
98.4882
class

418
99.4593
order

family
402
99.4378

88
80
genus
node206.members.0.js

genus
node207.members.0.js
314
99.1497

16
100
family

node209.members.0.js
genus
100
16

4185
98.4335
order

family
172
80

80
172
node212.members.0.js
genus

98.931
3810
family

1794
98.0663
genus
node214.members.0.js

node215.members.0.js
genus
96.6769
65

node216.members.0.js
genus
96.8797
1937

node217.members.0.js
genus
14
80

92.3153
203
family

genus
node219.members.0.js
92.3153
203

80
100
order

100
80
family

80
100
node222.members.0.js
genus

order
47
96.2553

family
47
96.2553

node225.members.0.js
genus
47
96.2553

phylum
5784
92.1885

class
1319
95.7847

order
99.8795
83

80
5
family

node230.members.0.js
genus
80
5

family
78
100

genus
node232.members.0.js
78
100

676
90.0828
order

90
668
family

genus
node235.members.0.js
90
668

97
8
family

8
96
node237.members.0.js
genus

order
98.1443
291

family
96
13

node240.members.0.js
genus
13
96

family
100
100

node242.members.0.js
genus
100
100

4
86
family

4
86
node244.members.0.js
genus

family
19
100

node246.members.0.js
genus
100
19

80
36
family

36
80
node248.members.0.js
genus

100
119
family

100
50
node250.members.0.js
genus

51
100
genus
node251.members.0.js

genus
node252.members.0.js
100
18

order
100
10

family
10
100

node255.members.0.js
genus
10
100

order
230
80

80
230
family

node258.members.0.js
genus
230
80

order
100
10

family
100
10

10
100
node261.members.0.js
genus

19
88.1579
order

family
19
88.1579

genus
node264.members.0.js
4
100

80
15
node265.members.0.js
genus

97.769
1411
class

order
11
100

family
100
11

9
100
genus
node269.members.0.js

genus
node270.members.0.js
100
2

order
98
123

family
123
98

node273.members.0.js
genus
98
123

order
95.2432
481

100
289
family

genus
node276.members.0.js
100
289

family
80
16

80
16
genus
node278.members.0.js

80
176
family

80
176
genus
node280.members.0.js

99.625
208
order

99.625
208
family

95
12
node283.members.0.js
genus

32
80
genus
node284.members.0.js

node285.members.0.js
genus
95.2805
164

order
80
588

80
588
family

genus
node288.members.0.js
588
80

80
2833
class

2833
80
order

family
80
2833

node292.members.0.js
genus
2833
80

class
18
100

order
99
18

18
99
family

node296.members.0.js
genus
18
99

class
100
24

100
18
order

family
18
100

node300.members.0.js
genus
18
100

order
100
6

6
100
family

6
100
node303.members.0.js
genus

100
3
class

order
3
100

family
3
100

node307.members.0.js
genus
3
100

100
4
class

order
100
4

100
4
family

node311.members.0.js
genus
4
100

4
100
class

order
100
4

family
100
4

100
4
node315.members.0.js
genus

168
93.5179
class

order
93.0476
168

family
97
80

97
80
genus
node319.members.0.js

family
71
84

84
71
node321.members.0.js
genus
