# Supplementary material for: Fungal community profiles in agricultural soils of a long-term field trial under different tillage, fertilization and crop rotation conditions analyzed by high-throughput ITS-amplicon sequencing
Source: PLoS One. 2018 Apr 5;13(4):e0195345. doi: 10.1371/journal.pone.0195345 (PMC5886558; doi:10.1371/journal.pone.0195345)
Supplement: S3 File — (HTML) [file pone.0195345.s013.html]

Javascript must be enabled to view this page.

members
count
unassigned
score
rank

ITS1BC6.fastq\_final.fastq\_classified\_otusc\_clean


79832

79832
domain
100

98.224
1656
phylum

95.7613
1588
class

111
order
99.3874

99.3874
family
111

111
93.2613
node6.members.0.js
genus

67
order
80

80
67
family

67
genus
80
node9.members.0.js

227
order
94.2203

83.6471
family
17

17
genus
node12.members.0.js
83.6471

family
210
95.0762

210
95.0762
node14.members.0.js
genus

95.6484
order
1183

95.8988
1028
family

7
node17.members.0.js
92.8571
genus

genus
80
node18.members.0.js
115

node19.members.0.js
95.02
genus
750

genus
node20.members.0.js
99.5385
156

family
51
80

51
node22.members.0.js
80
genus

91.4615
family
104

104
genus
91.4615
node24.members.0.js

80
45
class

45
order
80

45
family
80

45
genus
node28.members.0.js
80

96
23
class

96
order
23

23
family
96

genus
96
node32.members.0.js
23

2829
phylum
80

80
2829
class

80
order
2829

family
2829
80

genus
80
node37.members.0.js
2829

phylum
31577
99.8212

100
class
9

order
9
100

9
family
100

9
genus
node42.members.0.js
100

class
31269
99.8228

31269
order
99.8228

31269
family
99.8228

376
genus
80
node46.members.0.js

30893
genus
99.565
node47.members.0.js

class
281
100

100
order
281

family
281
100

17
genus
node51.members.0.js
100

genus
node52.members.0.js
100
135

100
node53.members.0.js
genus
2

genus
node54.members.0.js
80
98

29
100
node55.members.0.js
genus

91.8333
class
18

order
18
91.8333

91.8333
family
18

genus
80
node59.members.0.js
5

13
82.2308
node60.members.0.js
genus

35757
phylum
99.382

class
10593
99.1476

4792
order
98.4622

99.8831
77
family

genus
99
node65.members.0.js
9

68
node66.members.0.js
100
genus

99.96
1176
family

genus
node68.members.0.js
86
19

genus
100
node69.members.0.js
6

99.9183
node70.members.0.js
genus
1151

8
family
100

genus
100
node72.members.0.js
8

1601
family
99.6771

node74.members.0.js
100
genus
5

12
node75.members.0.js
80
genus

970
99
node76.members.0.js
genus

genus
99
node77.members.0.js
517

62
100
node78.members.0.js
genus

29
genus
95
node79.members.0.js

6
genus
node80.members.0.js
99.6667

98.4091
88
family

node82.members.0.js
98.4091
genus
88

family
67
100

genus
100
node84.members.0.js
67

80
family
6

genus
80
node86.members.0.js
6

80.7308
family
1330

node88.members.0.js
80.7308
genus
1330

family
439
80

node90.members.0.js
80
genus
439

order
1135
80

family
1135
80

1135
80
node93.members.0.js
genus

100
order
7

100
7
family

node96.members.0.js
100
genus
7

4632
order
100

100
family
4632

4632
100
node99.members.0.js
genus

order
27
100

100
27
family

node102.members.0.js
100
genus
27

303
class
95.5116

95.5116
order
303

family
183
98.9727

43
node106.members.0.js
100
genus

140
genus
node107.members.0.js
80

5
family
80

80
node109.members.0.js
genus
5

87.7652
115
family

genus
node111.members.0.js
99
5

node112.members.0.js
80
genus
110

4141
class
80

4141
order
80

4141
family
80

4141
genus
80
node116.members.0.js

12912
class
98.3879

100
23
order

23
family
100

23
node120.members.0.js
99
genus

98
order
4

family
4
98

4
genus
node123.members.0.js
90

80
order
3764

80
3764
family

3764
node126.members.0.js
80
genus

order
2
100

100
2
family

genus
node129.members.0.js
92
2

order
180
99.8889

family
180
99.8889

160
node132.members.0.js
100
genus

20
genus
98
node133.members.0.js

order
11
100

91
11
family

11
91
node136.members.0.js
genus

order
1711
99.9942

family
281
100

genus
node139.members.0.js
100
281

100
1428
family

genus
97
node141.members.0.js
9

node142.members.0.js
98
genus
1419

family
2
80

genus
node144.members.0.js
80
2

99.8252
103
order

99.8252
103
family

103
genus
99.8252
node147.members.0.js

6730
order
99.7288

6240
family
99.7188

4483
genus
node150.members.0.js
80

2
node151.members.0.js
100
genus

genus
100
node152.members.0.js
1056

genus
node153.members.0.js
91.4076
471

228
genus
92
node154.members.0.js

family
23
88

node156.members.0.js
88
genus
23

100
7
family

genus
node158.members.0.js
100
7

100
3
family

genus
node160.members.0.js
100
3

400
family
100

node162.members.0.js
100
genus
65

335
node163.members.0.js
99.9642
genus

99
57
family

25
genus
node165.members.0.js
97.72

genus
96
node166.members.0.js
23

9
100
node167.members.0.js
genus

97.3333
18
order

97.3333
18
family

genus
node170.members.0.js
91.7778
18

order
366
95.4235

family
35
80

35
genus
80
node173.members.0.js

family
69
100

69
genus
node175.members.0.js
89

262
family
90.1069

100
node177.members.0.js
genus
36

80
node178.members.0.js
genus
226

100
class
18

18
order
100

family
18
100

node182.members.0.js
100
genus
13

5
80
node183.members.0.js
genus

96.2949
468
class

order
468
96.2949

family
12
86

genus
node187.members.0.js
86
12

85.1272
family
456

456
node189.members.0.js
85.1272
genus

98.0397
class
2342

order
2342
98.0397

98.0397
family
2342

genus
98.0397
node193.members.0.js
2342

4245
class
97.5458

order
2
99

family
2
99

2
genus
80
node197.members.0.js

order
51
80

family
51
80

51
genus
node200.members.0.js
80

98.8012
3787
order

317
family
80

80
node203.members.0.js
genus
317

99
family
3101

3101
genus
99
node205.members.0.js

369
family
97.0244

genus
node207.members.0.js
80
318

node208.members.0.js
97.1765
genus
51

405
order
85.3975

family
405
85.3975

85.3975
node211.members.0.js
genus
405

735
class
99.9565

100
order
2

100
family
2

2
node215.members.0.js
80
genus

99.9482
733
order

731
family
100

731
genus
100
node218.members.0.js

family
2
80

genus
node220.members.0.js
80
2

7916
phylum
93.951

class
35
100

100
7
order

family
7
100

genus
100
node225.members.0.js
7

28
order
100

family
28
100

28
genus
100
node228.members.0.js

class
262
95.9466

100
order
3

100
family
3

3
genus
node232.members.0.js
100

order
203
97.8719

27
family
84

genus
node235.members.0.js
84
27

family
176
80

genus
80
node237.members.0.js
176

order
56
80

80
family
56

56
genus
node240.members.0.js
80

3481
class
96.6145

99.7778
order
27

9
family
80

9
genus
node244.members.0.js
80

100
family
18

18
genus
node246.members.0.js
100

order
2831
95.6348

100
family
24

100
node249.members.0.js
genus
24

family
25
86

25
86
node251.members.0.js
genus

family
2
100

node253.members.0.js
100
genus
2

100
family
42

8
100
node255.members.0.js
genus

genus
node256.members.0.js
100
34

100
15
family

15
node258.members.0.js
96
genus

family
2723
80

2723
80
node260.members.0.js
genus

order
4
82

4
family
81

4
genus
80
node263.members.0.js

80
237
order

family
237
80

237
genus
80
node266.members.0.js

93
380
order

93
family
380

93
node269.members.0.js
genus
380

100
2
order

family
2
100

genus
node272.members.0.js
94
2

class
1524
96.4055

order
226
98

98
226
family

226
98
node276.members.0.js
genus

581
order
80

581
family
80

581
80
node279.members.0.js
genus

91.9543
525
order

100
156
family

156
genus
node282.members.0.js
100

80
3
family

3
genus
node284.members.0.js
80

366
family
80.9836

366
genus
80.9836
node286.members.0.js

180
order
94.5667

180
family
94.5667

node289.members.0.js
95
genus
13

10
genus
80
node290.members.0.js

157
genus
node291.members.0.js
90.7707

12
order
93.75

80
5
family

80
node294.members.0.js
genus
5

7
family
100

genus
node296.members.0.js
100
2

genus
100
node297.members.0.js
5

6
class
98

6
order
98

98
family
6

6
genus
80
node301.members.0.js

59
class
100

order
59
99

99
family
59

59
node305.members.0.js
99
genus

80
2549
class

80
order
2549

2549
family
80

genus
node309.members.0.js
80
2549

96.3093
97
phylum

96.2887
class
97

99.575
80
order

99.575
80
family

genus
node314.members.0.js
99.575
80

17
order
80

17
family
80

genus
node317.members.0.js
80
17
