# Supplementary material for: Fungal community profiles in agricultural soils of a long-term field trial under different tillage, fertilization and crop rotation conditions analyzed by high-throughput ITS-amplicon sequencing
Source: PLoS One. 2018 Apr 5;13(4):e0195345. doi: 10.1371/journal.pone.0195345 (PMC5886558; doi:10.1371/journal.pone.0195345)
Supplement: S4 File — (HTML) [file pone.0195345.s014.html]

Javascript must be enabled to view this page.

members
count
unassigned
score
rank

ITS1BC12.fastq\_final.fastq\_classified\_otusc\_clean


97474

97474
100
domain

80
3228
phylum

3228
80
class

3228
80
order

3228
80
family

80
genus
node6.members.0.js
3228

93.025
160
phylum

class
160
93.025

order
100
5

family
5
100

100
genus
node11.members.0.js
5

100
93
order

family
100
93

93
100
node14.members.0.js
genus

80
62
order

family
62
80

62
80
node17.members.0.js
genus

phylum
4318
91.1568

1056
95.3617
class

100
12
order

family
8
100

8
node22.members.0.js
genus
86

family
100
4

2
node24.members.0.js
genus
100

genus
node25.members.0.js
100
2

585
91.5487
order

family
145
100

145
100
node28.members.0.js
genus

family
440
81.2727

node30.members.0.js
genus
81.2727
440

order
367
80

family
80
367

367
80
genus
node33.members.0.js

order
56
99

family
56
99

52
94.5385
genus
node36.members.0.js

genus
node37.members.0.js
80
4

order
36
98

family
36
98

36
node40.members.0.js
genus
98

class
92.3222
90

order
57
90.7368

33
84
family

33
node44.members.0.js
genus
84

80
24
family

node46.members.0.js
genus
80
24

order
88.6923
26

family
88.6923
26

21
80
node49.members.0.js
genus

100
genus
node50.members.0.js
5

order
7
80

7
80
family

80
genus
node53.members.0.js
7

80
1931
class

80
1931
order

family
1931
80

1931
genus
node57.members.0.js
80

class
99.8235
34

28
100
order

100
28
family

28
node61.members.0.js
genus
100

6
99
order

99
6
family

99
genus
node64.members.0.js
6

class
98
11

98
11
order

family
98
11

11
80
node68.members.0.js
genus

1188
92.2795
class

90.2548
314
order

family
100
2

100
node72.members.0.js
genus
2

143
80
family

80
genus
node74.members.0.js
143

family
100
75

71
genus
node76.members.0.js
99

100
node77.members.0.js
genus
4

family
100
53

node79.members.0.js
genus
100
38

15
node80.members.0.js
genus
100

family
97
6

6
node82.members.0.js
genus
97

100
22
family

100
genus
node84.members.0.js
17

5
genus
node85.members.0.js
100

86
13
family

13
genus
node87.members.0.js
86

142
91.0775
order

family
91.0775
142

142
91.0775
node90.members.0.js
genus

order
100
46

10
80
family

10
80
node93.members.0.js
genus

family
100
36

genus
node95.members.0.js
100
36

80
686
order

80
686
family

80
genus
node98.members.0.js
686

8
100
class

order
8
99

family
99
8

8
genus
node102.members.0.js
99

99.882
58646
phylum

93
6
class

order
6
93

family
6
93

6
genus
node107.members.0.js
80

class
100
222

order
100
222

222
100
family

node111.members.0.js
genus
100
167

100
node112.members.0.js
genus
17

100
genus
node113.members.0.js
21

17
80
genus
node114.members.0.js

class
58418
99.8822

99.8822
58418
order

99.8822
58418
family

genus
node118.members.0.js
99.8427
57829

genus
node119.members.0.js
80
589

28293
98.8438
phylum

2010
97.3891
class

order
97.3891
2010

family
97.3891
2010

node124.members.0.js
genus
97.3891
2010

456
89.5066
class

456
89.5066
order

17
93.8824
family

4
100
node128.members.0.js
genus

13
80
genus
node129.members.0.js

family
80
7

7
80
node131.members.0.js
genus

family
88.3679
212

37
node133.members.0.js
genus
99

175
80
node134.members.0.js
genus

family
87
220

220
genus
node136.members.0.js
87

class
4534
98.4658

order
97.9173
2623

99.625
128
family

128
node140.members.0.js
genus
99.625

family
212
97.8491

3
99
genus
node142.members.0.js

209
genus
node143.members.0.js
97.5981

80.1629
1160
family

80.1629
node145.members.0.js
genus
1160

352
99.9318
family

32
86
genus
node147.members.0.js

11
99.4545
node148.members.0.js
genus

genus
node149.members.0.js
99.8835
309

97
99.2165
family

80
node151.members.0.js
genus
4

93
node152.members.0.js
genus
100

family
41
100

41
genus
node154.members.0.js
100

99.5779
353
family

genus
node156.members.0.js
100
112

99
node157.members.0.js
genus
133

node158.members.0.js
genus
88
4

5
genus
node159.members.0.js
100

99
genus
node160.members.0.js
73

node161.members.0.js
genus
95
26

family
39
84.6154

80
genus
node163.members.0.js
39

family
241
80

241
80
genus
node165.members.0.js

80
821
order

family
80
821

80
genus
node168.members.0.js
821

100
25
order

25
100
family

genus
node171.members.0.js
100
25

99.9981
1051
order

99.9981
1051
family

94
genus
node174.members.0.js
2

node175.members.0.js
genus
100
1049

order
89.8182
11

89.8182
11
family

genus
node178.members.0.js
84
7

4
86
genus
node179.members.0.js

100
3
order

3
100
family

genus
node182.members.0.js
100
3

class
96
2

2
96
order

family
2
96

2
96
node186.members.0.js
genus

94.4937
1347
class

order
1347
94.4937

family
1347
81.3823

1347
81.3823
genus
node190.members.0.js

11909
98.412
class

96.1241
572
order

363
89.7961
family

308
80
genus
node194.members.0.js

100
node195.members.0.js
genus
53

96
genus
node196.members.0.js
2

99.9882
170
family

node198.members.0.js
genus
88.9509
163

99
genus
node199.members.0.js
7

family
80
39

80
genus
node201.members.0.js
39

99.5282
5772
order

39
88.2564
family

39
88.2564
genus
node204.members.0.js

family
6
100

100
node206.members.0.js
genus
6

100
1442
family

node208.members.0.js
genus
100
15

node209.members.0.js
genus
99.9846
1427

184
100
family

31
100
node211.members.0.js
genus

141
96
genus
node212.members.0.js

100
node213.members.0.js
genus
12

99.3839
4082
family

386
91
node215.members.0.js
genus

node216.members.0.js
genus
100
521

176
92
genus
node217.members.0.js

2999
genus
node218.members.0.js
80

80
11
family

node220.members.0.js
genus
80
11

100
8
family

100
genus
node222.members.0.js
8

order
1695
100

family
100
1672

1672
98
genus
node225.members.0.js

23
100
family

23
100
genus
node227.members.0.js

order
80
3516

80
3516
family

80
genus
node230.members.0.js
3516

order
4
100

91
4
family

91
genus
node233.members.0.js
4

order
5
96.4

family
5
96.4

93
node236.members.0.js
genus
3

80
node237.members.0.js
genus
2

11
99.4545
order

family
99.4545
11

8
genus
node240.members.0.js
99

87
node241.members.0.js
genus
3

order
98.5
24

98.5
24
family

24
95
genus
node244.members.0.js

94.6
5
order

94.6
5
family

2
92
node247.members.0.js
genus

90
genus
node248.members.0.js
3

order
161
99.5963

99.5963
161
family

96
100
genus
node251.members.0.js

65
98
genus
node252.members.0.js

144
98.4375
order

144
98.4375
family

144
genus
node255.members.0.js
98.4375

class
100
14

14
100
order

family
100
14

80
genus
node259.members.0.js
9

99
genus
node260.members.0.js
3

100
genus
node261.members.0.js
2

class
4087
80

order
4087
80

4087
80
family

4087
genus
node265.members.0.js
80

class
2831
98.177

order
99
2

99
2
family

99
genus
node269.members.0.js
2

514
89.8949
order

89.8949
514
family

genus
node272.members.0.js
89.8949
514

9
80
order

family
9
80

80
node275.members.0.js
genus
9

2306
99.8356
order

2
80
family

80
node278.members.0.js
genus
2

family
2143
99.9538

99.9538
genus
node280.members.0.js
2143

97.354
161
family

genus
node282.members.0.js
80
79

node283.members.0.js
genus
97.6951
82

class
1103
100

100
2
order

2
100
family

node287.members.0.js
genus
80
2

100
1101
order

family
1101
100

100
genus
node290.members.0.js
1101

phylum
98.953
2829

2766
97.7437
class

order
97.6423
2631

80
112
family

112
80
genus
node295.members.0.js

98.2044
2466
family

94.4464
node297.members.0.js
genus
905

118
80
genus
node298.members.0.js

1331
95.3043
genus
node299.members.0.js

112
98.0179
genus
node300.members.0.js

53
94.7358
family

node302.members.0.js
genus
94.7358
53

order
48
98.5833

45
98.4889
family

6
node305.members.0.js
genus
80

node306.members.0.js
genus
97.5897
39

3
100
family

100
node308.members.0.js
genus
3

95.0426
47
order

family
95.9767
43

node311.members.0.js
genus
95.9767
43

4
85
family

4
genus
node313.members.0.js
85

order
80
40

40
80
family

node316.members.0.js
genus
80
40

class
22
96

22
96
order

22
96
family

22
genus
node320.members.0.js
96

41
80
class

41
80
order

family
41
80

node324.members.0.js
genus
80
41
