# Supplementary material for: Fungal community profiles in agricultural soils of a long-term field trial under different tillage, fertilization and crop rotation conditions analyzed by high-throughput ITS-amplicon sequencing
Source: PLoS One. 2018 Apr 5;13(4):e0195345. doi: 10.1371/journal.pone.0195345 (PMC5886558; doi:10.1371/journal.pone.0195345)
Supplement: S5 File — (HTML) [file pone.0195345.s015.html]

Javascript must be enabled to view this page.

members
count
unassigned
score
rank

ITS1BC8.fastq\_classified\_otusc\_clean


76629

76629
domain
100

99.3887
44775
phylum

98.9913
class
13629

85.056
3448
order

85.056
family
3448

46
99
node6.members.0.js
genus

3031
80
genus
node7.members.0.js

371
100
genus
node8.members.0.js

72
order
98.3333

98.3333
72
family

node11.members.0.js
genus
100
52

20
80
node12.members.0.js
genus

order
60
99.2

family
60
99.2

60
93.8
node15.members.0.js
genus

3995
order
99.1277

family
158
100

158
100
node18.members.0.js
genus

98.7884
family
3143

node20.members.0.js
genus
80
2330

8
89
genus
node21.members.0.js

96
167
node22.members.0.js
genus

node23.members.0.js
genus
638
93

80
3
family

node25.members.0.js
genus
3
80

family
141
100

44
99
node27.members.0.js
genus

node28.members.0.js
genus
84
85

genus
node29.members.0.js
12
100

92
family
14

14
91
node31.members.0.js
genus

99.9496
536
family

97.3636
11
node33.members.0.js
genus

node34.members.0.js
genus
525
100

93.5348
402
order

90.6901
71
family

node37.members.0.js
genus
98.0345
29

node38.members.0.js
genus
37
80

5
100
genus
node39.members.0.js

99.7548
family
208

157
96
node41.members.0.js
genus

51
84
node42.members.0.js
genus

family
123
80

node44.members.0.js
genus
80
123

order
176
100

100
family
2

node47.members.0.js
genus
2
99

100
174
family

174
100
node49.members.0.js
genus

order
3514
99.928

100
family
3

100
3
node52.members.0.js
genus

family
23
80

80
23
node54.members.0.js
genus

family
3488
100

node56.members.0.js
genus
1952
98

96
1536
node57.members.0.js
genus

27
order
100

family
27
100

11
80
genus
node60.members.0.js

16
100
node61.members.0.js
genus

80
1696
order

80
1696
family

1696
80
node64.members.0.js
genus

100
8
order

100
8
family

node67.members.0.js
genus
8
100

order
227
100

227
family
100

227
100
genus
node70.members.0.js

100
4
order

4
family
88

genus
node73.members.0.js
4
88

97.0746
class
1407

100
3
order

3
family
100

3
100
node77.members.0.js
genus

order
1400
97.0686

family
1400
97.0686

node80.members.0.js
genus
97.0686
1400

4
order
97

97
family
4

node83.members.0.js
genus
97
4

80
class
4957

4957
order
80

family
4957
80

genus
node87.members.0.js
80
4957

98.6923
26
class

order
26
98.6923

8
family
99.625

5
99
genus
node91.members.0.js

genus
node92.members.0.js
3
80

98.2778
18
family

5
99
node94.members.0.js
genus

node95.members.0.js
genus
13
80

93
class
10

10
order
93

family
10
82

82
10
node99.members.0.js
genus

99.1317
class
20464

88.5385
order
13

88.5385
13
family

node103.members.0.js
genus
4
80

91
9
node104.members.0.js
genus

95.3004
order
4255

family
2085
80

2085
80
node107.members.0.js
genus

95.8793
family
58

node109.members.0.js
genus
95
58

96
family
5

96
5
node111.members.0.js
genus

100
family
11

100
11
node113.members.0.js
genus

92
family
16

node115.members.0.js
genus
92
16

family
727
99.0179

node117.members.0.js
genus
170
100

node118.members.0.js
genus
80
17

genus
node119.members.0.js
98
357

node120.members.0.js
genus
89
7

100
82
node121.members.0.js
genus

node122.members.0.js
genus
35
94

98
3
node123.members.0.js
genus

56
99
genus
node124.members.0.js

176
family
100

100
176
genus
node126.members.0.js

family
1177
99.8675

100
1165
node128.members.0.js
genus

87
12
node129.members.0.js
genus

99.9966
14025
order

14025
family
99.9833

node132.members.0.js
genus
80
6

node133.members.0.js
genus
99.988
14019

order
5
99

5
family
99

genus
node136.members.0.js
99
5

80
2136
order

80
2136
family

node139.members.0.js
genus
2136
80

100
30
order

100
30
family

30
100
node142.members.0.js
genus

class
1611
99.9677

1611
order
99.9677

1611
family
99.9677

genus
node146.members.0.js
99.9677
1611

100
class
15

15
order
100

family
15
100

93.4667
15
node150.members.0.js
genus

98.5809
2656
class

order
2025
98.244

88.6742
132
family

96
15
node154.members.0.js
genus

node155.members.0.js
genus
92.7273
11

node156.members.0.js
genus
17
96

80
89
genus
node157.members.0.js

family
65
80

node159.members.0.js
genus
80
65

family
1814
96

genus
node161.members.0.js
1814
96

99
family
14

14
93
node163.members.0.js
genus

228
order
90

90
family
228

228
80
node166.members.0.js
genus

99
order
403

family
403
99

403
99
node169.members.0.js
genus

95.0204
phylum
5398

2
class
100

100
order
2

100
family
2

node174.members.0.js
genus
100
2

99.5122
41
class

96
order
5

96
family
5

96
5
genus
node178.members.0.js

36
order
100

100
36
family

node181.members.0.js
genus
36
100

class
1631
99.6162

98
order
107

98
family
107

node185.members.0.js
genus
98
107

100
1336
order

100
family
1336

1336
100
node188.members.0.js
genus

97.761
159
order

family
29
92.7931

node191.members.0.js
genus
29
92.7931

100
9
family

100
7
genus
node193.members.0.js

node194.members.0.js
genus
2
100

100
4
family

node196.members.0.js
genus
100
4

2
family
81

node198.members.0.js
genus
2
80

99
64
family

genus
node200.members.0.js
64
99

84.6667
family
12

node202.members.0.js
genus
84.6667
12

family
39
99.5385

genus
node204.members.0.js
21
100

node205.members.0.js
genus
18
99

80
19
order

19
family
80

80
19
genus
node208.members.0.js

100
order
3

family
3
100

node211.members.0.js
genus
100
3

order
7
100

family
7
100

node214.members.0.js
genus
7
100

100
class
13

100
13
order

family
13
100

13
100
node218.members.0.js
genus

80
2245
class

2245
order
80

family
2245
80

node222.members.0.js
genus
2245
80

88.1651
630
class

100
order
204

80
family
204

204
80
node226.members.0.js
genus

80
426
order

80
426
family

genus
node229.members.0.js
426
80

7
class
100

100
order
7

7
family
100

100
7
genus
node233.members.0.js

829
class
96.5513

94
order
118

94
family
118

node237.members.0.js
genus
94
118

89.0723
166
order

89.0723
166
family

145
82.2828
node240.members.0.js
genus

node241.members.0.js
genus
80
21

80
order
255

80
family
255

node244.members.0.js
genus
255
80

99.9841
251
order

4
family
90

90
4
node247.members.0.js
genus

family
247
100

247
100
genus
node249.members.0.js

39
order
98.6667

family
39
98.6667

80
4
node252.members.0.js
genus

35
100
node253.members.0.js
genus

35
phylum
97.8571

35
class
97.8571

35
order
97.8571

97.8571
35
family

97.8571
35
node258.members.0.js
genus

97.6338
phylum
71

80
class
2

80
2
order

2
family
80

node263.members.0.js
genus
2
80

97.2029
69
class

order
11
99.4545

99
family
4

99
4
node267.members.0.js
genus

family
7
99.7143

node269.members.0.js
genus
7
80

order
7
88

88
family
7

node272.members.0.js
genus
88
7

97.9412
51
order

100
family
2

node275.members.0.js
genus
100
2

family
49
97.8571

node277.members.0.js
genus
100
14

genus
node278.members.0.js
35
92

99.427
phylum
20380

12
class
98.6667

order
12
98.6667

98.6667
family
12

12
98.6667
node283.members.0.js
genus

class
6
90

order
6
90

90
family
6

80
6
node287.members.0.js
genus

100
139
class

139
order
100

139
family
99.5827

genus
node291.members.0.js
96
100

node292.members.0.js
genus
14
100

29
80
node293.members.0.js
genus

20223
class
99.4147

99.4147
order
20223

99.4147
family
20223

node297.members.0.js
genus
98.7403
20089

node298.members.0.js
genus
80
134

phylum
5970
80

80
class
5970

order
5970
80

5970
family
80

80
5970
node303.members.0.js
genus
