# Supplementary material for: Fungal community profiles in agricultural soils of a long-term field trial under different tillage, fertilization and crop rotation conditions analyzed by high-throughput ITS-amplicon sequencing
Source: PLoS One. 2018 Apr 5;13(4):e0195345. doi: 10.1371/journal.pone.0195345 (PMC5886558; doi:10.1371/journal.pone.0195345)
Supplement: S6 File — (HTML) [file pone.0195345.s016.html]

Javascript must be enabled to view this page.

members
count
unassigned
score
rank

ITS1BC9.fastq\_classified\_otusc\_clean


72141

72141
100
domain

phylum
8462
98.7873

class
5777
99.7078

order
80
62

family
62
80

node6.members.0.js
80
62
genus

order
309
91

309
91
family

genus
node9.members.0.js
91
309

order
99.2039
103

family
12
83

node12.members.0.js
12
80
genus

family
13
96

genus
13
92
node14.members.0.js

2
80
family

node16.members.0.js
80
2
genus

family
36
100

genus
33
100
node18.members.0.js

node19.members.0.js
3
100
genus

family
4
100

genus
4
100
node21.members.0.js

family
99
3

genus
99
3
node23.members.0.js

100
33
family

genus
33
99
node25.members.0.js

order
5264
100

family
100
5264

node28.members.0.js
100
5264
genus

order
100
39

family
80
3

genus
node31.members.0.js
80
3

100
36
family

36
100
node33.members.0.js
genus

class
41
100

100
41
order

41
100
family

genus
100
41
node37.members.0.js

1526
96.576
class

97.9778
45
order

family
45
97.9778

genus
node41.members.0.js
2
100

genus
node42.members.0.js
80
7

100
36
node43.members.0.js
genus

218
93
order

93
218
family

genus
node46.members.0.js
93
218

501
94.2355
order

family
94.2355
501

genus
64
80
node49.members.0.js

node50.members.0.js
437
88.8719
genus

498
98.4618
order

family
100
452

genus
100
2
node53.members.0.js

450
100
node54.members.0.js
genus

family
80
40

genus
node56.members.0.js
80
40

family
90
6

node58.members.0.js
90
6
genus

80
264
order

80
264
family

genus
80
264
node61.members.0.js

class
4
100

order
100
4

100
4
family

node65.members.0.js
100
4
genus

class
99.2642
53

order
100
40

100
40
family

34
100
node69.members.0.js
genus

genus
6
80
node70.members.0.js

order
13
97

family
97
13

13
97
node73.members.0.js
genus

100
4
class

order
100
4

family
4
100

4
100
node77.members.0.js
genus

class
482
99.4606

order
100
455

455
80
family

genus
455
80
node81.members.0.js

7
100
order

family
7
100

genus
node84.members.0.js
96
4

node85.members.0.js
100
3
genus

80
20
order

family
80
20

80
20
node88.members.0.js
genus

80
575
class

order
575
80

family
80
575

80
575
node92.members.0.js
genus

phylum
80
4368

class
80
4368

80
4368
order

4368
80
family

genus
node97.members.0.js
4368
80

phylum
100
58

58
100
class

order
53
100

family
53
100

genus
53
100
node102.members.0.js

order
100
5

family
5
100

genus
node105.members.0.js
100
5

phylum
99.5959
1205

1205
99.488
class

1139
99.5443
order

1072
99.8862
family

node110.members.0.js
184
98.5815
genus

888
99.2523
node111.members.0.js
genus

49
92.9592
family

49
92.9592
node113.members.0.js
genus

family
18
80

node115.members.0.js
18
80
genus

order
98.1961
51

family
98.1961
51

genus
node118.members.0.js
80
46

genus
node119.members.0.js
5
85

85
15
order

15
85
family

node122.members.0.js
85
15
genus

phylum
99.1642
41209

98.6301
1468
class

order
47
90

47
90
family

node127.members.0.js
80
47
genus

order
1232
98.3815

family
8
98

genus
node130.members.0.js
8
98

8
99
family

genus
93
8
node132.members.0.js

family
6
80

genus
node134.members.0.js
6
80

family
94
86

94
80
node136.members.0.js
genus

family
96
1116

genus
1116
96
node138.members.0.js

189
99
order

99
189
family

genus
99
189
node141.members.0.js

class
71
96

71
96
order

family
96
71

node145.members.0.js
96
71
genus

class
6
82

6
82
order

family
82
6

node149.members.0.js
82
6
genus

99.9374
2684
class

99.9374
2684
order

family
99.9374
2684

99.9374
2684
node153.members.0.js
genus

class
98.8246
14769

order
100
12

family
12
100

100
12
node157.members.0.js
genus

28
100
order

family
100
28

28
100
node160.members.0.js
genus

order
99
52

52
99
family

genus
node163.members.0.js
99
52

80
1164
order

family
1164
80

1164
80
node166.members.0.js
genus

order
95.1762
4398

96.5217
23
family

98
8
node169.members.0.js
genus

2
98
node170.members.0.js
genus

genus
95.3846
13
node171.members.0.js

98.5938
32
family

32
98.5938
node173.members.0.js
genus

family
1803
80

genus
1803
80
node175.members.0.js

98.4247
1069
family

genus
69
99.8696
node177.members.0.js

node178.members.0.js
89
10
genus

node179.members.0.js
100
20
genus

node180.members.0.js
108
98
genus

node181.members.0.js
94
25
genus

node182.members.0.js
98
837
genus

family
1241
96.8405

node184.members.0.js
351
89
genus

80
4
node185.members.0.js
genus

100
75
node186.members.0.js
genus

node187.members.0.js
100
811
genus

91
7
family

91
7
node189.members.0.js
genus

family
96.1935
124

genus
95.7903
124
node191.members.0.js

family
99
100

100
99
node193.members.0.js
genus

order
99.9408
9115

family
9111
99.9058

genus
node196.members.0.js
80
63

genus
99.9887
9048
node197.members.0.js

80
4
family

genus
4
80
node199.members.0.js

97.0268
634
class

634
97.0205
order

family
97.0079
634

623
97.0482
node203.members.0.js
genus

genus
11
80
node204.members.0.js

95.9328
16459
class

order
99.8249
7252

family
127
80

genus
80
127
node208.members.0.js

family
1751
100

genus
node210.members.0.js
177
100

node211.members.0.js
1574
96
genus

5374
100
family

5374
100
node213.members.0.js
genus

order
95.7222
18

95.7222
18
family

7
100
node216.members.0.js
genus

11
80
node217.members.0.js
genus

order
155
100

family
100
155

genus
node220.members.0.js
100
155

order
1637
91.0483

91.0483
1637
family

genus
731
100
node223.members.0.js

node224.members.0.js
862
80
genus

genus
node225.members.0.js
99
44

order
90.1862
913

450
80
family

node228.members.0.js
450
80
genus

family
99.9327
208

node230.members.0.js
84
14
genus

96
194
node231.members.0.js
genus

100
2
family

node233.members.0.js
2
100
genus

80.7905
253
family

node235.members.0.js
100
5
genus

100
5
node236.members.0.js
genus

node237.members.0.js
243
80
genus

order
80
3935

family
80
3935

3935
80
node240.members.0.js
genus

2525
98.0277
order

92
2
family

node243.members.0.js
2
91
genus

family
98.2143
56

genus
node245.members.0.js
80
2

genus
100
12
node246.members.0.js

genus
91.6667
12
node247.members.0.js

genus
97
30
node248.members.0.js

43
80
family

80
43
node250.members.0.js
genus

226
100
family

genus
226
100
node252.members.0.js

family
97.7585
2182

genus
93
328
node254.members.0.js

1254
80
node255.members.0.js
genus

node256.members.0.js
600
84.2133
genus

16
100
family

genus
100
16
node258.members.0.js

order
98.5417
24

family
24
98.5417

genus
24
91.8333
node261.members.0.js

class
90
2

2
90
order

2
80
family

node265.members.0.js
2
80
genus

class
4976
80

order
80
4976

family
4976
80

genus
node269.members.0.js
4976
80

class
123
91.2439

123
91.2439
order

65
80
family

genus
node273.members.0.js
65
80

family
87
12

80
12
node275.members.0.js
genus

80
4
family

node277.members.0.js
80
4
genus

family
42
100

genus
31
99
node279.members.0.js

11
80
node280.members.0.js
genus

class
100
17

17
100
order

100
17
family

node284.members.0.js
17
89
genus

phylum
16839
99.3089

class
99.2984
16741

16741
99.2984
order

99.2984
16741
family

node289.members.0.js
80
209
genus

16532
98.9959
node290.members.0.js
genus

class
98
4

order
98
4

family
4
98

node294.members.0.js
98
4
genus

94
99.9574
class

order
99.9574
94

94
99.0851
family

genus
41
80
node298.members.0.js

genus
2
100
node299.members.0.js

genus
node300.members.0.js
22
100

node301.members.0.js
4
99
genus

node302.members.0.js
25
100
genus
