# Supplementary material for: Fungal community profiles in agricultural soils of a long-term field trial under different tillage, fertilization and crop rotation conditions analyzed by high-throughput ITS-amplicon sequencing
Source: PLoS One. 2018 Apr 5;13(4):e0195345. doi: 10.1371/journal.pone.0195345 (PMC5886558; doi:10.1371/journal.pone.0195345)
Supplement: S7 File — (HTML) [file pone.0195345.s017.html]

Javascript must be enabled to view this page.

members
count
unassigned
score
rank

ITS1BC7.fastq\_classified\_otusc\_clean


66549

100
66549
domain

phylum
27895
99.2236

class
885
99.9582

99.9582
order
885

family
885
99.9582

node6.members.0.js
99.9582
genus
885

97.5079
class
2205

order
210
99

99
family
210

node10.members.0.js
210
genus
99

order
1989
97.2207

72
family
80

72
genus
80
node13.members.0.js

96
1683
family

96
genus
1683
node15.members.0.js

family
227
90.5198

genus
180
80
node17.members.0.js

node18.members.0.js
genus
10
96

node19.members.0.js
96
genus
33

4
genus
95
node20.members.0.js

7
family
99

node22.members.0.js
7
genus
93

92
6
order

92
family
6

80
6
genus
node25.members.0.js

98.0996
12717
class

87.601
3391
order

87.601
3391
family

97
genus
948
node29.members.0.js

100
genus
280
node30.members.0.js

node31.members.0.js
genus
2163
80

92.0754
557
order

100
31
family

node34.members.0.js
100
31
genus

80
367
family

80
genus
367
node36.members.0.js

100
159
family

node38.members.0.js
151
genus
97

node39.members.0.js
genus
8
89

99.0439
5695
order

7
family
95

genus
7
93
node42.members.0.js

family
5312
98.8726

node44.members.0.js
genus
1356
96

node45.members.0.js
92.05
140
genus

genus
3577
80
node46.members.0.js

84
genus
18
node47.members.0.js

node48.members.0.js
52
genus
89

169
genus
80
node49.members.0.js

99.9791
191
family

node51.members.0.js
100
187
genus

98
4
genus
node52.members.0.js

100
family
95

genus
39
100
node54.members.0.js

33
genus
100
node55.members.0.js

23
genus
80
node56.members.0.js

100
25
family

node58.members.0.js
100
25
genus

family
65
80

node60.members.0.js
65
genus
80

order
3
100

3
family
88

node63.members.0.js
genus
3
88

1474
order
80

80
family
1474

1474
genus
80
node66.members.0.js

100
3
order

3
family
100

node69.members.0.js
100
genus
3

order
32
99.0625

99.0625
32
family

93.3125
32
genus
node72.members.0.js

19
order
96.2105

96.2105
19
family

node75.members.0.js
100
genus
7

80
genus
12
node76.members.0.js

order
35
100

100
family
35

100
35
genus
node79.members.0.js

99.8979
1508
order

136
family
100

node82.members.0.js
100
genus
136

95
family
2

node84.members.0.js
95
2
genus

80
12
family

genus
12
80
node86.members.0.js

100
1358
family

75
genus
99
node88.members.0.js

node89.members.0.js
97
1283
genus

100
5
class

100
order
5

100
5
family

5
genus
95
node93.members.0.js

90
17
class

90
order
17

family
17
80

node97.members.0.js
80
17
genus

class
411
97.1971

411
order
97.1971

97.1971
411
family

node101.members.0.js
97.1971
411
genus

100
22
class

100
order
22

22
family
100

node105.members.0.js
99
9
genus

88
genus
4
node106.members.0.js

node107.members.0.js
80
genus
9

2850
class
80

2850
order
80

family
2850
80

node111.members.0.js
80
genus
2850

98.8434
8783
class

99.9818
4956
order

4956
family
99.9562

node115.members.0.js
99.0067
4938
genus

18
genus
80
node116.members.0.js

order
493
80

493
family
80

493
genus
80
node119.members.0.js

3334
order
96.5315

67
family
100

node122.members.0.js
100
genus
67

92.2
family
15

genus
3
97
node124.members.0.js

node125.members.0.js
91
genus
12

family
779
99.9859

node127.members.0.js
genus
16
100

genus
3
100
node128.members.0.js

114
genus
100
node129.members.0.js

11
genus
89
node130.members.0.js

node131.members.0.js
98
19
genus

node132.members.0.js
94
genus
41

node133.members.0.js
100
genus
575

95.9524
family
21

node135.members.0.js
95.9524
genus
21

family
981
99.9327

genus
6
89
node137.members.0.js

node138.members.0.js
972
genus
100

node139.members.0.js
99
genus
3

family
8
94

94
8
genus
node141.members.0.js

family
72
97

node143.members.0.js
96
72
genus

1391
family
80

1391
genus
80
node145.members.0.js

80
phylum
3225

80
class
3225

3225
order
80

80
family
3225

genus
3225
80
node150.members.0.js

99.1275
149
phylum

class
147
97.8435

order
106
97.4528

family
5
80

80
genus
5
node155.members.0.js

family
14
99.5

node157.members.0.js
99.5
genus
14

family
87
97.8391

node159.members.0.js
genus
21
98

66
genus
90.8485
node160.members.0.js

96
8
order

family
8
96

node163.members.0.js
96
8
genus

order
28
100

100
28
family

node166.members.0.js
genus
28
94.5357

order
5
80

family
5
80

genus
5
80
node169.members.0.js

80
2
class

80
2
order

2
family
80

genus
2
80
node173.members.0.js

88.9689
phylum
3988

88.6033
1099
class

80
order
671

80
671
family

node178.members.0.js
80
671
genus

100
order
3

family
3
100

node181.members.0.js
genus
3
100

order
78
97.5897

family
45
99.1556

80
4
genus
node184.members.0.js

100
genus
4
node185.members.0.js

genus
6
99
node186.members.0.js

node187.members.0.js
94
31
genus

16
family
99

98
genus
16
node189.members.0.js

7
family
91

node191.members.0.js
91
7
genus

family
5
80

80
5
genus
node193.members.0.js

93
family
5

node195.members.0.js
93
5
genus

90
287
order

287
family
90

node198.members.0.js
genus
287
90

100
order
60

60
family
100

node201.members.0.js
genus
60
100

class
2045
80

80
2045
order

2045
family
80

genus
2045
80
node205.members.0.js

2
class
100

2
order
100

100
2
family

100
2
genus
node209.members.0.js

class
57
100

100
57
order

57
family
100

node213.members.0.js
genus
57
100

701
class
92.0856

95
order
74

family
74
95

node217.members.0.js
95
74
genus

order
6
86.6667

100
family
2

node220.members.0.js
genus
2
100

80
family
4

80
genus
4
node222.members.0.js

416
order
80

416
family
80

416
genus
80
node225.members.0.js

order
35
89.3714

89.3714
35
family

node228.members.0.js
genus
3
89

node229.members.0.js
genus
28
80

node230.members.0.js
4
genus
92

170
order
100

family
165
100

165
genus
100
node233.members.0.js

family
5
80

node235.members.0.js
80
genus
5

class
35
99.7429

order
32
100

32
family
100

node239.members.0.js
32
genus
100

3
order
97

family
3
97

genus
3
97
node242.members.0.js

49
class
100

100
order
45

80
45
family

node246.members.0.js
80
genus
45

order
4
100

100
family
4

4
genus
100
node249.members.0.js

99.0245
31265
phylum

305
class
100

order
305
100

99.8525
family
305

node254.members.0.js
genus
230
100

100
genus
25
node255.members.0.js

node256.members.0.js
genus
45
80

node257.members.0.js
genus
2
100

100
genus
3
node258.members.0.js

class
30956
98.9967

order
30956
98.9967

family
30956
98.9967

genus
1336
80
node262.members.0.js

node263.members.0.js
29620
genus
98.8038

class
2
98

98
2
order

family
2
98

98
2
genus
node267.members.0.js

87
2
class

2
order
87

87
family
2

80
genus
2
node271.members.0.js

100
phylum
27

100
27
class

100
27
order

27
family
100

node276.members.0.js
100
27
genus
