# Supplementary material for: Fungal community profiles in agricultural soils of a long-term field trial under different tillage, fertilization and crop rotation conditions analyzed by high-throughput ITS-amplicon sequencing
Source: PLoS One. 2018 Apr 5;13(4):e0195345. doi: 10.1371/journal.pone.0195345 (PMC5886558; doi:10.1371/journal.pone.0195345)
Supplement: S8 File — (HTML) [file pone.0195345.s018.html]

Javascript must be enabled to view this page.

members
count
unassigned
score
rank

All.fastq\_classified\_otusc\_clean


106374

domain
100
106374

2150
phylum
99.254

2090
class
98.999

91.2308
order
26

6
family
81

genus
81
node6.members.0.js
6

20
94.3
family

20
node8.members.0.js
94.3
genus

51
order
100

100
family
51

node11.members.0.js
51
genus
93.7059

order
98.9237
1965

90
family
80

node14.members.0.js
90
genus
80

family
91.6364
11

node16.members.0.js
11
genus
91.6364

family
99.6636
1864

199
node18.members.0.js
97.196
genus

node19.members.0.js
5
genus
85

node20.members.0.js
1640
genus
99.1591

80
genus
20
node21.members.0.js

80
order
48

80
family
48

48
node24.members.0.js
80
genus

41
96
class

41
96
order

family
96
41

genus
96
node28.members.0.js
41

80
class
19

19
80
order

80
family
19

node32.members.0.js
19
genus
80

51503
99.4215
phylum

90
class
143

143
90
order

143
family
80

genus
80
node37.members.0.js
143

97.3228
class
4098

99
order
5

5
family
99

genus
80
node41.members.0.js
5

order
97.9459
3514

100
family
9

90
genus
9
node44.members.0.js

family
95
3

node46.members.0.js
3
genus
95

family
95.078
205

80
genus
189
node48.members.0.js

genus
96
node49.members.0.js
5

genus
96
node50.members.0.js
11

family
96
3297

genus
96
node52.members.0.js
3297

92
80
order

92
family
80

node55.members.0.js
92
genus
80

487
order
94.0205

family
94.0205
487

94.0205
genus
487
node58.members.0.js

1062
class
97.0885

order
100
7

family
100
7

100
genus
7
node62.members.0.js

11
99
order

99
family
11

genus
99
node65.members.0.js
11

order
97.0374
1044

1044
family
97.0374

node68.members.0.js
6
genus
80

genus
97.0549
node69.members.0.js
1038

class
98.8627
13834

6919
order
97.6724

282
91.0532
family

genus
80
node73.members.0.js
3

279
node74.members.0.js
90.7706
genus

family
100
86

86
node76.members.0.js
100
genus

96.3396
family
53

genus
98
node78.members.0.js
9

genus
98
node79.members.0.js
4

35
node80.members.0.js
95.2
genus

node81.members.0.js
5
genus
100

2188
family
80

80
genus
2188
node83.members.0.js

2871
99.9579
family

95.16
genus
25
node85.members.0.js

2846
node86.members.0.js
100
genus

family
100
447

genus
100
node88.members.0.js
447

980
99.9071
family

58
node90.members.0.js
98
genus

node91.members.0.js
59
genus
100

38
node92.members.0.js
92
genus

100
genus
698
node93.members.0.js

genus
100
node94.members.0.js
36

91
node95.members.0.js
89
genus

12
family
88

genus
88
node97.members.0.js
12

20
order
99

20
99
family

20
node100.members.0.js
99
genus

80
order
1075

1075
80
family

80
genus
1075
node103.members.0.js

99.9896
order
5793

5793
99.9206
family

node106.members.0.js
5778
genus
99.0344

80
genus
15
node107.members.0.js

27
order
100

27
family
100

genus
100
node110.members.0.js
27

98.7316
class
20261

95.351
order
678

315
99.819
family

node114.members.0.js
315
genus
96.2159

family
91.5756
172

161
node116.members.0.js
80
genus

genus
100
node117.members.0.js
11

80
family
191

80
genus
191
node119.members.0.js

763
99.384
order

763
99.384
family

97
genus
470
node122.members.0.js

293
node123.members.0.js
100
genus

100
order
6

family
100
6

100
genus
6
node126.members.0.js

31
95
order

31
95
family

31
node129.members.0.js
80
genus

2599
order
80

2599
family
80

2599
node132.members.0.js
80
genus

56
order
100

56
family
100

56
node135.members.0.js
100
genus

order
97.6277
5498

100
family
1469

100
genus
1469
node138.members.0.js

family
80
63

63
node140.members.0.js
80
genus

2572
100
family

genus
100
node142.members.0.js
339

2233
node143.members.0.js
97
genus

1394
family
87

87
genus
1394
node145.members.0.js

84
order
19

19
84
family

node148.members.0.js
19
genus
84

10534
order
98.9775

9516
family
98.8706

genus
83
node151.members.0.js
289

genus
80
node152.members.0.js
7452

93
genus
214
node153.members.0.js

1561
node154.members.0.js
99
genus

family
93
3

genus
93
node156.members.0.js
3

family
96.9942
173

node158.members.0.js
55
genus
90.5455

genus
82
node159.members.0.js
56

3
node160.members.0.js
80
genus

59
node161.members.0.js
100
genus

100
family
140

node163.members.0.js
140
genus
100

593
family
99.973

genus
100
node165.members.0.js
585

node166.members.0.js
8
genus
96

11
100
family

node168.members.0.js
11
genus
100

family
80
98

genus
80
node170.members.0.js
98

order
97.4595
74

74
97.4595
family

94.125
genus
64
node173.members.0.js

node174.members.0.js
10
genus
80

3
order
100

3
85
family

3
node177.members.0.js
85
genus

31
100
class

order
100
31

31
100
family

31
node181.members.0.js
95
genus

99.6214
class
3164

3164
order
99.6214

3164
99.6214
family

99.6214
genus
3164
node185.members.0.js

2103
99.4883
class

99.4883
order
2103

99.9605
family
2001

80
genus
12
node189.members.0.js

1989
node190.members.0.js
100
genus

32
family
80

80
genus
32
node192.members.0.js

family
88.6857
70

genus
80
node194.members.0.js
70

class
80
6807

80
order
6807

80
family
6807

genus
80
node198.members.0.js
6807

6738
94.6425
phylum

544
class
80

544
order
80

family
80
544

genus
80
node203.members.0.js
544

class
92.111
3929

order
89.8726
2237

99.4515
family
536

99
genus
81
node207.members.0.js

node208.members.0.js
231
genus
94.4675

224
node209.members.0.js
94.1607
genus

family
100
30

node211.members.0.js
30
genus
99

40
83.8
family

83.8
genus
40
node213.members.0.js

1608
80
family

1608
node215.members.0.js
80
genus

family
91
7

7
node217.members.0.js
80
genus

100
family
7

genus
99
node219.members.0.js
7

4
family
100

node221.members.0.js
4
genus
100

family
90
5

90
genus
5
node223.members.0.js

89
order
2

2
89
family

87
genus
2
node226.members.0.js

967
80
order

80
family
967

967
node229.members.0.js
80
genus

6
100
order

family
100
6

100
genus
6
node232.members.0.js

18
96.3333
order

18
96.3333
family

node235.members.0.js
6
genus
89

node236.members.0.js
12
genus
100

order
97.9693
684

663
family
98

663
node239.members.0.js
98
genus

21
97
family

21
node241.members.0.js
94
genus

15
order
100

family
80
15

80
genus
15
node244.members.0.js

96.0149
class
1947

136
100
order

family
100
136

100
genus
136
node248.members.0.js

order
80
378

378
family
80

node251.members.0.js
378
genus
80

order
96.2047
1075

100
family
871

871
node254.members.0.js
100
genus

80
family
204

204
node256.members.0.js
80
genus

57
order
99.386

53
99.3396
family

node259.members.0.js
5
genus
80

node260.members.0.js
48
genus
100

99
family
4

node262.members.0.js
4
genus
99

301
95.5482
order

301
95.5482
family

80
genus
150
node265.members.0.js

97.6794
genus
131
node266.members.0.js

90
genus
20
node267.members.0.js

class
100
6

6
order
100

family
100
6

100
genus
6
node271.members.0.js

59
100
class

100
order
54

54
100
family

genus
100
node275.members.0.js
54

5
order
100

5
100
family

genus
100
node278.members.0.js
5

253
class
96.0909

212
order
96.0377

156
80
family

genus
80
node282.members.0.js
156

56
85
family

node284.members.0.js
56
genus
85

order
80
30

30
80
family

30
node287.members.0.js
80
genus

100
order
11

11
100
family

11
node290.members.0.js
100
genus

phylum
97.617
188

182
98.1978
class

80
order
18

18
80
family

18
node295.members.0.js
80
genus

164
99.5366
order

99.5366
family
164

99.5366
genus
164
node298.members.0.js

6
class
80

6
order
80

80
family
6

node302.members.0.js
6
genus
80

98.9373
phylum
40448

434
100
class

434
100
order

family
99.2396
434

4
node307.members.0.js
94
genus

node308.members.0.js
153
genus
80

genus
100
node309.members.0.js
115

genus
100
node310.members.0.js
162

98.9253
class
39974

98.9253
order
39974

39974
family
98.9253

genus
98.5064
node314.members.0.js
39374

node315.members.0.js
600
genus
80

class
99.425
40

99.425
order
40

40
family
99.425

99.425
genus
40
node319.members.0.js

5347
80
phylum

80
class
5347

order
80
5347

5347
80
family

genus
80
node324.members.0.js
5347
