# Supplementary material for: Fungal community profiles in agricultural soils of a long-term field trial under different tillage, fertilization and crop rotation conditions analyzed by high-throughput ITS-amplicon sequencing
Source: PLoS One. 2018 Apr 5;13(4):e0195345. doi: 10.1371/journal.pone.0195345 (PMC5886558; doi:10.1371/journal.pone.0195345)
Supplement: S9 File — (HTML) [file pone.0195345.s019.html]

Javascript must be enabled to view this page.

members
count
unassigned
score
rank

ITS2BC8.fastq\_final.fastq\_classified\_otusc\_clean


53098

100
53098
domain

132
phylum
100

100
100
class

100
order
100

100
100
family

87.141
node6.members.0.js
genus
78

80
node7.members.0.js
genus
22

100
class
32

32
order
100

32
family
100

83
node11.members.0.js
16
genus

100
node12.members.0.js
6
genus

genus
10
node13.members.0.js
100

49206
phylum
99.8377

99.2815
20495
class

98.5559
11308
order

7772
family
80

7772
genus
node18.members.0.js
80

2612
family
93.3078

80
node20.members.0.js
genus
1127

882
genus
85.9184
node21.members.0.js

genus
603
node22.members.0.js
97.9138

99.8052
924
family

genus
332
80
node24.members.0.js

node25.members.0.js
94.1098
genus
592

1250
order
99.9976

99.9907
965
family

3
genus
node28.members.0.js
96

genus
962
node29.members.0.js
100

100
family
281

281
genus
90
node31.members.0.js

82
4
family

82
node33.members.0.js
genus
4

1158
order
80

80
1158
family

1158
genus
80
node36.members.0.js

5522
order
98.9643

80
111
family

111
genus
80
node39.members.0.js

220
family
95.9

220
genus
95.9
node41.members.0.js

2
family
100

node43.members.0.js
100
genus
2

4425
family
98.468

node45.members.0.js
98.5557
2048
genus

genus
1747
80
node46.members.0.js

90
node47.members.0.js
genus
5

node48.members.0.js
91.7075
genus
400

genus
225
node49.members.0.js
80.1778

100
189
family

node51.members.0.js
100
genus
5

node52.members.0.js
93
genus
33

node53.members.0.js
95.3709
genus
151

124
family
100

100
node55.members.0.js
genus
124

100
264
family

node57.members.0.js
100
genus
243

node58.members.0.js
100
genus
21

187
family
97.1604

genus
147
node60.members.0.js
97.8027

genus
40
80
node61.members.0.js

95.5
16
order

family
16
95.5

genus
6
node64.members.0.js
83

10
genus
node65.members.0.js
80

134
order
100

134
family
96

134
genus
96
node68.members.0.js

274
order
100

274
family
100

node71.members.0.js
100
genus
274

99.7338
541
order

99.7338
541
family

100
node74.members.0.js
7
genus

genus
54
node75.members.0.js
99.6296

95
node76.members.0.js
genus
472

80
node77.members.0.js
genus
8

292
order
97.7055

99.2652
264
family

264
genus
node80.members.0.js
80

80
28
family

80
node82.members.0.js
genus
28

29
class
100

29
order
100

100
family
29

genus
9
node86.members.0.js
80

20
genus
94.9
node87.members.0.js

1250
class
93

93
1250
order

1250
family
93

93
node91.members.0.js
1250
genus

8
class
84

84
8
order

84
8
family

8
genus
node95.members.0.js
84

15512
class
99.2214

99.5454
5037
order

96.1084
775
family

node99.members.0.js
87.5
genus
150

625
genus
95.968
node100.members.0.js

80
2440
family

genus
2440
80
node102.members.0.js

5
family
100

node104.members.0.js
100
genus
5

99.827
1474
family

5
genus
node106.members.0.js
81

100
node107.members.0.js
genus
17

node108.members.0.js
80
genus
51

genus
971
97
node109.members.0.js

genus
430
node110.members.0.js
99

295
family
89

genus
295
node112.members.0.js
89

99.8333
48
family

99.8333
node114.members.0.js
genus
48

5
order
100

100
5
family

node117.members.0.js
100
genus
5

940
order
80

940
family
80

node120.members.0.js
80
genus
940

9465
order
100

9465
family
100

genus
9465
100
node123.members.0.js

81
8
order

8
family
81

genus
8
node126.members.0.js
81

25
order
88.6

88.6
25
family

genus
10
node129.members.0.js
99

genus
15
node130.members.0.js
81

100
32
order

100
32
family

genus
32
node133.members.0.js
100

1139
class
92.4864

99.3333
6
order

6
family
99.3333

2
genus
node137.members.0.js
94

node138.members.0.js
80
genus
4

80
2
order

80
2
family

genus
2
node141.members.0.js
80

1072
order
92.041

215
family
80

80
node144.members.0.js
genus
215

family
12
81.6667

genus
4
node146.members.0.js
83

8
genus
80
node147.members.0.js

93
806
family

806
genus
node149.members.0.js
93

39
family
100

genus
39
100
node151.members.0.js

59
order
99.5932

100
49
family

node154.members.0.js
100
49
genus

96.8
10
family

genus
2
node156.members.0.js
80

node157.members.0.js
95
genus
8

1655
class
98.5166

98.5166
1655
order

98.5166
1655
family

genus
1655
98.5166
node161.members.0.js

98.6466
5789
class

98.8726
4607
order

80
133
family

80
node165.members.0.js
genus
133

282
family
96.3936

node167.members.0.js
98
genus
3

95.5072
node168.members.0.js
276
genus

node169.members.0.js
80
genus
3

9
family
100

node171.members.0.js
100
genus
9

99.9524
3783
family

99.9524
node173.members.0.js
genus
3783

90.5
family
400

80
node175.members.0.js
256
genus

genus
144
node176.members.0.js
88

8
order
80

8
family
80

node179.members.0.js
80
8
genus

order
1174
94.5852

1174
family
94.5852

94.5852
node182.members.0.js
genus
1174

4
class
86

4
order
86

4
family
86

genus
4
node186.members.0.js
86

3099
class
80

80
3099
order

80
3099
family

genus
3099
node190.members.0.js
80

99.2345
226
class

99.2345
226
order

174
family
98.9828

genus
174
80
node194.members.0.js

100
7
family

100
node196.members.0.js
genus
7

45
family
99.6444

45
genus
node198.members.0.js
99.6444

99.4924
2878
phylum

33
class
100

100
33
order

33
family
100

node203.members.0.js
100
33
genus

100
36
class

36
order
100

family
36
100

100
node207.members.0.js
genus
36

74
class
99.6216

7
order
80

family
7
80

node211.members.0.js
80
genus
7

67
order
95.9701

80
48
family

genus
48
node214.members.0.js
80

19
family
100

node216.members.0.js
100
genus
19

1854
class
99.7174

order
49
80

80
49
family

80
node220.members.0.js
genus
49

99.9223
193
order

190
family
100

100
node223.members.0.js
genus
190

95
3
family

genus
3
node225.members.0.js
95

99.3125
336
order

100
family
53

2
genus
node228.members.0.js
100

genus
51
node229.members.0.js
100

family
262
100

node231.members.0.js
80
genus
2

genus
8
node232.members.0.js
100

genus
246
node233.members.0.js
100

node234.members.0.js
100
genus
6

80
18
family

node236.members.0.js
80
genus
18

3
family
99

node238.members.0.js
97
genus
3

85
4
order

4
family
84

4
genus
node241.members.0.js
80

100
12
order

5
family
100

genus
5
100
node244.members.0.js

7
family
80

80
node246.members.0.js
genus
7

89
order
7

7
family
80

genus
7
80
node249.members.0.js

100
1253
order

100
1253
family

genus
1253
100
node252.members.0.js

class
14
97

14
order
80

14
family
80

14
genus
node256.members.0.js
80

43
class
80

80
43
order

43
family
80

genus
43
node260.members.0.js
80

824
class
97.2828

56
order
83.2143

9
family
100

9
genus
100
node264.members.0.js

80
47
family

node266.members.0.js
80
genus
47

99.2
20
order

20
family
99.2

node269.members.0.js
99.2
20
genus

172
order
80

80
172
family

genus
172
node272.members.0.js
80

96.7606
564
order

96.7606
family
564

95.5443
node275.members.0.js
genus
564

100
12
order

12
family
100

genus
12
100
node278.members.0.js

110
phylum
100

100
110
class

8
order
100

8
family
100

genus
8
100
node283.members.0.js

39
order
100

100
39
family

node286.members.0.js
100
genus
39

55
order
100

55
family
100

55
genus
100
node289.members.0.js

order
8
100

8
family
100

genus
8
node292.members.0.js
100

80
593
phylum

593
class
80

593
order
80

593
family
80

node297.members.0.js
80
genus
593

100
179
phylum

class
179
100

179
order
100

100
family
179

node302.members.0.js
100
genus
179
