# Supplementary material for: Fungal community profiles in agricultural soils of a long-term field trial under different tillage, fertilization and crop rotation conditions analyzed by high-throughput ITS-amplicon sequencing
Source: PLoS One. 2018 Apr 5;13(4):e0195345. doi: 10.1371/journal.pone.0195345 (PMC5886558; doi:10.1371/journal.pone.0195345)
Supplement: S10 File — (HTML) [file pone.0195345.s020.html]

Javascript must be enabled to view this page.

members
count
unassigned
score
rank

All.fastq\_classified\_otusc\_clean


80089

domain
80089
100

98.4495
396
phylum

class
98.4495
396

order
98.3081
396

family
396
98.3081

99.4944
356
node6.members.0.js
genus

80
genus
40
node7.members.0.js

99.7512
71197
phylum

98.8915
13591
class

order
2588
95.0502

2588
95.0502
family

95.0502
2588
node12.members.0.js
genus

order
99.6504
10933

80
125
family

node15.members.0.js
125
genus
80

91.6129
310
family

genus
node17.members.0.js
280
80

genus
node18.members.0.js
30
88

family
99.9535
9798

genus
9798
node20.members.0.js
99.9535

700
96.3757
family

95.5268
genus
691
node22.members.0.js

80
genus
9
node23.members.0.js

68
80
order

family
80
68

genus
node26.members.0.js
68
80

100
2
order

100
2
family

100
node29.members.0.js
2
genus

class
99.1949
5269

order
5269
99.1949

family
99.1949
5269

99.1949
node33.members.0.js
5269
genus

class
89
14

order
14
89

89
14
family

89
node37.members.0.js
14
genus

class
1558
92

order
92
1558

family
1558
92

92
node41.members.0.js
1558
genus

class
91.534
2603

94.0833
12
order

2
86
family

2
node45.members.0.js
genus
86

3
100
family

100
genus
node47.members.0.js
3

80
3
family

3
node49.members.0.js
genus
80

4
81
family

node51.members.0.js
4
genus
80

order
91.48
2581

86.4247
186
family

100
genus
3
node54.members.0.js

80
119
node55.members.0.js
genus

83
64
node56.members.0.js
genus

family
93
2013

93
genus
2013
node58.members.0.js

80
382
family

genus
node60.members.0.js
382
80

10
100
order

family
100
10

80
10
node63.members.0.js
genus

class
23965
98.1801

80
943
order

80
943
family

genus
943
node67.members.0.js
80

order
95.45
20

family
95.45
20

genus
node70.members.0.js
20
80

order
590
100

family
100
590

91
node73.members.0.js
genus
99.033

32
node74.members.0.js
genus
100

95
467
node75.members.0.js
genus

order
98.2311
6492

86.703
101
family

86.703
101
node78.members.0.js
genus

family
451
100

80
genus
164
node80.members.0.js

node81.members.0.js
5
genus
100

genus
14
node82.members.0.js
93

node83.members.0.js
268
genus
94.097

100
28
family

100
node85.members.0.js
28
genus

4815
97.3551
family

node87.members.0.js
2160
genus
97.6352

91.4273
node88.members.0.js
791
genus

node89.members.0.js
1864
genus
80

family
87
80

80
genus
node91.members.0.js
87

201
99.6567
family

201
node93.members.0.js
genus
97.8259

family
100
809

759
node95.members.0.js
genus
100

100
node96.members.0.js
50
genus

order
99.9405
1327

96
100
family

genus
node99.members.0.js
4
100

genus
node100.members.0.js
92
96

family
99.8075
1231

genus
1152
node102.members.0.js
100

genus
79
node103.members.0.js
96

427
99.2248
order

427
99.2248
family

427
node106.members.0.js
genus
80

order
97.4282
13986

80
2794
family

80
2794
node109.members.0.js
genus

family
100
5

genus
node111.members.0.js
5
80

89
216
family

89
node113.members.0.js
216
genus

family
89.9588
9500

node115.members.0.js
5522
genus
80

96
genus
596
node116.members.0.js

node117.members.0.js
491
genus
97.8432

1023
node118.members.0.js
genus
88.8612

84
genus
1868
node119.members.0.js

family
1471
99.6111

node121.members.0.js
604
genus
89.7748

80
node122.members.0.js
867
genus

2
100
order

family
2
100

genus
node125.members.0.js
2
100

100
2
order

100
2
family

genus
2
node128.members.0.js
100

order
100
58

family
58
96

58
node131.members.0.js
genus
96

100
118
order

100
118
family

100
node134.members.0.js
118
genus

7
80
class

order
7
80

7
80
family

node138.members.0.js
7
genus
80

class
1703
99.4439

order
1703
99.4439

family
669
100

genus
669
node142.members.0.js
100

633
96.9068
family

89.8182
77
node144.members.0.js
genus

80
556
node145.members.0.js
genus

family
99.9776
401

80
genus
node147.members.0.js
6

genus
node148.members.0.js
395
98.2785

class
5301
80

5301
80
order

family
80
5301

80
genus
node152.members.0.js
5301

class
99.2424
66

66
99.2424
order

99.2424
66
family

96.8889
genus
9
node156.members.0.js

100
7
node157.members.0.js
genus

80
genus
node158.members.0.js
50

98.8688
17120
class

80
1635
order

1635
80
family

80
1635
node162.members.0.js
genus

9089
99.5605
order

1534
99.9296
family

99
node165.members.0.js
714
genus

100
genus
11
node166.members.0.js

98
7
node167.members.0.js
genus

775
node168.members.0.js
genus
97

node169.members.0.js
27
genus
80

family
80
4515

node171.members.0.js
4515
genus
80

family
462
91

node173.members.0.js
462
genus
91

93
3
family

80
genus
node175.members.0.js
3

99
97.5051
family

node177.members.0.js
99
genus
97.5051

family
2177
100

100
node179.members.0.js
2177
genus

299
98.4548
family

93.9692
node181.members.0.js
260
genus

94.8718
genus
node182.members.0.js
39

order
100
124

family
100
124

100
genus
node185.members.0.js
124

order
59
100

100
59
family

genus
node188.members.0.js
59
100

order
100
6174

family
6174
100

node191.members.0.js
6174
genus
100

order
98.8205
39

98.8205
39
family

83
2
node194.members.0.js
genus

genus
node195.members.0.js
6
96

100
genus
31
node196.members.0.js

99.7342
158
phylum

43
100
class

43
100
order

100
43
family

genus
18
node201.members.0.js
100

100
genus
9
node202.members.0.js

83
genus
node203.members.0.js
12

genus
4
node204.members.0.js
100

115
99.6087
class

order
99.6087
115

99.6087
115
family

100
node208.members.0.js
45
genus

80
genus
node209.members.0.js
70

1144
99.8322
phylum

class
99.8254
1134

99.8951
286
order

29
98.6207
family

node214.members.0.js
5
genus
80

genus
node215.members.0.js
24
100

100
257
family

genus
257
node217.members.0.js
100

order
100
10

family
10
100

genus
node220.members.0.js
10
100

99.4478
833
order

family
92.3684
95

92.3684
genus
95
node223.members.0.js

family
99.9837
738

80
node225.members.0.js
4
genus

223
node226.members.0.js
genus
99.5471

99.9374
node227.members.0.js
511
genus

order
100
5

family
5
100

5
node230.members.0.js
genus
100

class
96
10

order
96
10

family
10
96

genus
10
node234.members.0.js
96

99.2028
4650
phylum

192
99.5156
class

3
83
order

family
83
3

node239.members.0.js
3
genus
80

order
97.7931
174

family
80
102

80
genus
node242.members.0.js
102

family
97.375
72

genus
72
node244.members.0.js
97.375

order
80
15

80
15
family

genus
node247.members.0.js
15
80

1719
97.6731
class

11
90
order

11
90
family

11
node251.members.0.js
genus
86

order
100
26

100
26
family

genus
node254.members.0.js
26
100

85.8571
42
order

family
80
42

genus
node257.members.0.js
42
80

order
96.983
824

98.2474
97
family

68
node260.members.0.js
genus
100

94.1379
29
node261.members.0.js
genus

100
551
family

100
223
node263.members.0.js
genus

node264.members.0.js
268
genus
100

96
genus
node265.members.0.js
3

100
node266.members.0.js
57
genus

99
30
family

node268.members.0.js
30
genus
99

80
146
family

node270.members.0.js
146
genus
80

order
80
100

family
100
80

80
node273.members.0.js
genus
100

order
100
11

family
7
100

100
genus
7
node276.members.0.js

family
4
100

100
genus
node278.members.0.js
4

order
324
100

family
100
13

genus
13
node281.members.0.js
80

311
100
family

100
node283.members.0.js
311
genus

order
99.5625
64

47
100
family

node286.members.0.js
47
genus
100

80
17
family

genus
17
node288.members.0.js
80

80
337
order

80
337
family

genus
337
node291.members.0.js
80

class
200
80

80
200
order

family
200
80

node295.members.0.js
200
genus
80

class
483
96.1698

order
92.0729
480

480
92.0729
family

89
445
node299.members.0.js
genus

node300.members.0.js
35
genus
80

3
100
order

family
100
3

100
3
node303.members.0.js
genus

class
100
71

order
100
71

71
100
family

100
genus
71
node307.members.0.js

100
55
class

order
100
55

family
55
100

55
node311.members.0.js
genus
100

99
3
class

order
3
99

99
3
family

3
node315.members.0.js
genus
98

class
99.2636
1927

order
20
100

family
100
20

100
genus
20
node319.members.0.js

order
98.6739
1374

family
1374
98.6739

node322.members.0.js
1361
genus
97.5092

80
13
node323.members.0.js
genus

97.6667
21
order

93
7
family

genus
node326.members.0.js
7
93

family
14
100

node328.members.0.js
10
genus
100

node329.members.0.js
4
genus
100

80
424
order

family
80
424

80
genus
node332.members.0.js
424

100
88
order

family
88
98.8068

100
node335.members.0.js
81
genus

7
node336.members.0.js
genus
85

phylum
80
2544

2544
80
class

order
2544
80

family
80
2544

2544
node341.members.0.js
genus
80
