# Supplementary material for: Fungal community profiles in agricultural soils of a long-term field trial under different tillage, fertilization and crop rotation conditions analyzed by high-throughput ITS-amplicon sequencing
Source: PLoS One. 2018 Apr 5;13(4):e0195345. doi: 10.1371/journal.pone.0195345 (PMC5886558; doi:10.1371/journal.pone.0195345)
Supplement: S11 File — (HTML) [file pone.0195345.s021.html]

Javascript must be enabled to view this page.

members
count
unassigned
score
rank

All.fastq\_classified\_otusc\_clean


78723

78723
100
domain

1453
80
phylum

80
1453
class

order
1453
80

80
1453
family

1453
node6.members.0.js
80
genus

264
98.9015
phylum

98.6932
264
class

27
98.8889
order

98.8889
27
family

genus
98.8889
node11.members.0.js
27

order
204
98

family
58
92.1207

92.1207
genus
58
node14.members.0.js

family
146
99.5411

genus
99.1507
node16.members.0.js
146

100
33
order

family
100
3

genus
100
node19.members.0.js
3

100
30
family

genus
100
node21.members.0.js
30

7
100
phylum

7
100
class

100
7
order

100
7
family

node26.members.0.js
7
genus
100

phylum
100
255

class
124
100

124
100
order

family
124
100

node31.members.0.js
25
genus
100

node32.members.0.js
5
genus
100

node33.members.0.js
84
genus
83.8095

10
node34.members.0.js
100
genus

100
131
class

100
131
order

131
100
family

80
genus
101
node38.members.0.js

node39.members.0.js
30
genus
100

65953
99.7747
phylum

class
100
20

order
100
20

family
100
20

genus
100
node44.members.0.js
8

90
genus
12
node45.members.0.js

9334
98.2204
class

order
80
128

family
128
80

128
node49.members.0.js
80
genus

order
98.8149
7743

80
68
family

80
genus
68
node52.members.0.js

family
514
94.3813

514
node54.members.0.js
93.2743
genus

family
858
87.4091

genus
80
node56.members.0.js
663

82
genus
195
node57.members.0.js

6298
99.9594
family

genus
99.9594
node59.members.0.js
6298

family
5
98

5
node61.members.0.js
98
genus

92.1757
1463
order

92.1757
1463
family

node64.members.0.js
1463
genus
92.1757

class
1762
93

93
1762
order

1762
93
family

genus
93
node68.members.0.js
1762

28304
99.299
class

order
100
5

100
5
family

genus
100
node72.members.0.js
5

80
689
order

80
689
family

689
node75.members.0.js
80
genus

order
1936
99.9933

family
687
100

687
node78.members.0.js
96
genus

family
99.9686
1244

genus
100
node80.members.0.js
1218

96
genus
13
node81.members.0.js

node82.members.0.js
13
genus
80

family
88
5

88
genus
5
node84.members.0.js

order
348
100

348
96
family

genus
96
node87.members.0.js
348

98.5242
910
order

family
910
98.5242

node90.members.0.js
906
genus
80

genus
100
node91.members.0.js
4

order
1024
100

family
100
1024

114
node94.members.0.js
99.3158
genus

96
genus
898
node95.members.0.js

12
node96.members.0.js
100
genus

98.7889
13706
order

99.7745
1419
family

genus
80
node99.members.0.js
667

93.2593
genus
752
node100.members.0.js

family
5
100

80
genus
5
node102.members.0.js

family
98.1971
3115

988
node104.members.0.js
95.5243
genus

912
node105.members.0.js
80
genus

genus
85.8881
node106.members.0.js
1215

9167
80
family

genus
80
node108.members.0.js
9167

18
93.7222
order

family
93.7222
18

node111.members.0.js
13
genus
80

83
genus
5
node112.members.0.js

order
99.2443
9377

family
89.5855
193

genus
89.5855
node115.members.0.js
193

99.947
434
family

99.2581
genus
434
node117.members.0.js

99.0271
8203
family

2782
node119.members.0.js
80
genus

2
node120.members.0.js
100
genus

91.0163
genus
675
node121.members.0.js

4740
node122.members.0.js
99.4819
genus

4
node123.members.0.js
90
genus

100
45
family

100
genus
45
node125.members.0.js

family
100
381

genus
100
node127.members.0.js
352

100
genus
29
node128.members.0.js

family
87
100

genus
100
node130.members.0.js
10

93
genus
22
node131.members.0.js

28
node132.members.0.js
98
genus

80
genus
27
node133.members.0.js

80
34
family

genus
80
node135.members.0.js
34

order
100
291

family
291
100

node138.members.0.js
291
genus
100

class
86
5

order
5
86

family
86
5

5
node142.members.0.js
86
genus

class
1665
92.1153

2
100
order

family
100
2

97
genus
2
node146.members.0.js

1650
92.0436
order

family
13
100

genus
100
node149.members.0.js
13

family
80
180

node151.members.0.js
180
genus
80

family
1432
93

genus
93
node153.members.0.js
1432

25
83
family

83
genus
25
node155.members.0.js

order
100
13

family
10
100

10
node158.members.0.js
100
genus

family
3
100

80
genus
3
node160.members.0.js

100
7
class

100
7
order

7
80
family

7
node164.members.0.js
80
genus

class
80
8

order
8
80

80
8
family

80
genus
8
node168.members.0.js

class
89
4

4
89
order

family
4
89

genus
89
node172.members.0.js
4

98.6352
2352
class

98.6352
2352
order

98.6352
2352
family

genus
98.6352
node176.members.0.js
2352

class
2
84

order
2
84

family
84
2

84
genus
2
node180.members.0.js

16473
98.8649
class

order
54
98.9444

family
54
98.9444

node184.members.0.js
3
genus
81

genus
99
node185.members.0.js
6

genus
100
node186.members.0.js
45

100
59
order

family
59
100

genus
100
node189.members.0.js
59

order
5
100

5
100
family

100
genus
5
node192.members.0.js

order
80
1180

1180
80
family

80
genus
1180
node195.members.0.js

order
100
7046

7046
100
family

100
genus
7046
node198.members.0.js

order
12
100

family
100
12

node201.members.0.js
12
genus
100

99.5509
8117
order

family
4
91

node204.members.0.js
4
genus
91

family
97.5728
2041

node206.members.0.js
5
genus
95

node207.members.0.js
23
genus
80

95.9074
genus
1943
node208.members.0.js

70
node209.members.0.js
88.8571
genus

2589
99.9305
family

node211.members.0.js
36
genus
80

99
genus
1499
node212.members.0.js

24
node213.members.0.js
100
genus

node214.members.0.js
25
genus
81

20
node215.members.0.js
100
genus

97
genus
985
node216.members.0.js

family
3306
80

node218.members.0.js
3306
genus
80

92
99.8261
family

99.7391
genus
92
node220.members.0.js

29
98.0345
family

3
node222.members.0.js
80
genus

node223.members.0.js
26
genus
100

family
91.1277
47

genus
91.1277
node225.members.0.js
47

family
9
97

87
genus
9
node227.members.0.js

class
80
5249

order
80
5249

family
80
5249

genus
80
node231.members.0.js
5249

class
99.5964
768

order
768
99.5964

373
99.9678
family

369
node235.members.0.js
95.1816
genus

80
genus
4
node236.members.0.js

299
99.1472
family

node238.members.0.js
6
genus
81

node239.members.0.js
293
genus
80

family
99.0521
96

node241.members.0.js
96
genus
99.0521

10791
99.8808
phylum

class
99.7742
186

96.6667
180
order

family
149
80

80
genus
149
node246.members.0.js

family
100
31

100
genus
31
node248.members.0.js

6
80
order

family
80
6

80
genus
6
node251.members.0.js

97.8791
2787
class

order
45
100

45
100
family

genus
100
node255.members.0.js
45

order
127
84.0945

101
80
family

genus
80
node258.members.0.js
101

26
100
family

100
genus
26
node260.members.0.js

5
99.2
order

93.2
5
family

2
node263.members.0.js
80
genus

genus
100
node264.members.0.js
3

order
335
80

335
80
family

335
node267.members.0.js
80
genus

order
97.4857
2275

family
97.4857
2275

2273
node270.members.0.js
96.8104
genus

2
node271.members.0.js
80
genus

class
36
80

order
80
36

family
80
36

genus
80
node275.members.0.js
36

98
5
class

order
80
5

80
5
family

genus
80
node279.members.0.js
5

99.7786
7474
class

16
100
order

10
80
family

node283.members.0.js
10
genus
80

family
100
6

100
genus
6
node285.members.0.js

93.3544
6580
order

family
100
3

genus
100
node288.members.0.js
3

2593
80
family

80
genus
2593
node290.members.0.js

family
98.0764
3872

genus
100
node292.members.0.js
113

3759
node293.members.0.js
98.0186
genus

family
8
99

node295.members.0.js
8
genus
99

100
77
family

6
node297.members.0.js
100
genus

node298.members.0.js
9
genus
100

genus
100
node299.members.0.js
62

family
27
100

node301.members.0.js
27
genus
99

order
13
100

100
13
family

13
node304.members.0.js
100
genus

54
90
order

family
80
54

80
genus
54
node307.members.0.js

85.6667
18
order

80
18
family

node310.members.0.js
18
genus
80

265
100
order

family
265
100

genus
100
node313.members.0.js
265

order
528
80

family
80
528

80
genus
528
node316.members.0.js

class
100
53

100
53
order

53
100
family

node320.members.0.js
53
genus
100

96
36
class

order
92
36

family
92
36

node324.members.0.js
36
genus
89

class
100
214

order
100
214

100
214
family

genus
100
node328.members.0.js
214
