# Supplementary material for: Fungal community profiles in agricultural soils of a long-term field trial under different tillage, fertilization and crop rotation conditions analyzed by high-throughput ITS-amplicon sequencing
Source: PLoS One. 2018 Apr 5;13(4):e0195345. doi: 10.1371/journal.pone.0195345 (PMC5886558; doi:10.1371/journal.pone.0195345)
Supplement: S12 File — (HTML) [file pone.0195345.s022.html]

Javascript must be enabled to view this page.

members
count
unassigned
score
rank

ITS2BC11.fastq\_final.fastq\_classified\_otusc\_clean


50737

100
domain
50737

100
phylum
199

class
100
175

175
100
order

175
family
100

80
node6.members.0.js
genus
141

node7.members.0.js
100
34
genus

24
100
class

24
100
order

24
100
family

100
node11.members.0.js
genus
9

93.8667
node12.members.0.js
genus
15

45927
99.572
phylum

12221
class
98.3626

100
order
17

100
family
17

genus
17
100
node17.members.0.js

4926
order
99.4572

146
89
family

89
node20.members.0.js
genus
146

97
family
364

87
node22.members.0.js
genus
364

family
100
71

genus
71
100
node24.members.0.js

80
family
2367

2367
genus
node26.members.0.js
80

14
family
91

14
genus
node28.members.0.js
91

family
99.8043
1610

genus
21
100
node30.members.0.js

4
genus
node31.members.0.js
81

422
genus
node32.members.0.js
99

84
genus
node33.members.0.js
80

1023
genus
node34.members.0.js
97

node35.members.0.js
100
56
genus

253
95.7589
family

138
genus
node37.members.0.js
95.6739

80
node38.members.0.js
genus
52

90
node39.members.0.js
genus
63

101
86.0396
family

86.0396
node41.members.0.js
genus
101

order
99.3333
21

21
99.3333
family

node44.members.0.js
96
7
genus

99
node45.members.0.js
genus
14

order
100
5117

family
100
5117

94
node48.members.0.js
genus
2

100
node49.members.0.js
genus
5115

order
99.5
4

4
99.5
family

node52.members.0.js
99.5
4
genus

46
order
100

family
100
46

node55.members.0.js
100
46
genus

order
80
2090

2090
80
family

genus
2090
80
node58.members.0.js

100
class
19

order
100
19

19
100
family

genus
8
92.5
node62.members.0.js

100
node63.members.0.js
genus
11

80
class
4199

80
order
4199

4199
80
family

4199
genus
node67.members.0.js
80

7496
99.1597
class

order
99.2052
6354

92.2105
family
1848

genus
1460
80
node71.members.0.js

243
genus
node72.members.0.js
83

145
genus
node73.members.0.js
89.6552

family
99.9956
4121

genus
4121
99.9956
node75.members.0.js

family
80
49

genus
49
80
node77.members.0.js

100
family
30

30
genus
node79.members.0.js
85

family
97.3856
306

306
genus
node81.members.0.js
96.4052

92.8345
order
1142

1142
family
92.8345

92.8345
node84.members.0.js
genus
1142

1849
class
95.8269

1849
order
95.8269

200
family
99.095

99.095
node88.members.0.js
genus
200

93.9909
family
1431

17
genus
node90.members.0.js
81

61
genus
node91.members.0.js
80

80
node92.members.0.js
genus
1344

genus
9
100
node93.members.0.js

218
100
family

99.9048
node95.members.0.js
genus
210

80
node96.members.0.js
genus
8

1737
class
97.4093

1737
order
97.4093

1737
97.4093
family

97.4093
node100.members.0.js
genus
1737

1019
class
92.6997

995
92.5528
order

78
family
80

node104.members.0.js
80
78
genus

788
family
93

93
node106.members.0.js
genus
788

family
91.6418
67

3
genus
node108.members.0.js
80

46
genus
node109.members.0.js
91

node110.members.0.js
83
18
genus

family
100
62

100
node112.members.0.js
genus
62

97.25
order
12

97.25
family
12

genus
4
80
node115.members.0.js

genus
3
89
node116.members.0.js

97
node117.members.0.js
genus
5

order
99
12

family
100
3

3
genus
node120.members.0.js
99

family
80
6

80
node122.members.0.js
genus
6

3
family
100

100
node124.members.0.js
genus
3

class
80
6

80
order
6

6
family
80

6
genus
node128.members.0.js
80

98.2508
class
16548

1629
order
80

80
family
1629

node132.members.0.js
80
1629
genus

6
97
order

6
97
family

node135.members.0.js
97
6
genus

100
order
107

107
family
100

107
genus
node138.members.0.js
100

863
order
99.9873

5
family
93

5
genus
node141.members.0.js
80

809
family
99.9852

96
node143.members.0.js
genus
4

genus
805
100
node144.members.0.js

family
100
47

90
node146.members.0.js
genus
47

2
family
82

genus
2
82
node148.members.0.js

order
99.9253
937

family
99.9253
937

genus
30
100
node151.members.0.js

genus
71
98.6479
node152.members.0.js

node153.members.0.js
95
831
genus

5
genus
node154.members.0.js
80

order
99.4331
635

635
family
99.4331

node157.members.0.js
80
632
genus

genus
3
100
node158.members.0.js

order
100
59

family
96
59

node161.members.0.js
96
59
genus

99.2227
order
6750

100
family
186

40
genus
node164.members.0.js
100

146
genus
node165.members.0.js
100

family
87.6541
159

genus
159
87.6541
node167.members.0.js

5134
98.8448
family

1768
genus
node169.members.0.js
98.7817

253
genus
node170.members.0.js
80.5692

node171.members.0.js
100
4
genus

node172.members.0.js
80
2586
genus

523
genus
node173.members.0.js
91.7094

family
99.9196
336

genus
336
99.4911
node175.members.0.js

72
family
100

72
genus
node177.members.0.js
100

100
family
800

5
genus
node179.members.0.js
100

genus
148
94.4122
node180.members.0.js

genus
586
80
node181.members.0.js

genus
61
87
node182.members.0.js

63
family
80

63
genus
node184.members.0.js
80

99.64
order
50

50
family
99.64

genus
46
94.2609
node187.members.0.js

genus
4
80
node188.members.0.js

100
order
4

100
family
4

node191.members.0.js
100
4
genus

order
97.3076
5508

99.7441
family
1184

517
genus
node194.members.0.js
92.2031

node195.members.0.js
80
667
genus

2123
family
94.4936

90.9485
node197.members.0.js
genus
447

80
node198.members.0.js
genus
934

86.8194
node199.members.0.js
genus
742

100
family
5

genus
5
80
node201.members.0.js

family
80
2196

80
node203.members.0.js
genus
2196

93
class
831

831
order
93

831
93
family

genus
831
93
node207.members.0.js

86
class
2

order
86
2

86
family
2

2
genus
node211.members.0.js
86

phylum
99.6545
301

301
99.6312
class

301
99.588
order

family
90.9167
24

24
genus
node216.members.0.js
90.9167

277
family
99.9747

genus
136
99.9338
node218.members.0.js

100
node219.members.0.js
genus
141

phylum
80
843

843
class
80

843
order
80

843
family
80

node224.members.0.js
80
843
genus

3450
99.5023
phylum

1944
99.6816
class

order
80
208

80
family
208

genus
208
80
node229.members.0.js

24
100
order

100
family
24

node232.members.0.js
100
24
genus

order
99.5192
1585

family
96.0407
1377

node235.members.0.js
96.0407
1377
genus

34
family
96

96
node237.members.0.js
genus
34

80
family
45

80
node239.members.0.js
genus
45

100
family
23

node241.members.0.js
100
23
genus

81
100
family

100
node243.members.0.js
genus
36

10
genus
node244.members.0.js
100

genus
35
99.8
node245.members.0.js

93
family
25

genus
4
97
node247.members.0.js

genus
21
87.8571
node248.members.0.js

22
order
100

80
family
4

genus
4
80
node251.members.0.js

family
100
18

85
node253.members.0.js
genus
18

100
order
98

100
family
11

11
genus
node256.members.0.js
95

100
family
87

87
genus
node258.members.0.js
100

100
order
5

100
family
5

genus
5
100
node261.members.0.js

100
order
2

2
family
100

genus
2
100
node264.members.0.js

class
99.8473
131

98.5565
order
124

family
80
103

103
genus
node268.members.0.js
80

21
97.9048
family

21
genus
node270.members.0.js
97.9048

100
order
2

2
100
family

100
node273.members.0.js
genus
2

5
80
order

family
80
5

80
node276.members.0.js
genus
5

11
99.0909
class

11
98.1818
order

family
98.1818
11

98.1818
node280.members.0.js
genus
11

9
97
class

9
order
92

92
family
9

genus
9
89
node284.members.0.js

class
97.4444
9

80
order
7

7
family
80

node288.members.0.js
80
7
genus

2
order
99

2
family
99

genus
2
98
node291.members.0.js

class
80
14

order
80
14

family
80
14

14
genus
node295.members.0.js
80

100
class
158

order
100
158

family
100
158

genus
158
100
node299.members.0.js

class
97.7411
1174

100
order
21

family
100
21

21
genus
node303.members.0.js
100

182
order
80

182
family
80

182
genus
node306.members.0.js
80

96.9933
order
898

96.9933
family
898

96.0135
node309.members.0.js
genus
891

genus
7
80
node310.members.0.js

90.4478
order
67

32
family
80

genus
32
80
node313.members.0.js

35
family
100

node315.members.0.js
100
2
genus

genus
33
100
node316.members.0.js

6
order
98.6667

family
98
6

node319.members.0.js
92
2
genus

node320.members.0.js
100
4
genus

93.4118
phylum
17

17
93.4118
class

93
order
17

17
93
family

node325.members.0.js
99.4
10
genus

80
node326.members.0.js
genus
7
