# Supplementary material for: Fungal community profiles in agricultural soils of a long-term field trial under different tillage, fertilization and crop rotation conditions analyzed by high-throughput ITS-amplicon sequencing
Source: PLoS One. 2018 Apr 5;13(4):e0195345. doi: 10.1371/journal.pone.0195345 (PMC5886558; doi:10.1371/journal.pone.0195345)
Supplement: S13 File — (HTML) [file pone.0195345.s023.html]

Javascript must be enabled to view this page.

members
count
unassigned
score
rank

ITS2BC5.fastq\_final.fastq\_classified\_otusc\_clean


48193

domain
100
48193

phylum
100
112

100
class
64

order
100
64

100
family
64

8
genus
node6.members.0.js
83

genus
100
node7.members.0.js
42

node8.members.0.js
100
genus
10

4
genus
100
node9.members.0.js

48
class
100

48
100
order

48
100
family

11
100
node13.members.0.js
genus

genus
node14.members.0.js
80
37

phylum
99.8405
44795

7
80
class

7
order
80

family
80
7

genus
80
node19.members.0.js
7

1261
class
92.391

7
100
order

100
family
7

80
node23.members.0.js
genus
7

order
96.2857
7

100
family
5

5
genus
100
node26.members.0.js

80
family
2

genus
80
node28.members.0.js
2

92.3039
order
1247

12
family
80

genus
node31.members.0.js
80
12

98
82.4694
family

80
node33.members.0.js
genus
48

50
83
node34.members.0.js
genus

93
family
1109

genus
node36.members.0.js
93
1109

99
family
28

28
genus
node38.members.0.js
99

11624
99.2526
class

99.9817
order
1417

1378
99.9434
family

92.5778
node42.members.0.js
genus
180

1198
genus
100
node43.members.0.js

37
family
100

37
genus
90
node45.members.0.js

2
family
82

82
node47.members.0.js
genus
2

297
99.202
order

297
99.202
family

80
node50.members.0.js
genus
297

order
98.1831
3331

family
93.3409
132

132
93.3409
node53.members.0.js
genus

142
family
100

16
93
node55.members.0.js
genus

5
genus
100
node56.members.0.js

121
genus
92.4959
node57.members.0.js

52
family
100

52
genus
100
node59.members.0.js

74
family
98.2973

genus
node61.members.0.js
81
6

genus
node62.members.0.js
98.3235
68

180
100
family

genus
node64.members.0.js
100
174

6
100
node65.members.0.js
genus

family
98.3711
2385

224
genus
80
node67.members.0.js

5
node68.members.0.js
80
genus

genus
node69.members.0.js
80
740

genus
node70.members.0.js
98.5586
1271

node71.members.0.js
91.6414
genus
145

366
80
family

366
node73.members.0.js
80
genus

216
order
100

family
100
216

genus
node76.members.0.js
100
216

14
100
order

96
family
14

node79.members.0.js
96
genus
14

80
order
461

461
family
80

461
node82.members.0.js
80
genus

5090
99.2972
order

family
98.4792
2623

1820
genus
node85.members.0.js
86.15

genus
node86.members.0.js
98.5886
525

100
node87.members.0.js
genus
2

274
genus
node88.members.0.js
80

node89.members.0.js
96
genus
2

1364
family
99.9883

488
node91.members.0.js
97.3197
genus

genus
80
node92.members.0.js
876

80
family
1103

1103
80
node94.members.0.js
genus

100
order
3

3
family
100

genus
91
node97.members.0.js
3

order
100
774

774
100
family

38
genus
99.4211
node100.members.0.js

704
95
node101.members.0.js
genus

100
node102.members.0.js
genus
32

21
95.0952
order

21
family
95.0952

genus
80
node105.members.0.js
12

node106.members.0.js
86.7778
genus
9

18205
class
98.7038

100
order
7

100
family
7

genus
node110.members.0.js
99
7

7503
100
order

7503
family
100

7503
node113.members.0.js
100
genus

100
order
29

family
100
29

29
100
node116.members.0.js
genus

569
order
80

569
80
family

genus
80
node119.members.0.js
569

6
order
100

6
100
family

genus
node122.members.0.js
100
6

order
97.8733
10091

family
99.6879
769

5
node125.members.0.js
100
genus

48
node126.members.0.js
80
genus

97
node127.members.0.js
genus
632

3
genus
node128.members.0.js
81

81
genus
99
node129.members.0.js

34
family
100

34
genus
node131.members.0.js
100

3454
80
family

genus
80
node133.members.0.js
3454

family
100
5

5
genus
80
node135.members.0.js

99.2727
family
33

33
genus
99.2727
node137.members.0.js

92.08
family
25

genus
node139.members.0.js
92.08
25

5771
family
95.8534

4923
95.4739
node141.members.0.js
genus

genus
node142.members.0.js
85.4175
848

class
94.4763
8369

89.3451
order
7024

family
80.4375
3785

3647
genus
node146.members.0.js
80

138
genus
80
node147.members.0.js

100
family
6

6
80
node149.members.0.js
genus

2725
family
99.9681

2725
99.9681
node151.members.0.js
genus

411
96.0998
family

356
genus
node153.members.0.js
95.8876

55
genus
80
node154.members.0.js

55
100
family

genus
100
node156.members.0.js
55

42
80
family

node158.members.0.js
80
genus
42

1345
95.1048
order

1345
95.1048
family

95.1048
node161.members.0.js
genus
1345

3
86
class

86
order
3

3
86
family

3
86
node165.members.0.js
genus

100
class
11

11
order
100

11
100
family

genus
100
node169.members.0.js
3

5
node170.members.0.js
80
genus

3
genus
node171.members.0.js
99

80
class
1959

80
order
1959

family
80
1959

80
node175.members.0.js
genus
1959

1811
class
98.2165

order
98.2165
1811

family
98.2165
1811

1811
98.2165
node179.members.0.js
genus

1090
class
98.9248

order
98.9248
1090

217
96.4562
family

2
node183.members.0.js
81
genus

81
node184.members.0.js
genus
142

node185.members.0.js
80
genus
73

family
99.9897
388

305
100
node187.members.0.js
genus

80
node188.members.0.js
genus
83

485
family
99.0454

node190.members.0.js
99.0454
genus
485

455
93
class

455
order
93

455
family
93

455
93
node194.members.0.js
genus

100
phylum
107

class
99.9439
107

107
99.9439
order

107
99.9439
family

50
80
node199.members.0.js
genus

node200.members.0.js
100
genus
31

genus
node201.members.0.js
92.5769
26

78
phylum
100

78
class
100

100
order
78

100
family
78

100
node206.members.0.js
genus
78

98.7926
phylum
2469

class
80
65

80
order
65

65
80
family

genus
80
node211.members.0.js
65

1063
96.096
class

31
99.6129
order

family
86
3

genus
node215.members.0.js
86
3

100
family
28

28
node217.members.0.js
100
genus

order
94.6264
886

family
94.6264
886

genus
93.8883
node220.members.0.js
886

8
order
100

family
100
8

8
genus
100
node223.members.0.js

order
80
95

80
family
95

95
genus
80
node226.members.0.js

43
order
98.1163

23
family
97

23
97
node229.members.0.js
genus

99.1
family
20

92
node231.members.0.js
genus
3

17
genus
node232.members.0.js
100

class
100
3

order
100
3

100
family
3

3
100
node236.members.0.js
genus

class
97
109

order
92
109

109
family
92

109
node240.members.0.js
89
genus

98.7664
class
214

30
85.6333
order

85.6333
family
30

30
node244.members.0.js
80
genus

184
99.7554
order

family
100
74

74
100
node247.members.0.js
genus

110
family
80

110
80
node249.members.0.js
genus

27
100
class

27
100
order

27
100
family

100
node253.members.0.js
genus
27

914
class
98.7549

423
99.9622
order

96
family
4

4
node257.members.0.js
80
genus

419
family
100

419
100
node259.members.0.js
genus

order
80
244

244
80
family

genus
node262.members.0.js
80
244

order
85
32

32
family
84

32
node265.members.0.js
80
genus

order
95.7578
161

72
97
family

100
node268.members.0.js
genus
17

55
genus
95.7455
node269.members.0.js

100
family
2

genus
100
node271.members.0.js
2

48
80
family

genus
80
node273.members.0.js
48

100
family
34

34
genus
node275.members.0.js
99.1471

100
family
2

genus
node277.members.0.js
100
2

3
93
family

3
genus
80
node279.members.0.js

100
order
3

3
family
100

100
node282.members.0.js
genus
3

order
100
18

family
100
18

18
genus
100
node285.members.0.js

18
89
order

18
family
80

node288.members.0.js
80
genus
18

100
order
15

10
family
100

node291.members.0.js
100
genus
10

family
80
5

5
genus
80
node293.members.0.js

class
100
74

100
order
74

74
100
family

node297.members.0.js
100
genus
74

80
phylum
632

632
class
80

632
order
80

632
family
80

632
genus
node302.members.0.js
80
