# Supplementary material for: Fungal community profiles in agricultural soils of a long-term field trial under different tillage, fertilization and crop rotation conditions analyzed by high-throughput ITS-amplicon sequencing
Source: PLoS One. 2018 Apr 5;13(4):e0195345. doi: 10.1371/journal.pone.0195345 (PMC5886558; doi:10.1371/journal.pone.0195345)
Supplement: S14 File — (HTML) [file pone.0195345.s024.html]

Javascript must be enabled to view this page.

members
count
unassigned
score
rank

ITS2BC12.fastq\_final.fastq\_classified\_otusc\_clean


93340

93340
domain
100

phylum
80
1750

1750
class
80

order
80
1750

1750
80
family

1750
node6.members.0.js
genus
80

12
100
phylum

12
class
100

12
100
order

12
100
family

100
12
genus
node11.members.0.js

phylum
99.8026
304

class
99.8026
304

97.8452
order
84

31
93.0968
family

genus
node16.members.0.js
31
93.0968

100
family
53

100
8
node18.members.0.js
genus

100
node19.members.0.js
genus
45

100
order
210

100
family
14

98.0714
node22.members.0.js
genus
14

196
100
family

genus
node24.members.0.js
196
100

10
100
order

10
family
100

100
genus
node27.members.0.js
10

83477
phylum
99.9198

3331
class
91.4173

17
order
97.3529

100
family
7

7
genus
node32.members.0.js
100

6
family
80

80
node34.members.0.js
genus
6

4
family
100

80
2
genus
node36.members.0.js

100
genus
node37.members.0.js
2

98.4286
order
14

14
98.4286
family

89
2
genus
node40.members.0.js

12
node41.members.0.js
genus
80

order
91.3488
3300

216
80
family

80
216
node44.members.0.js
genus

93
family
2677

2677
node46.members.0.js
genus
93

family
83.4816
407

83
341
genus
node48.members.0.js

44
genus
node49.members.0.js
80

91.9091
node50.members.0.js
genus
22

948
class
93

948
order
93

family
93
948

93
genus
node54.members.0.js
948

90
class
2

2
order
90

90
family
2

node58.members.0.js
genus
2
90

class
80
13

13
order
80

13
80
family

13
node62.members.0.js
genus
80

59
class
100

59
order
100

family
100
59

100
8
genus
node66.members.0.js

genus
node67.members.0.js
33
91.1515

18
genus
node68.members.0.js
80

class
99.2229
26337

29
100
order

29
family
100

100
node72.members.0.js
genus
29

692
80
order

family
80
692

node75.members.0.js
genus
692
80

452
100
order

100
family
452

100
452
node78.members.0.js
genus

order
100
13801

13801
100
family

node81.members.0.js
genus
13801
100

98.7912
order
11321

89.8199
family
161

node84.members.0.js
genus
161
89.8199

99.5458
family
3479

24
node86.members.0.js
genus
85.0833

100
genus
node87.members.0.js
17

99
1226
node88.members.0.js
genus

node89.members.0.js
genus
316
80

100
node90.members.0.js
genus
145

97
1751
node91.members.0.js
genus

4054
80
family

4054
node93.members.0.js
genus
80

91
100
family

91
node95.members.0.js
genus
100

39
100
family

80
39
genus
node97.members.0.js

99.8
family
20

99.8
20
node99.members.0.js
genus

family
97.0345
3477

3144
genus
node101.members.0.js
95.9793

87.1381
genus
node102.members.0.js
333

42
order
94.5714

42
family
94.5714

12
genus
node105.members.0.js
81

8
genus
node106.members.0.js
80

22
genus
node107.members.0.js
99

86
class
14

14
86
order

86
family
14

genus
node111.members.0.js
14
86

96.7569
class
3398

order
96.7569
3398

96.7569
family
3398

3398
node115.members.0.js
genus
96.7569

class
98.2503
7890

1888
order
94.6324

94.6324
family
1888

1888
genus
node119.members.0.js
94.6324

97.3294
order
5907

88.5387
family
1240

88
1077
genus
node122.members.0.js

163
genus
node123.members.0.js
80

3691
family
99.8385

3691
node125.members.0.js
genus
99.8385

50
family
80

50
node127.members.0.js
genus
80

97.9092
family
914

80
genus
node129.members.0.js
5

96.9417
node130.members.0.js
genus
909

100
family
12

80
node132.members.0.js
genus
12

order
80
95

family
80
95

node135.members.0.js
genus
95
80

5346
class
99.7909

order
99.7909
5346

4402
family
100

99.9586
node139.members.0.js
genus
4400

node140.members.0.js
genus
2
80

198
96.8889
family

80
130
node142.members.0.js
genus

100
3
genus
node143.members.0.js

82
65
genus
node144.members.0.js

746
99.2735
family

99.2735
746
genus
node146.members.0.js

21
class
99.5238

21
99.5238
order

21
99.5238
family

99.5238
21
node150.members.0.js
genus

class
80
4597

80
order
4597

80
family
4597

node154.members.0.js
genus
4597
80

31521
99.4031
class

542
100
order

family
100
542

100
542
genus
node158.members.0.js

10028
97.9215
order

2471
90.049
family

genus
node161.members.0.js
1798
80

338
genus
node162.members.0.js
88.858

100
335
genus
node163.members.0.js

100
family
22

22
node165.members.0.js
genus
80

65
89
family

65
node167.members.0.js
genus
89

5597
family
80

80
genus
node169.members.0.js
5597

1873
99.7934
family

80
node171.members.0.js
genus
489

1384
genus
node172.members.0.js
97.2724

14093
99.9915
order

2134
99.8355
family

node175.members.0.js
genus
59
80

100
1958
genus
node176.members.0.js

96
genus
node177.members.0.js
117

3
family
82

82
genus
node179.members.0.js
3

100
family
11956

2
genus
node181.members.0.js
80

11954
genus
node182.members.0.js
98

7
order
100

7
family
100

7
genus
node185.members.0.js
100

10
100
order

96
family
10

node188.members.0.js
genus
10
96

4636
98.9422
order

7
family
100

100
7
genus
node191.members.0.js

80
family
127

80
node193.members.0.js
genus
127

216
100
family

100
11
node195.members.0.js
genus

node196.members.0.js
genus
181
96.2541

93
24
node197.members.0.js
genus

99.9396
family
298

298
node199.members.0.js
genus
99.6174

3558
family
98.6518

90.3848
460
genus
node201.members.0.js

100
node202.members.0.js
genus
2

80
984
genus
node203.members.0.js

97.8654
1872
node204.members.0.js
genus

240
node205.members.0.js
genus
80

family
91.2714
70

91.2714
70
genus
node207.members.0.js

360
family
100

100
node209.members.0.js
genus
102

100
genus
node210.members.0.js
258

584
98.4897
order

29
family
80

80
29
node213.members.0.js
genus

99.4559
family
555

555
node215.members.0.js
genus
80

585
order
80

80
family
585

585
genus
node218.members.0.js
80

51
order
95.4902

51
95.4902
family

34
genus
node221.members.0.js
80

86.7647
17
node222.members.0.js
genus

order
99.8438
922

922
99.8438
family

genus
node225.members.0.js
8
80

37
genus
node226.members.0.js
100

96
811
genus
node227.members.0.js

99.6364
genus
node228.members.0.js
66

63
order
99.8095

63
family
99.8095

12
node231.members.0.js
genus
99

80
2
genus
node232.members.0.js

91
49
genus
node233.members.0.js

7685
phylum
98.3188

95.5302
class
1624

9
order
100

family
100
9

100
9
genus
node238.members.0.js

order
100
251

236
family
100

genus
node241.members.0.js
236
100

100
family
15

15
genus
node243.members.0.js
80

2
100
order

2
family
100

node246.members.0.js
genus
2
100

order
99.8667
15

4
family
100

100
node249.members.0.js
genus
4

9
80
family

80
9
node251.members.0.js
genus

2
family
83

80
node253.members.0.js
genus
2

58
order
87.1034

58
family
87.1034

44
genus
node256.members.0.js
83

100
14
genus
node257.members.0.js

95.4444
order
18

14
80
family

80
genus
node260.members.0.js
14

4
80
family

genus
node262.members.0.js
4
80

order
80
617

617
80
family

80
node265.members.0.js
genus
617

90.7061
order
643

80
family
97.6

94.9268
node268.members.0.js
genus
41

39
node269.members.0.js
genus
100

418
family
80

418
genus
node271.members.0.js
80

99.9528
family
127

5
genus
node273.members.0.js
100

100
node274.members.0.js
genus
24

100
node275.members.0.js
genus
95

3
genus
node276.members.0.js
98

family
100
4

100
4
node278.members.0.js
genus

100
family
14

14
genus
node280.members.0.js
100

89.8182
order
11

11
family
89.4545

6
genus
node283.members.0.js
80

92
5
genus
node284.members.0.js

class
80
357

80
order
357

family
80
357

node288.members.0.js
genus
357
80

10
class
98.5

10
order
97

10
97
family

10
genus
node292.members.0.js
97

57
100
class

57
100
order

family
100
57

100
node296.members.0.js
genus
57

class
98.2381
21

17
93.6471
order

17
family
93.6471

80
node300.members.0.js
genus
14

node301.members.0.js
genus
3
89

100
order
4

100
family
4

node304.members.0.js
genus
4
100

1136
class
98.4498

order
89.0632
190

190
89.0632
family

node308.members.0.js
genus
190
80

941
95.2944
order

89.7922
family
255

89.7922
node311.members.0.js
genus
255

666
80
family

80
genus
node313.members.0.js
666

family
85
20

85
node315.members.0.js
genus
20

5
order
80

5
family
80

genus
node318.members.0.js
5
80

4480
class
96.6217

order
80
192

family
80
192

genus
node322.members.0.js
192
80

3846
order
95.2871

family
95.2871
3846

34
node325.members.0.js
genus
80

94.8227
genus
node326.members.0.js
3812

203
99.7044
order

family
99.5567
203

160
node329.members.0.js
genus
100

genus
node330.members.0.js
43
97.2093

53
order
100

53
family
100

100
53
node333.members.0.js
genus

186
99.3978
order

152
100
family

100
152
genus
node336.members.0.js

family
88.4706
34

88.4706
genus
node338.members.0.js
34

112
100
phylum

74
class
100

100
order
74

74
family
100

100
16
node343.members.0.js
genus

83
node344.members.0.js
genus
26

100
genus
node345.members.0.js
24

100
genus
node346.members.0.js
8

38
class
100

38
100
order

100
family
38

80
genus
node350.members.0.js
16

22
node351.members.0.js
genus
100
