# Supplementary material for: Fungal community profiles in agricultural soils of a long-term field trial under different tillage, fertilization and crop rotation conditions analyzed by high-throughput ITS-amplicon sequencing
Source: PLoS One. 2018 Apr 5;13(4):e0195345. doi: 10.1371/journal.pone.0195345 (PMC5886558; doi:10.1371/journal.pone.0195345)
Supplement: S15 File — (HTML) [file pone.0195345.s025.html]

Javascript must be enabled to view this page.

members
count
unassigned
score
rank

All.fastq\_classified\_otusc\_clean


94148

94148
100
domain

3
85
phylum

85
class
3

3
85
order

family
85
3

genus
80
3
node6.members.0.js

phylum
99.727
6769

class
80
35

35
order
80

80
family
35

35
genus
80
node11.members.0.js

190
class
100

190
100
order

190
100
family

node15.members.0.js
genus
100
190

class
100
184

96.5598
order
184

80
family
151

node19.members.0.js
genus
80
151

99.0303
family
33

node21.members.0.js
99.0303
genus
33

99.4212
class
3694

300
80
order

300
family
80

node25.members.0.js
300
80
genus

order
100
2453

100
family
2442

node28.members.0.js
2442
genus
100

family
80
11

80
genus
11
node30.members.0.js

order
100
64

64
family
80

node33.members.0.js
genus
80
64

256
order
99.6172

71
family
97.0423

node36.members.0.js
100
genus
15

node37.members.0.js
3
genus
100

95.7358
genus
53
node38.members.0.js

family
100
4

4
100
genus
node40.members.0.js

family
99.9763
169

67
99.8806
genus
node42.members.0.js

node43.members.0.js
16
genus
100

80
genus
42
node44.members.0.js

node45.members.0.js
33
100
genus

2
genus
96
node46.members.0.js

genus
99.1111
9
node47.members.0.js

family
100
12

12
100
genus
node49.members.0.js

133
order
90

family
80
133

133
80
genus
node52.members.0.js

488
99.1434
order

209
98
family

node55.members.0.js
209
80
genus

279
100
family

279
genus
100
node57.members.0.js

class
97
5

5
order
92

5
92
family

node61.members.0.js
genus
89
5

2624
class
98.1185

1987
98.1943
order

1987
98.1943
family

genus
97.1374
1987
node65.members.0.js

80
order
274

274
80
family

node68.members.0.js
274
80
genus

30
order
100

30
family
100

genus
100
30
node71.members.0.js

134
98.0597
order

2
family
99

genus
99
2
node74.members.0.js

132
family
83.3864

node76.members.0.js
129
genus
80

node77.members.0.js
genus
100
3

199
order
86.7337

99.0625
family
64

60
genus
100
node80.members.0.js

node81.members.0.js
4
genus
85

3
family
100

node83.members.0.js
genus
100
3

family
80
132

132
80
genus
node85.members.0.js

37
100
class

order
100
37

100
family
37

37
100
genus
node89.members.0.js

phylum
99.4267
83266

34336
99.1035
class

2631
order
99.9913

88
family
6

node94.members.0.js
6
88
genus

2358
99.9707
family

node96.members.0.js
genus
100
2215

92.6434
genus
143
node97.members.0.js

267
100
family

node99.members.0.js
267
96
genus

121
100
order

121
family
100

node102.members.0.js
121
100
genus

12086
order
97.2203

family
99.9375
3807

node105.members.0.js
2902
98.47
genus

905
80
genus
node106.members.0.js

family
90.9342
3965

945
genus
98.5577
node108.members.0.js

80
genus
2598
node109.members.0.js

153
genus
94
node110.members.0.js

87.5019
genus
269
node111.members.0.js

family
80
4289

4289
genus
80
node113.members.0.js

100
family
25

80
genus
25
node115.members.0.js

80
100
order

80
family
96

node118.members.0.js
genus
96
80

97.2474
order
1055

family
97.2474
1055

1055
80
genus
node121.members.0.js

400
order
80

family
80
400

400
80
genus
node124.members.0.js

95.3368
order
95

95
family
95.3368

9
genus
99
node127.members.0.js

86
genus
80
node128.members.0.js

99.2963
order
13746

385
99.626
family

93
genus
91
node131.members.0.js

54
genus
97.3333
node132.members.0.js

193
genus
95.2953
node133.members.0.js

47
genus
80
node134.members.0.js

88.088
family
284

node136.members.0.js
88.088
genus
284

1076
family
99.7853

node138.members.0.js
1059
99.6959
genus

node139.members.0.js
80
genus
13

4
92
genus
node140.members.0.js

family
80
91

node142.members.0.js
91
genus
80

100
family
378

node144.members.0.js
329
100
genus

genus
100
49
node145.members.0.js

184
family
100

node147.members.0.js
184
100
genus

98.8028
family
11348

80
genus
5179
node149.members.0.js

node150.members.0.js
4949
genus
99.0721

100
genus
3
node151.members.0.js

8
genus
88
node152.members.0.js

node153.members.0.js
2
genus
100

88.3314
genus
1207
node154.members.0.js

14
100
order

family
100
14

node157.members.0.js
genus
100
14

order
99.9328
4108

99.9328
family
4108

299
98.9498
genus
node160.members.0.js

node161.members.0.js
genus
96
2474

node162.members.0.js
18
genus
80

node163.members.0.js
genus
100
1317

99.1675
class
25218

24
100
order

24
100
family

100
genus
24
node167.members.0.js

1297
80
order

family
80
1297

80
genus
1297
node170.members.0.js

10330
99.3729
order

54
97.8148
family

node173.members.0.js
32
87
genus

node174.members.0.js
80
genus
22

107
family
89

89
genus
107
node176.members.0.js

1628
99.656
family

4
85
genus
node178.members.0.js

node179.members.0.js
137
100
genus

node180.members.0.js
1266
genus
98

node181.members.0.js
68
genus
99

node182.members.0.js
140
genus
80

genus
100
13
node183.members.0.js

3467
family
97.3718

node185.members.0.js
genus
91.83
100

node186.members.0.js
3367
genus
95.5174

260
92.5462
family

node188.members.0.js
92.5462
genus
260

4775
family
80

80
genus
4775
node190.members.0.js

39
99
family

node192.members.0.js
39
genus
99

13534
100
order

13534
family
100

node195.members.0.js
13534
100
genus

33
order
95.3939

33
95.3939
family

21
genus
100
node198.members.0.js

genus
99
4
node199.members.0.js

genus
81
8
node200.members.0.js

8
80
class

8
80
order

80
family
8

8
80
genus
node204.members.0.js

80
class
6740

order
80
6740

6740
family
80

node208.members.0.js
6740
genus
80

100
class
4

4
order
100

4
100
family

100
genus
4
node212.members.0.js

class
98.4385
10821

8229
98.6947
order

181
family
80

node216.members.0.js
genus
80
181

family
91.158
753

node218.members.0.js
genus
80
570

node219.members.0.js
genus
88
169

14
genus
80
node220.members.0.js

6107
99.9761
family

node222.members.0.js
6107
genus
99.9761

family
100
6

6
genus
80
node224.members.0.js

92.247
family
1182

80
genus
62
node226.members.0.js

1120
genus
91.3714
node227.members.0.js

2583
order
94.0774

2583
family
94.0774

node230.members.0.js
94.0774
genus
2583

order
80
9

80
family
9

node233.members.0.js
9
genus
80

29
100
class

100
order
29

100
family
29

node237.members.0.js
22
97.6364
genus

genus
80
7
node238.members.0.js

768
class
99.1419

768
order
99.1419

99.4677
family
62

genus
95.9259
54
node242.members.0.js

node243.members.0.js
80
genus
8

99.0635
family
567

genus
99.0635
567
node245.members.0.js

99.3165
family
139

100
genus
3
node247.members.0.js

genus
80
110
node248.members.0.js

genus
81
26
node249.members.0.js

1115
93
class

1115
93
order

1115
93
family

node253.members.0.js
1115
genus
93

class
92.5886
2273

15
order
93.9333

6
100
family

node257.members.0.js
genus
100
6

4
80
family

node259.members.0.js
4
genus
80

5
92.2
family

genus
92.2
5
node261.members.0.js

order
92.5025
2239

family
93
2094

node264.members.0.js
93
genus
2094

family
83.9375
64

node266.members.0.js
83
genus
49

node267.members.0.js
80
genus
15

80
family
81

node269.members.0.js
81
genus
80

19
100
order

family
100
19

80
genus
12
node272.members.0.js

node273.members.0.js
genus
96
7

84
class
3

3
84
order

family
84
3

node277.members.0.js
genus
84
3

1951
class
97.7883

order
97.7883
1951

1951
97.7883
family

node281.members.0.js
1951
genus
97.7883

3540
phylum
80

3540
class
80

80
order
3540

family
80
3540

3540
80
genus
node286.members.0.js

99.6923
phylum
39

class
99.6923
39

39
99.6923
order

100
family
37

node291.members.0.js
genus
100
37

family
94
2

node293.members.0.js
genus
94
2

531
phylum
100

276
class
100

order
100
276

100
family
276

node298.members.0.js
45
100
genus

node299.members.0.js
79
genus
84.5063

node300.members.0.js
100
genus
100

node301.members.0.js
52
100
genus

class
100
255

order
100
255

family
100
255

78
100
genus
node305.members.0.js

node306.members.0.js
177
genus
80
