# Supplementary material for: Fungal community profiles in agricultural soils of a long-term field trial under different tillage, fertilization and crop rotation conditions analyzed by high-throughput ITS-amplicon sequencing
Source: PLoS One. 2018 Apr 5;13(4):e0195345. doi: 10.1371/journal.pone.0195345 (PMC5886558; doi:10.1371/journal.pone.0195345)
Supplement: S16 File — (HTML) [file pone.0195345.s026.html]

Javascript must be enabled to view this page.

members
count
unassigned
score
rank

ITS2BC10.fastq\_final.fastq\_classified\_otusc\_clean


60537

60537
100
domain

phylum
1027
80

class
1027
80

80
1027
order

family
1027
80

node6.members.0.js
1027
genus
80

100
207
phylum

100
38
class

order
38
100

100
38
family

genus
100
node11.members.0.js
24

14
node12.members.0.js
80
genus

169
100
class

order
169
100

169
100
family

genus
100
node16.members.0.js
25

85
node17.members.0.js
100
genus

node18.members.0.js
4
genus
100

genus
83
node19.members.0.js
55

phylum
8616
99.6743

99.6333
120
class

order
98.5083
120

family
100
12

12
node24.members.0.js
100
genus

family
4
85

4
node26.members.0.js
85
genus

family
104
80

node28.members.0.js
104
genus
80

class
100
25

order
100
25

100
25
family

genus
100
node32.members.0.js
25

class
100
20

100
20
order

20
100
family

100
genus
20
node36.members.0.js

99.5937
6930
class

13
100
order

2
91
family

genus
91
node40.members.0.js
2

11
80
family

11
node42.members.0.js
80
genus

order
6319
97.3406

family
1191
99.1033

100
genus
322
node45.members.0.js

97.0333
genus
360
node46.members.0.js

509
node47.members.0.js
99.0491
genus

family
4923
96.9622

node49.members.0.js
4923
genus
96.9547

family
100
13

100
genus
13
node51.members.0.js

96.8889
9
family

genus
96
node53.members.0.js
7

node54.members.0.js
2
genus
100

family
4
100

genus
99
node56.members.0.js
4

158
80
family

genus
80
node58.members.0.js
158

family
98.8095
21

node60.members.0.js
11
genus
97

node61.members.0.js
10
genus
97

order
100
16

100
16
family

16
node64.members.0.js
100
genus

241
99.8838
order

35
99.2
family

14
node67.members.0.js
80
genus

genus
96.5238
node68.members.0.js
21

family
206
100

206
node70.members.0.js
100
genus

80
288
order

family
80
288

288
node73.members.0.js
80
genus

53
85.3019
order

family
53
80

80
genus
53
node76.members.0.js

class
40
80

80
40
order

family
80
40

80
genus
40
node80.members.0.js

class
16
97

16
92
order

92
16
family

node84.members.0.js
16
genus
89

class
1465
97.6778

order
22
100

family
100
22

node88.members.0.js
22
genus
100

order
1154
96.8726

1154
96.8726
family

95.9921
genus
1145
node91.members.0.js

genus
80
node92.members.0.js
9

80
146
order

family
146
80

node95.members.0.js
146
genus
80

order
42
99.6429

family
5
97

node98.members.0.js
5
genus
97

family
33
100

33
node100.members.0.js
100
genus

family
100
4

4
node102.members.0.js
100
genus

order
100
101

100
101
family

101
node105.members.0.js
100
genus

68
98.8529
phylum

98.7794
68
class

order
89
5

87
5
family

node110.members.0.js
5
genus
80

99.3333
63
order

100
56
family

100
genus
21
node113.members.0.js

node114.members.0.js
35
genus
100

93
7
family

7
node116.members.0.js
93
genus

phylum
99.5838
50599

class
648
93

order
648
93

family
93
648

node121.members.0.js
648
genus
93

98.7897
3329
class

3329
98.7897
order

99.4527
433
family

genus
99.4527
node125.members.0.js
433

family
100
2434

2426
node127.members.0.js
99.9835
genus

80
genus
8
node128.members.0.js

90.5195
462
family

458
node130.members.0.js
80
genus

4
node131.members.0.js
83
genus

13261
99.2393
class

100
6032
order

100
6032
family

6032
node135.members.0.js
100
genus

6472
99.4192
order

family
80
2373

2373
node138.members.0.js
80
genus

121
100
family

genus
99
node140.members.0.js
3

genus
100
node141.members.0.js
118

family
30
99.6333

30
node143.members.0.js
99.6333
genus

family
91
4

genus
91
node145.members.0.js
4

family
7
93

7
node147.members.0.js
80
genus

100
823
family

100
genus
35
node149.members.0.js

genus
98
node150.members.0.js
218

genus
97
node151.members.0.js
507

63
node152.members.0.js
99
genus

89.2245
147
family

genus
89.2245
node154.members.0.js
147

2967
97.5541
family

81
node156.members.0.js
89.1975
genus

2886
node157.members.0.js
95.7696
genus

655
80
order

family
80
655

genus
80
node160.members.0.js
655

100
5
order

100
5
family

5
node163.members.0.js
99
genus

43
100
order

43
100
family

43
node166.members.0.js
100
genus

order
54
100

family
100
54

54
node169.members.0.js
100
genus

class
6
100

6
100
order

family
3
99

3
node173.members.0.js
99
genus

family
3
100

genus
100
node175.members.0.js
3

class
98.3391
6491

order
80
8

family
8
80

node179.members.0.js
8
genus
80

3
93
order

family
3
93

93
genus
3
node182.members.0.js

order
5027
98.6622

6
100
family

6
node185.members.0.js
80
genus

family
80
262

node187.members.0.js
262
genus
80

536
95.7705
family

4
node189.members.0.js
80
genus

node190.members.0.js
532
genus
94.8139

230
91.7391
family

80
genus
215
node192.members.0.js

88
genus
15
node193.members.0.js

99.9649
3993
family

node195.members.0.js
3993
genus
99.9649

order
94.1831
1453

94.1831
1453
family

1453
node198.members.0.js
94.1831
genus

1416
92.9718
class

100
23
order

100
23
family

node202.members.0.js
7
genus
80

16
node203.members.0.js
97
genus

order
99.4483
29

9
100
family

node206.members.0.js
2
genus
100

80
genus
2
node207.members.0.js

genus
84
node208.members.0.js
5

8
80
family

node210.members.0.js
8
genus
80

100
12
family

genus
100
node212.members.0.js
12

92.7038
1364
order

93
1246
family

genus
93
node215.members.0.js
1246

family
90.6111
90

genus
83
node217.members.0.js
28

node218.members.0.js
11
genus
80

51
node219.members.0.js
94.3529
genus

family
28
80

genus
80
node221.members.0.js
28

15
80
class

order
80
15

family
15
80

15
node225.members.0.js
80
genus

2221
98.2508
class

2221
98.2508
order

98.2508
2221
family

genus
98.2508
node229.members.0.js
2221

19079
98.7158
class

order
3502
98.3958

90.1066
2196
family

genus
85.1758
node233.members.0.js
546

80
genus
332
node234.members.0.js

95
genus
31
node235.members.0.js

genus
98.1111
node236.members.0.js
306

node237.members.0.js
981
genus
84.0102

family
80
170

80
genus
170
node239.members.0.js

family
1126
99.9538

node241.members.0.js
412
genus
80

714
node242.members.0.js
96.8487
genus

family
100
10

node244.members.0.js
10
genus
80

order
100
24

21
96
family

96
genus
21
node247.members.0.js

family
80
3

80
genus
3
node249.members.0.js

order
99.9907
4609

2594
100
family

node252.members.0.js
2594
genus
96

family
99.6811
577

99.6811
genus
577
node254.members.0.js

family
99.9145
1438

1147
node256.members.0.js
100
genus

291
node257.members.0.js
92.5636
genus

96.7037
54
order

family
96.7037
54

node260.members.0.js
44
genus
88.7273

genus
80
node261.members.0.js
10

order
100
59

59
100
family

59
node264.members.0.js
100
genus

7919
99.3796
order

6674
99.2092
family

node267.members.0.js
5
genus
83

98.2805
genus
1768
node268.members.0.js

node269.members.0.js
4346
genus
80

genus
80.8571
node270.members.0.js
280

genus
91.7757
node271.members.0.js
272

genus
100
node272.members.0.js
3

family
80
99

genus
80
node274.members.0.js
99

315
99.8667
family

node276.members.0.js
315
genus
99.1556

family
214
100

93.7069
genus
116
node278.members.0.js

node279.members.0.js
94
genus
86

genus
100
node280.members.0.js
4

100
248
family

genus
100
node282.members.0.js
248

90.7255
102
family

genus
90.7255
node284.members.0.js
102

family
265
100

genus
100
node286.members.0.js
6

100
genus
259
node287.members.0.js

100
2
family

genus
100
node289.members.0.js
2

order
98.8126
523

80
12
family

80
genus
12
node292.members.0.js

family
99.2074
511

511
node294.members.0.js
80
genus

99.8883
967
order

99.8883
967
family

genus
99.0252
node297.members.0.js
119

100
genus
153
node298.members.0.js

node299.members.0.js
6
genus
80

node300.members.0.js
689
genus
95

order
80
1416

family
80
1416

node303.members.0.js
1416
genus
80

order
6
100

6
100
family

100
genus
6
node306.members.0.js

4114
80
class

order
80
4114

family
4114
80

4114
node310.members.0.js
80
genus

class
100
16

100
16
order

16
100
family

genus
100
node314.members.0.js
11

80
genus
3
node315.members.0.js

2
node316.members.0.js
90
genus

class
86
3

order
3
86

86
3
family

3
node320.members.0.js
86
genus

phylum
20
99.75

class
99.75
20

order
99.75
20

99.75
20
family

node325.members.0.js
20
genus
99.5
